# Mono‐Palmitoyl‐N‐Alkylurea Ligands as Specific Activators of Human Toll‐Like Receptor 2/6 Heterodimer

**DOI:** 10.1002/cbic.202400583

**Published:** 2024-11-11

**Authors:** Marjolein M. E. Isendoorn, Giulia Castello, Çağla Koç, Nico Meeuwenoord, Jeroen D. C. Codée, Ferry Ossendorp, Dmitri V. Filippov

**Affiliations:** ^1^ Leiden Institute of Chemistry Leiden University Einsteinweg 55 2333 CC Leiden The Netherlands; ^2^ Department of Immunology Leiden University Medical Center Albinusdreef 2 2333 ZA Leiden The Netherlands

**Keywords:** Lipopeptides, Selective TLR2/6 ligands, Solid phase synthesis, Immunostimulants, Mini-Upam, Amino acids

## Abstract

Ligands for Toll‐like‐receptor 2 (TLR2) have demonstrated significant potential as immune‐stimulating components in synthetic vaccines. Activation of TLR2 relies on the formation of dimeric complexes with either TLR1 or TLR6 and the nature of these dimers can impact therapeutic outcomes. The lipopeptide‐based TLR2 ligands Pam_3_CysSK_4_ and Pam_2_CysSK_4_ have been extensively studied, and their recognition by different TLR‐receptor heterodimers, TLR2/TLR1 and TLR2/TLR6, respectively, has been established. However, the high lipophilicity of these ligands, containing multiple palmitoyl residues, can result in solubility issues when used as vaccine adjuvants. To address this, we previously synthesized a less lipophilic ligand containing a single palmitoyl chain called mini‐UPam, which effectively stimulates human moDC maturation. We here probe the receptor‐dimer specificity of several mini‐Upam derivatives and reveal that these mini‐UPam are hTLR2/TLR6 selective ligands and that the introduction of longer urea alkyl chains does not shift the binding specificity to hTLR2/TLR1 heterodimers, in contrast to their Pam_2_CysSK_4_ and Pam_3_CysSK_4_ counterparts, pointing to a different binding mode of the UPam ligands.

## Graphical abstract

The derivatives of the synthetic hTLR2 agonist mini‐UPam were synthesized and biologically evaluated to explore its TLR heterodimer specificity. Mini‐Upam is similar to the well‐known immunostimulatory lipopeptide Pam_2_CysSK_4_, which is a potential adjuvant for synthetic vaccines. Our research shows that mini‐UPam and its more lipophilic alkylurea derivatives require the heterodimer hTLR2/hTLR6, rather than hTLR2/hTLR1, to exert their immunostimulatory activity.

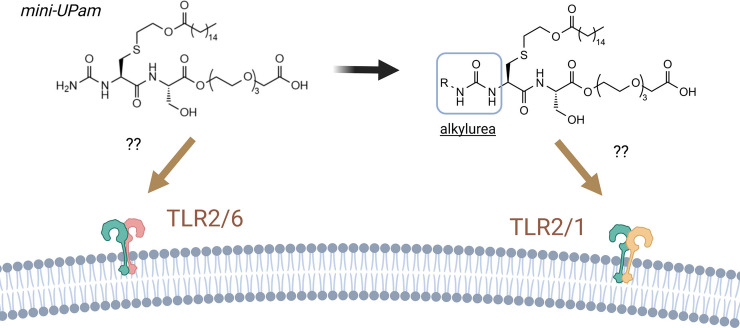

## Introduction

**Figure 1:**
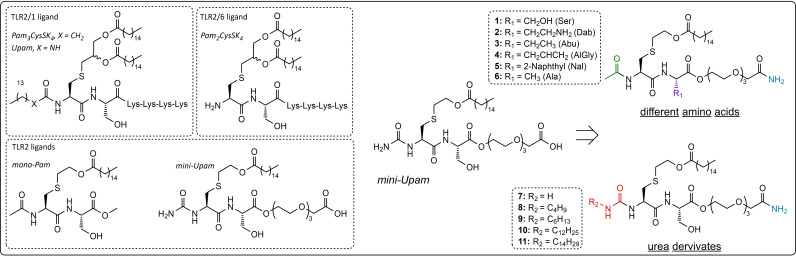
Overview of previously reported TLR2 ligands (Left Panel). Schematic overview of this work: design of new mini‐Upam derivatives (Right Panel). Colors indicate the residues modified in comparison to previous studies.

Lipopeptides that are part of the bacterial cell wall are recognized by the pattern recognition receptor Toll‐like‐receptor 2 (TLR2), which is present on antigen‐presenting cells. TLR2, once activated, initializes a signaling cascade that leads to the release of pro‐inflammatory cytokines and the initiation of the innate immune response.[^1^, ^2^, ^3^] Because of its ability to drive inflammatory responses, TLR2 is an attractive target for adjuvants, which are necessary components of therapeutic vaccines.[^4^, ^5^, ^6^, ^7^] TLR2 forms heterodimers with either TLR1 or TLR6. It primarily recognizes tri‐ and di‐acetylated lipopeptides, with the tri‐acylated lipopeptide Pam_3_CysSK_4_ and diacylated Pam_2_CysSK_4_ (Figure 1) being the most studied TLR2 ligands. Pam_3_CysSK_4_ is derived from the bacterial lipoprotein of *Escherichia coli* and immunology studies revealed that it is selective towards TLR2/TLR1 complex.^[8]^ On the other hand, Pam_2_CysSK_4_, which lacks the N‐acylated chain, is derived from a lipopeptide of *Mycoplasma fermentans* and has been found to be a TLR2/TLR6 ligand.^[9]^

Pam_3_Cys and Pam_2_Cys‐based ligands are highly lipophilic, making their use as adjuvants challenging due to poor solubility, especially when coupled to lipophilic antigenic peptides. Therefore, much effort has been directed toward developing a less lipophilic agonist of TLR2. Through structure‐activity relationship studies, David and co‐workers discovered a less lipophilic TLR2 ligand containing only one palmitoyl chain, which proved to be a ligand for human TLR2 (hTLR2) but not for that of mice.[^10^, ^11^, ^12^] This mono‐palmitoylated dipeptide (mono‐Pam) was further optimized by our group, culminating in the development of mini‐UPam (Figure 1), a derivative containing a urea moiety instead of the N‐terminal acetyl as in the original mono‐Pam and a tri(ethylene glycol) C‐terminal extension. The urea modification was inspired by our previous studies on Pam_3_CysSK_4_‐like TLR2 ligands, which revealed that substituting the original N‐palmitoyl at the α‐amino group of the terminal cysteine residue for an alkylurea residue led to a more potent ligand, so‐called UPam (Figure 1).^[13]^ To retain solubility and allow covalent attachment of antigenic peptides to the mini‐UPam ligand without loss of activity, the conventional tetra‐lysine linker (K_4_), present in Pam_3_Cys, Pam_2_Cys and Upam, was replaced by an ester‐bound tri(ethylene glycol) linker.^[14]^ Mini‐Upam shows an improved ability to mature human monocyte‐derived dendritic cells (moDCs), as measured by human IL12p40 production. We have recently shown the suitability of mini‐UPam as an adjuvant in conjugate vaccines, it was attached to the N‐termini of the two different peptide neoepitopes from a melanoma tumor. These mini‐UPam‐peptide conjugates demonstrated the capacity to trigger human TLR2 signaling in vitro, were able to functionally stimulate human DCs and enhanced the activation of neoantigen‐specific patient‐derived CD4^+^ or CD8^+^ T cells.^[14]^

The usefulness of TLR2 ligands as adjuvants depends on the binding preferences to either TLR2/1 or TLR2/6, as interaction with either complex can have different immunological effects. Both receptors operate via a MyD88 dependent signaling pathway, leading to inflammatory responses by which CD4^+^ T cells can differentiate to either Th1 or Th2 cells. It has previously been suggested that the immune response, induced by the activation of either the TLR2/TLR1 or TLR2/TLR6 heterodimers complexes, is similar and that the different dimers only serve to broaden the ligand repertoire.^[15]^ However, recent publications have provided new insights into these intracellular signaling routes, suggesting that they can differ depending on the nature of the activated heterodimer.[^16^, ^17^, ^18^, ^19^, ^20^] It was found that the strength of cellular activation upon ligand binding depends on the intracellular domains (ICDs) of the TLR1 or TLR6 coreceptors, where replacing the ICD of TLR1 with the one from TLR6 led to a reduced cytokine release upon lipopeptide ligation.^[16]^ Additionally, it was found in murine parasite vaccine models that Pam_2_CysSK_4_ is a Th2 polarizing adjuvant.[^21^, ^22^] Heinevetter and co‐workers studied the adjuvanticity of Pam_3_CysSK_4_ in vivo using the wheat storage protein gliadin as antigen, where they showed that the lipopeptide predominantly elicits the activation of the Th1 subset, as the IgG2a and IgA responses were enhanced but IgG1 production reduced.^[23]^ However, conflicting findings on the ability of different TLR2 heterodimer to skew Th1/Th2 immune responses have also been reported.[^6^, ^24^, ^25^]

The binding differences of Pam_3_CysSK_4_ and Pam_2_CysSK_4_ to the TLR2/TLR1 and TLR2/TLR6 dimer have been elucidated by co‐crystallization. The crystallographic data of TLR2/TLR1 with Pam_3_CysSK_4_ have revealed that two ester‐bound fatty acid chains are inserted into the TLR2 pocket in an extended conformation.^[26]^ The amide lipid chain interacts with TLR1, positioned in a narrow channel of the receptor. The cysteine backbone is located in the center where the TLR2 and TLR1 pockets join. Co‐crystallization of TLR2/TLR6 with Pam_2_CysSK_4_ showed similar binding for the two ester‐bound chains, which are inserted into the TLR2 pocket. The lipid channel of TLR6, however, is blocked by two phenylalanine side chains, allowing binding by Pam_2_CysSK_4_ but preventing Pam_3_CysSK_4_ from binding.^[27]^ Besides these co‐crystallography, many studies have focused on the structure−activity relationship (SAR) of these lipopeptides to better understand the ligand‐receptor binding and determine structural requirements for TLR2 activation.[^28^, ^29^] The group of Jung^[30]^ synthesized and evaluated several Pam_3_Cys analogues carrying two to five amino acids. In comparison, the compounds that included the single amino acid, cysteine, and cysteine methyl ester showed only minimal activity, suggesting that the presence of a dipeptide is essential for the activity. Detailed binding studies for mini‐UPam have not been reported, but extrapolating from the data on the Pam_2_Cys and Pam_3_Cys derivatives, it is hypothesized that this ligand likely binds to the TLR2/TLR6 complex rather than the TLR2/TLR1 dimer. Here, we set out to analyze this by determining the binding specificity of a series of mini‐UPam derivatives in comparison to both Pam_3_CysSK_4_ and Pam_2_CysSK_4_. We probed different amino acid residues flanking the central cysteine as this had previously delivered more active UPam derivatives. We also probed the effect of the addition of (short) urea alkyl chains to transform the putative TLR2/TLR6 mini‐UPam ligand to a TLR2/TLR1 activator.

## Results and Discussion

### Synthesis of the TLR2 Ligands

**Scheme 1:**
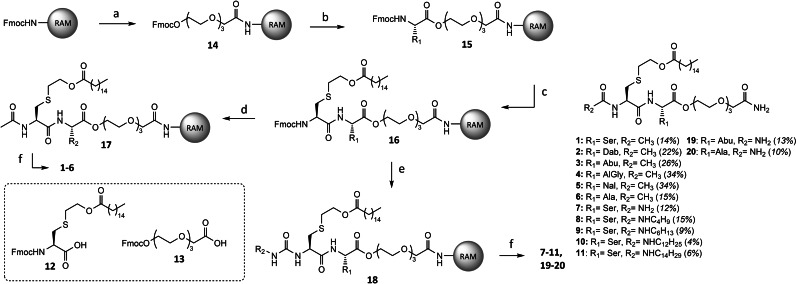
Synthesis of TLR2/6 ligands. Reaction conditions: a) i. 20 % piperidine in DMF ii. **13**, 1 M HCTU in DMF, 0.1 M DIPEA in DMF. b) i. 20 % piperidine in DMF ii. AA−X, 1 M HCTU in DMF, 0.1 M DIPEA in DMF. c) i. 20 % piperidine in DMF ii. **12**, 1 M HCTU in DMF, 0.1 M DIPEA in DMF. d) i. 20 % piperidine in DMF ii. 10 % Ac_2_O in DMF. e) i. 20 % piperidine in DMF ii. R‐isocyanate, i‐PrOH, DCM. f) 95 : 2.5 : 2.5 TFA/TIS/H_2_O.

Our previously developed mini‐UPam was used as the starting point for the new ligand design. Two types of derivatives were generated: in the first series, the serine residue was replaced by several other amino acids (Figure 1, 1–6), while the terminal amine was acetylated to simplify the synthesis of the ligands (green, Figure 1). In the second series, the N‐terminal amine was equipped with different alkyl‐containing urea substituents of increasing length (Figure 1, 7–11), which could influence the selectivity for binding to either the TLR2/TLR6 or TLR2/TLR1 heterodimer. The selection of the amino acids used was based on the outcome of our previously published study of UPam, where we had shown that small aliphatic amino acid side chains led to improved immunostimulatory activity.^[13]^ The best UPam derivatives were obtained by installing aminobutyric acid (Abu), diaminobutyric acid (Dab) and alanine (Ala). The bulky naphthylalanine (Nal) containing derivative **5** was added as a possible negative control since it was found that UPam‐Nal was inactive.^[13]^ The mini‐UPam derivatives were assembled by solid‐phase peptide synthesis (SPPS) using the known cysteine building block **12** and Fmoc‐protected triethylene glycol spacer (PEG‐3) **13** (Scheme 1).[^11^, ^14^] Rink amide MBHA (RAM) resin and a HCTU/piperidine Fmoc‐based peptide coupling protocol were used to install the linker, the different amino acid residues and the palmitoyloxyethyl cysteine building block. The RAM resin was selected to provide a C‐terminal amide upon cleavage, which mirrors the linkage of the ligand, when present in a peptide conjugate. Linker **13** was synthesized starting from commercially available triethylene glycol using tert‐butyl bromoacetate to install the carboxylic acid for conjugation purposes. After attachment of the amino acids and building block **12**, the terminal amine was deprotected with 20 % piperidine solution and either treated with acetic anhydride or the various isocyanates to obtain intermediates **17** and **18**. Through TFA‐assisted deprotection and cleavage from the resin, acetylated derivatives **1–6** and the alkylurea derivatives **7–11** were obtained.

### Immunological Evaluation of Mini‐UPam Derivatives

**Figure 2:**
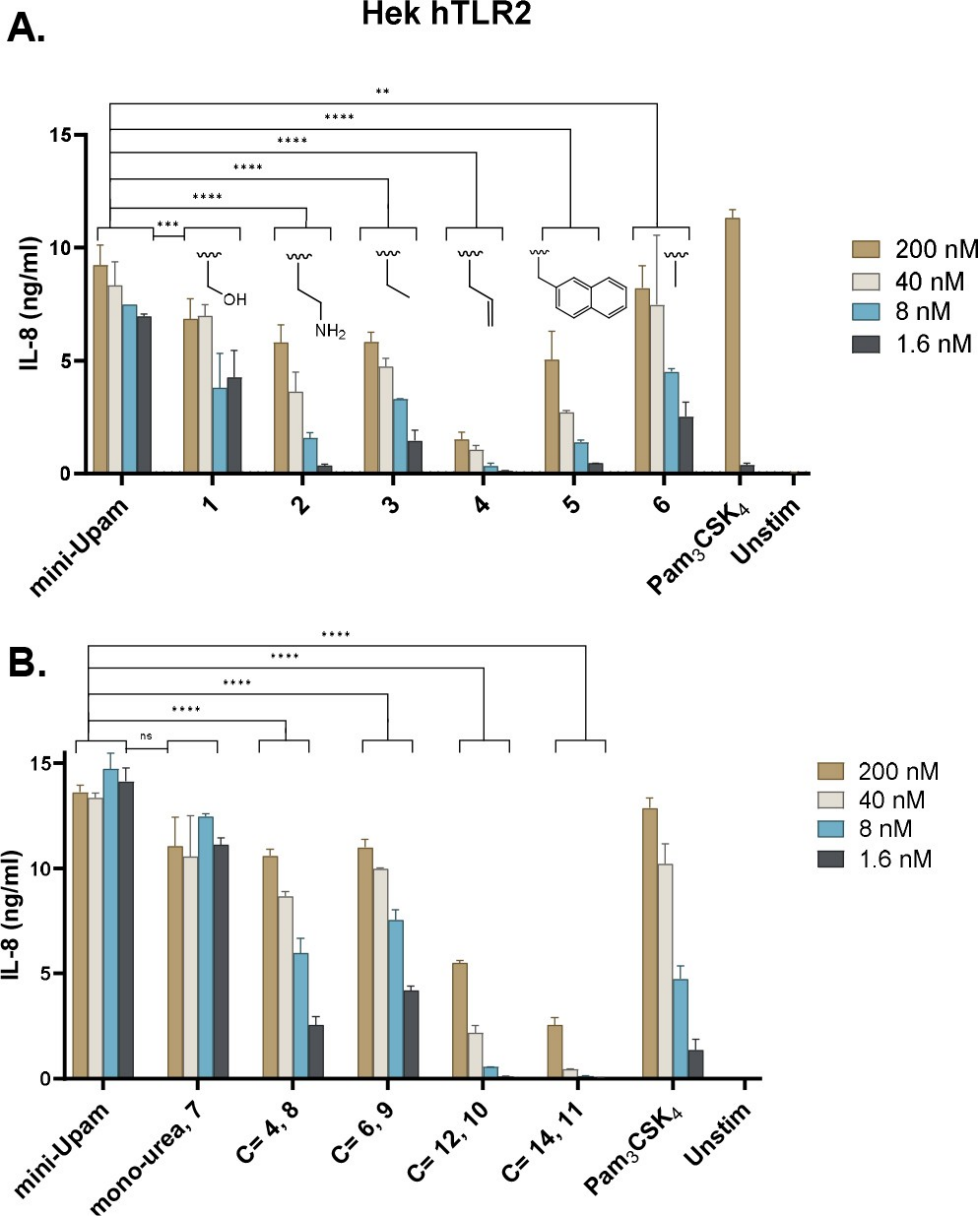
The activity of the synthesized mini ‐Pam and mini‐Upam derivatives measured by IL‐8 production at different concentrations in HEK293‐hTLR2 cells. A) The influence of different amino acids side chains on the serine position. B) The influence of N‐alkylurea length. Graphs are a representative image of n=3 independent experiments.

To evaluate the activity of the synthetic ligands for the TLR2 receptor, the human embryonic kidney cell line HEK293, stably transfected with human TLR2, the murine bone‐marrow derived dendritic cells (BMDCs) and a murine dendritic cell line were used. The cell lines were incubated with either the N‐acetyl mini‐Pam analogues (**1–6**) or the mini‐UPam derivatives containing different alkylureas with varying alkyl chain lengths (**7–11**). Murine dendritic cells showed no activity for any of the mini‐(U)Pam derivatives (Supp Figure S1). Using the human TLR2 transfected HEK293 cell line we observed that mini‐UPam outperformed other TLR2 ligands, Pam_3_CysSK_4_ and Pam_2_CysSK_4_, from which Pam_3_CysSK_4_ was selected as a positive control for the following experiments (Supp Figure S2). With Hek hTLR2 cells (Figure 2), it became apparent that the small and aliphatic side chains in **3**, **4** and **6** and the polar side chain in **2** did not outperform the N‐acetylated serine mini‐Pam **1** (mini‐Pam‐Ser). It is noteworthy that, in contrast to what we observed previously in our UPam study, mini‐Pam‐Nal **5** with bulky naphthyl alanine is able to activate hTLR2 and that compound **4** (mini‐Pam‐AlGly) was less active when compared to mini‐Pam‐Ser than its UPam counterpart (UPam‐AGly) was in comparison to UPam‐Ser.^[13]^ The crystallographic study by Kang *et al*. showed that the serine in Pam_2_CysSK_4_ fits in the narrow neck area of the ligand‐binding pocket and can form a hydrogen bond with the backbone amide of phenylalanine F319 of hTLR6.^[27]^ In addition, it has been shown that in Pam_2_Cys analogues in which the serine in the peptide chain flanking the central cysteine is replaced with bulky hydrophobic aromatic substituents, the activity of the ligands is preserved.[^31^, ^32^] Considering this, the reduced activity of compound **4** with its relatively small and hydrophobic side chain is unexpected. When comparing our prototype ligand mini‐Upam (mini‐Upam‐Ser) with the synthesized acetylated derivatives, it becomes apparent that the addition of the terminal urea increases the production of human IL‐8 (Figure 2A, mini‐UPam compared to **1**).

**Figure 3:**
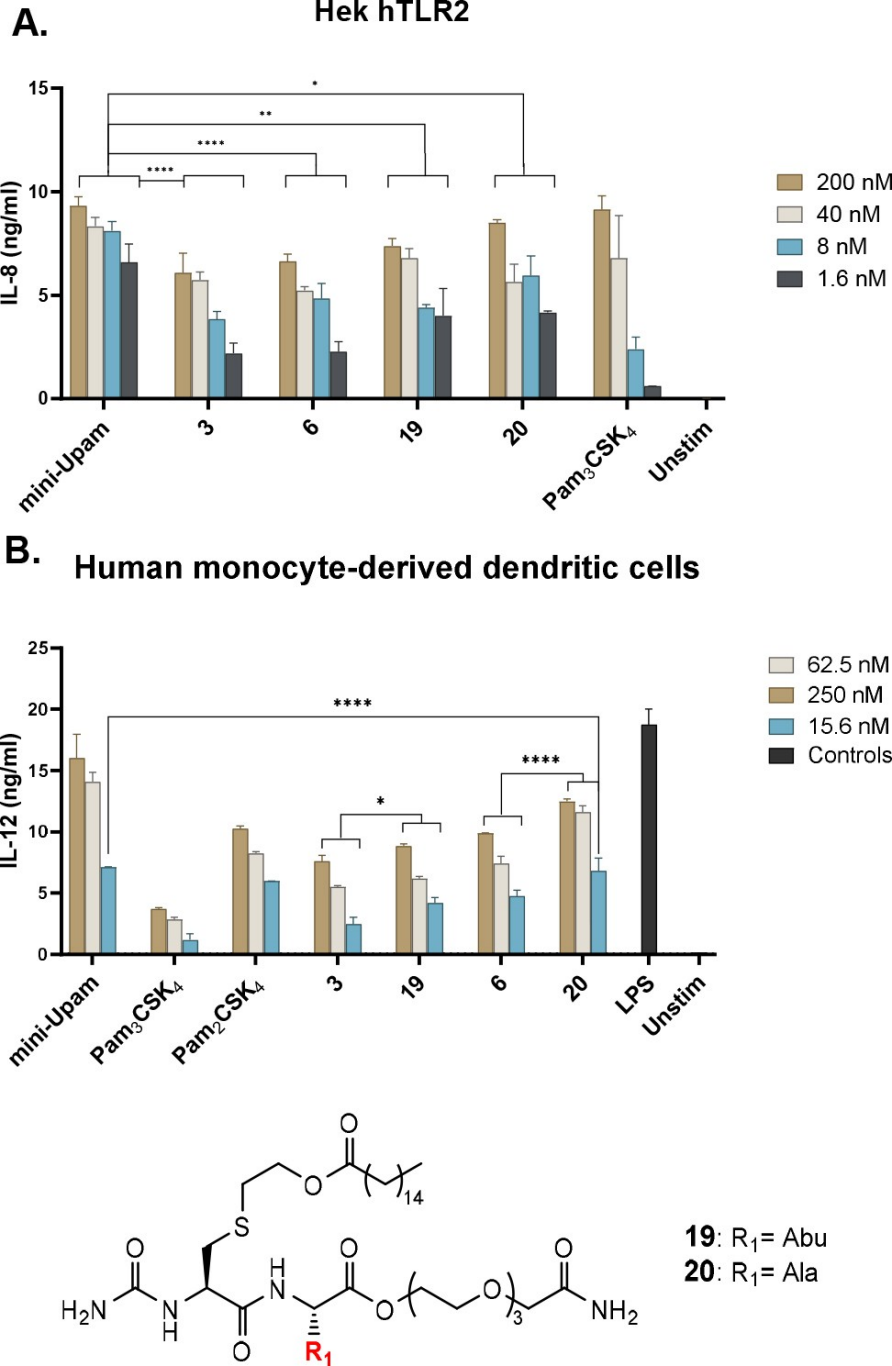
A: The activity of the acetylated derivatives **3** and **6** compared to their corresponding urea variants **19** and **21** measured by the IL‐8 production in Hek293‐hTLR2 cells. Graphs is a representative image of n=3 independent experiments. B: TLR2 activation of the acetylated derivatives **3** and **6** compared to their corresponding urea variants **19** and **20** measured by the IL‐12 production in human moDCs. Graph is a representative image of n=2 independent experiments. Statistical analysis was done via a two‐way ANOVA with Tukey's multiple comparison.

Subsequently, the different alkylurea ligands were tested, revealing that elongation of the alkylurea chain leads to diminished activity, and the non‐substituted urea **7** emerges as the most optimal residue when using both Hek293‐hTLR2 and human moDCs (Figure 2B and Supp Figure 3 respectively). Based on these results, we synthesized a new set of derivatives containing the optimal terminal urea residue and an Abu or Ala residue (mini‐UPam‐Abu **19** and mini‐UPam‐Ala **20**). These ligands both outperformed the N‐acetylated counterparts in Hek293‐hTLR2 and moDCs (Figure 3A and Figure 3B), with mini‐Upam‐Ala **20** with similar activity as mini‐Upam‐Ser at the lowest concentration.

### Selectivity Towards the Two Different Heterodimers (TLR2/TLR1 or TLR2/TLR6)

**Figure 4:**
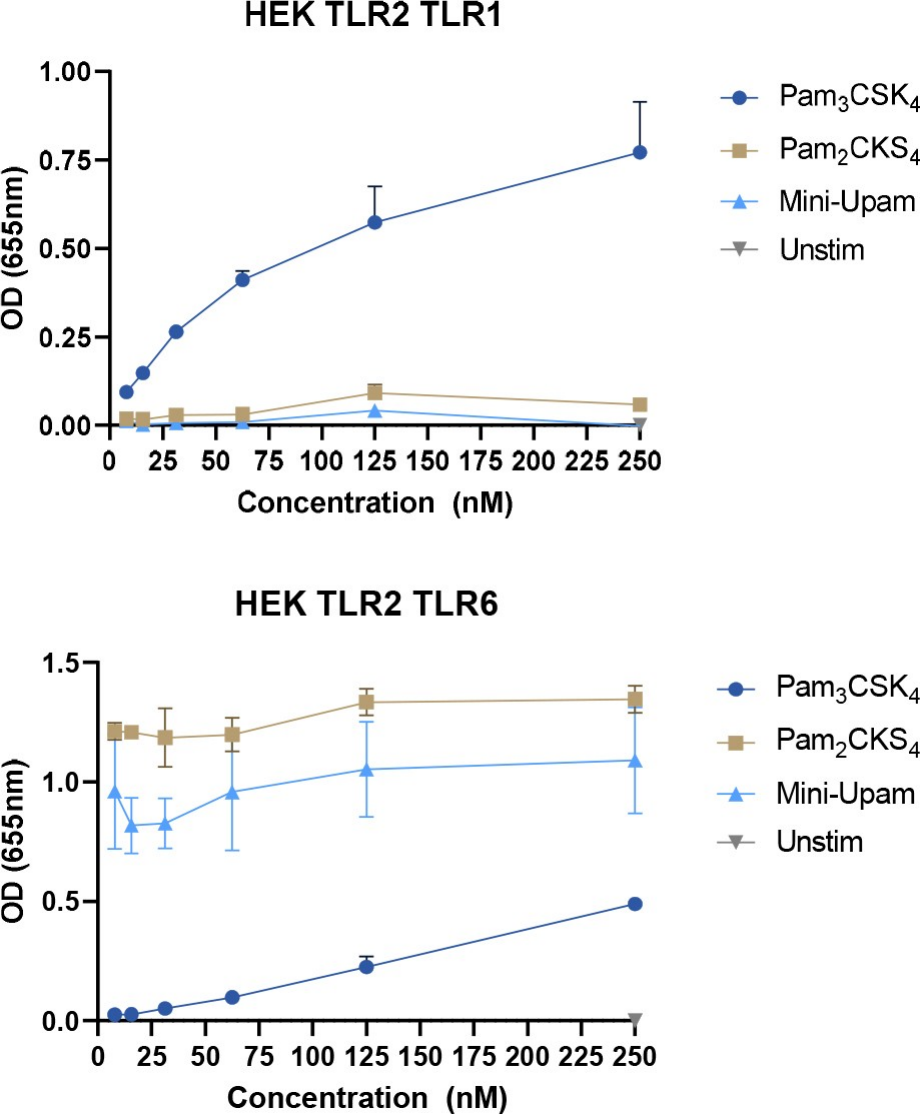
Binding selectivity of Pam_3_CysSK_4_, Pam_2_CysSK_4_ and mini‐UPam using knock‐in hTLR2/TLR1 or hTLR2/TLR6 HEK293 reporter cell line, co‐transfected with human TLR2 an NF‐ĸB/AP1‐inducible SEAP reporter genes (HEKblue TLR2 cells). The SEAP expression is measured at 655 nm with a spectrophotometer. Pam_3_CysSK_4_ is selective towards hTLR2/hTLR1. Pam_2_CysSK_4_ and mini‐UPam are selective for hTLR2/hTLR6. Graphs are a representative image of n=4 independent experiments.

**Figure 5:**
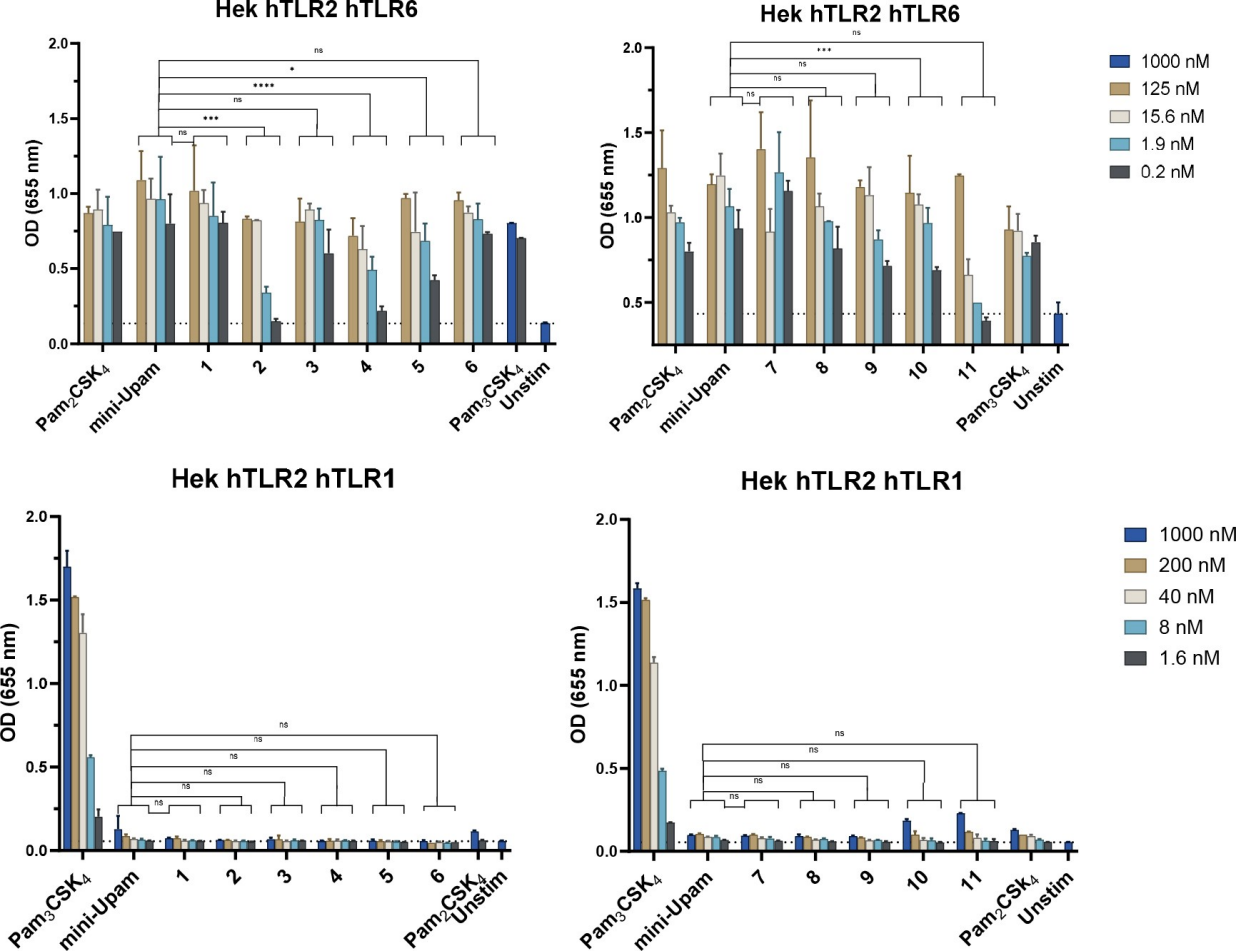
The binding specificity of the synthesized mini‐Upam **1**–**11** derivatives using knock‐in hTLR2/hTLR1 or hTLR2/hTLR6 HEK293 reporter cell line, co‐transfected with human TLR2 and NF‐ĸB/AP1‐inducible SEAP reporter genes (HEKblue). The SEAP expression is measured using a spectrophotometer at 655 nm using various concentrations. Graphs are a representative image of n=3 independent experiments.

To better understand the ligand binding to the two receptor heterodimers, the binding specificity was examined using a HEK293 reporter cell line with either knock‐out/knock‐in hTLR2/hTLR1 or hTLR2/hTLR6 co‐transfected with TLR2‐NF‐ĸB‐SEAP reporter genes (HEKblue). We first compared our prototype mini‐UPam (mini‐UPam‐Ser) with both Pam_3_CysSK_4_ and Pam_2_CysSK_4_ (Figure 4). It became apparent that mini‐UPam is unable to activate the hTLR2/hTLR1 heterodimer but does activate the hTLR2/hTLR6 dimer, indicating that mini‐UPam could bind similarly to Pam_2_CysSK_4_. Crystallographic studies suggest that elongation of the alkyl chain at the urea side might lead to shifting of the ligand selectivity from hTLR2/hTLR6 to hTLR2/hTLR1.[^27^, ^33^] However, derivatives **1**–**11** were all hTLR2/hTLR6 selective ligands (Figure 5), and increasing the alkyl urea chain did not result in a significant enhancement of the hTLR2/hTLR1 immunostimulatory activity. Only compounds **10** and **11** showed some activity at high concentrations (Figure 5). In addition, the hTLR2/hTLR6 activity diminishes upon elongation of the N‐alkylurea length. This suggests that the mini‐UPam compounds bind differently than Pam_3_CysSK_4_ and Pam_2_CysSK_4_.

## Conclusions

We have demonstrated that TLR2 ligands derived from mini‐UPam are selective for the hTLR2/hTLR6 heterodimer. To gain more insight into the activation of the TLR2/TLR6 complex, we synthesized multiple novel ligands derived from mini‐UPam. From previously reported crystallographic data, it has become clear that the length of the N‐acyl chain might determine which heterodimer is activated, as the presence of this third palmitoyl chain leads to selective TLR2/TLR1 recognition. In our study on the structure‐activity relationship, we found that the binding of mini‐Upam derivatives to the receptor differs from the prototype ligand Pam_2_CysSK_4_. Lengthening the alkylurea did not lead to a significant stimulation of the hTLR2/hTLR1 heterodimer, and the activation potency towards the hTLR2/hTLR6 heterodimer decreased somewhat with increasing length of the alkylurea chain. These data suggest that the alkyl substituent at the urea folds differently than the amide‐bound lipid chain, while the mini‐UPam derivative binds to the receptor heterodimer. Variation of the amino acid flanking the central cysteine has led to ligands **19** and **20**, with immunostimulatory activities similar to the original mini‐UPam (mini‐UPam‐Ser). These novel human TLR2/TLR6 selective ligands are able to mature human moDCs, as measured by human IL‐12 production in which mini‐Upam‐Ala **20** outperformed mini‐Upam‐Abu **19**. Replacing the terminal amine with an urea increased the potency of both Pam‐Cys derivatives (UPam and mini‐UPam), presumably by enabling the formation of an extra hydrogen bond between the receptor and the ligand. In the future, X‐ray studies may provide insight into the different ligand‐receptor interactions.

## Conflict of Interests

The authors declare no conflict of interest.

1

## Supporting information

As a service to our authors and readers, this journal provides supporting information supplied by the authors. Such materials are peer reviewed and may be re‐organized for online delivery, but are not copy‐edited or typeset. Technical support issues arising from supporting information (other than missing files) should be addressed to the authors.

Supporting Information

## Data Availability

The data that support the findings of this study are available in the supplementary material of this article.
