# Supplementary material for: Mono‐Palmitoyl‐N‐Alkylurea Ligands as Specific Activators of Human Toll‐Like Receptor 2/6 Heterodimer
Source: Chembiochem. 2024 Nov 11;25(23):e202400583. doi: 10.1002/cbic.202400583 (PMC11610712; doi:10.1002/cbic.202400583)
Supplement: Supplementary file 1 — Supporting Information [file CBIC-25-e202400583-s001.pdf]

ChemBioChem

Supporting Information

Mono-Palmitoyl-N-Alkylurea Ligands as Specific Activators of Human Toll-Like Receptor 2/6 Heterodimer

Marjolein M. E. Isendoorn, Giulia Castello, Çağla Koç, Nico Meeuwenoord,
Jeroen D. C. Codée, Ferry Ossendorp,* and Dmitri V. Filippov*

Mono-palmitoyl-N-alkylurea ligands as specific activators of human Toll-like receptor 2/6 heterodimer

Marjolein M.E. Isendoorn+,[a] Giulia Castello+,[b] Jeroen D. C. Codée,[a] Ferry Ossendorp,[b] and Dmitri V. Filippov[a]**

[a] Leiden Institute of Chemistry, Leiden University, Einsteinweg 55, 2333 CC Leiden, The Netherlands

[b] G. Castello, Prof. Dr. F. Ossendorp, Department of Immunology, Leiden University Medical Center, Albinusdreef 2, 2333 ZA Leiden, The Netherlands

Corresponding Author

* Ferry Ossendorp − Department of Immunology, Leiden University Medical Center, 2333 ZA Leiden, The Netherlands; Email: F.A.Ossendorp@lumc.nl

* Dmitri V. Filippov − Bio-Organic Synthesis, Leiden Institute of Chemistry, Leiden University, 2333 CC Leiden, The Netherlands; Email: filippov@chem.leidenuniv.nl

Table of Contents

Supplementary figures 2

Supplementary procedures 4

General methods 4

General procedure for amino acid variation 4

General procedure for alkyl urea variation 4

Cell culture 10

Activation human TLR-2 transfected HEK cells 10

Dendritic cell maturation determination 10

Specificity of hTLR2/1 and hTLR2/6 heterodimers 11

Statistical analysis 11

NMR spectra for synthetic ligands 12

LCMS figures synthetic ligands 38

Supplementary figures

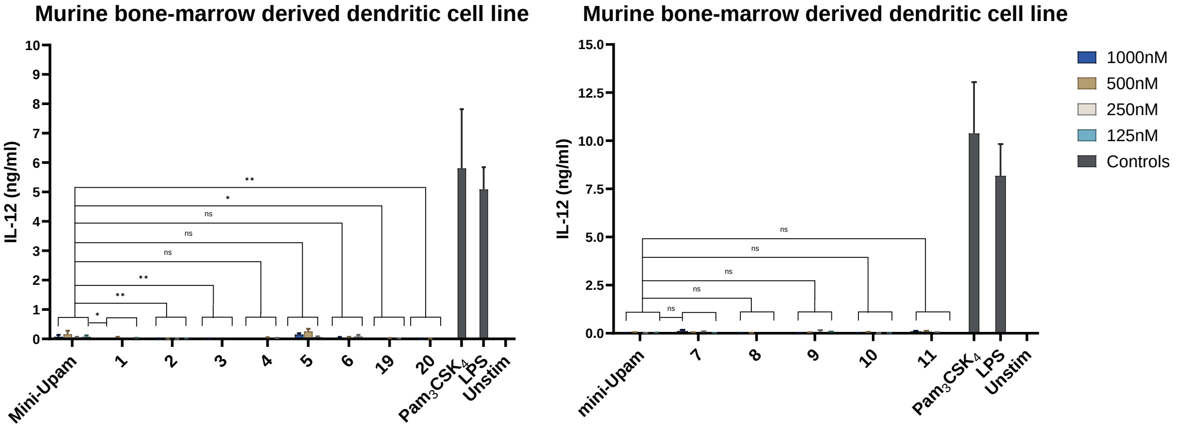

Figure S1: Analysis of activity of the synthesized mini-Pam (1-6) and mini-Upam (7-11, 19 and 20) derivatives measured by murine IL-12 production at different concentrations using A) murine D1 dendritic cell line and B) murine bone-marrow derived dendritic cells (BMDCs). LPS and Pam_3CSK_4 are used as positive controls at 2 µg/ml and 1000 nM respectively. Graphs are a representative image of n=4 independent experiments.

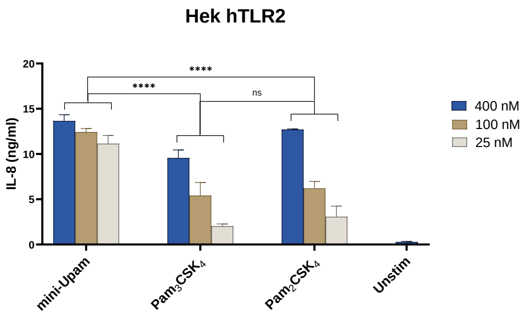

Figure S2: The activity of mini-Upam, Pam_3CSK_4, and Pam_2CSK_4 measured by human IL-8 production at different concentrations in HEK293-hTLR2 cells. Graphs are a representative image of n=2 independent experiments.

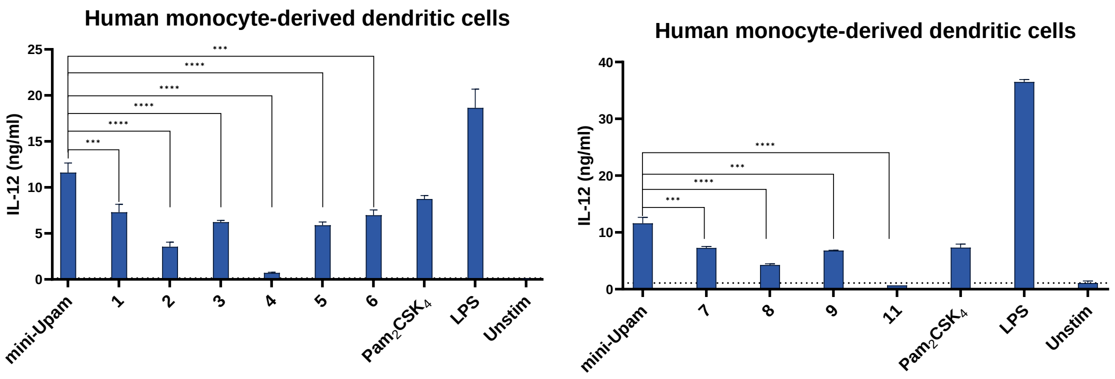

Figure S3: The activity of the synthesized mini-(U)pam derivatives at 1000 nM measured by human IL-12 production. LPS is used as positive control at 2µg/ml. A) The influence of different amino acid side chains on the serine position B) The influence of N-alkylurea length. Graphs are a representative image of n=2 independent experiments. Statistical analysis utilized was one-way ANOVA with Dunnett’s multiple comparisons.

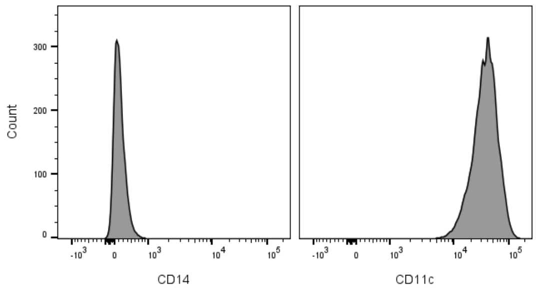

Figure S4: Quality control of human monocyte dendritic cells. Expression of CD14 and CD11c, displayed by flow cytometry, in live moDCs after 5 days of culture with human IL-4 and GM-CSF. The differentiation of $CD14^+$ monocytes into human moDCs is indicated by the loss of CD14 and gained expression of CD11c.

Supplementary procedures

General methods:

All reagents and solvents used in the solid-phase peptide synthesis were purchased from Biosolve (Netherlands). Fmoc amino acid building blocks were purchased from Sigma Aldrich or Novabiochem. was purchased from Sigma Aldrich. The solid-phase peptide synthesis was performed on the Liberty Blue™ Automated Microwaved Peptide Synthesiser (Gyros protein Technologies AB, Arizona, USA) using Rink amide MBHA resin LL (100-200 mesh) on a 50/100 µmol scale applying established Fmoc protocols. LC-MS analyses were executed on a Thermo Finnigann LCQ Fleet MAX ion-trap mass spectrometer with an electrospray ion source coupled to a Vanquish UHPLC system (Thermo Finnegan) or an Agilent Technologies 1260 Infinity LC system (detection simultaneously at 214 and 254 nm) coupled to a Agilent Technologies 6120 Quadrupole MS, using a diphenyl column (Vydac 219 TP, 5µm, 250 x 4.6 mm), using the following buffers: A: H_2O B: MeCN and C: 1% TFA in H_2O (0.1% TFA end concentration). High resolution mass spectra (HRMS) were recorded by direct injection (2 µl of a 1 µM solution in MeCN) using a Q-Exactive HF Orbitrap (Thermo Scientific) equipped with an electrospray ion source (ESI). Source voltage 3.5 kV, sheath gas flow 10, capillary temperature 250˚C with resolution R = 60.000 at m/z 400 (mass range m/z = 150 – 3,000 to a maximum of 6000). The HRMS was calibrated prior to measurements with a calibration mixture (Thermo Finnigan). Crude products were purified by preparative high-pressure liquid chromatography (HPLC) using etiher a Waters auto purifier prep LCMS coupled to a waters SQ detector, a Gilson GX281 with an automatoc fraction collector, or an Agilent1200 semi-prep system coupled to an Agilent 6120 quadruple detector. Columns used: Gemini-NX 5µm C18, 110 Å, 250 x 10.0 mm, 5 mL/min or Vydac 219TP 5µm Diphenyl, 300 Å, 250 x 10 mm, 5 mL/min in combination with with eluents A: 0.1% TFA in MilliQ in B: ACN or NUCLEOSIL 120-5 C4, 120 Å, 250 x 4.6 mm, 5 mL/min in combination with eluents A: 50% MeOH in H_2O + 0.2% TFA B: 50% IPA in CH_3CN.

General procedure for amino acid variation

The manual peptide couplings were carried out using fritted reaction syringe equipped with a syringe cap. The syringe was shaken using a Heidolph Multi Reax vertexer set at 650 rpm. The resin (50 µmol) was swollen in DMF (5 mL) for 1.5h. The Fmoc deprotection was achieved by agitating the resin with 20% (v/v) piperidine in DMF (3x 4 mL for 3 min), after which the resin was washed with DMF (3x 4 mL). The Peg-spacer coupling was applied in three-fold excess. Generally, the Fmoc protected PEG spacer (0.30 mmol, 3.0 eq) was dissolved in 0.2 M HCTU in NMP (1.48 mL, 2.95 eq) in an Eppendorf tube and the resulting solution was transferred to the reaction syringe. Next, the Eppendorf tube was washed with 1.0 M DIPEA in DMF (0.6 mL, 6.0 eq) and subsequently the solution was transferred to the reaction syringe. The syringe was shaken over night at RT. The amino acid coupling was applied in five-fold excess. Generally, the Fmoc protected amino acid (0.25 mmol, 5.0 eq) was dissolved in 0.2 M HCTU in NMP (0.74 mL, 2.95 eq) in an Eppendorf tube and the resulting solution was transferred to the reaction syringe. Next, the Eppendorf tube was washed with 1.0 M DIPEA in DMF (0.5 mL, 10.0 eq) and subsequently the solution was transferred to the reaction syringe. The syringe was shaken over night at RT. The mini-Upam building block **12** coupling, applying three-fold excess. Generally, the Fmoc protected mini-Upam building block (0.3 mmol, 3.0 eq) was dissolved in 0.2 M HCTU in NMP (1.48 mL, 2.95 eq) in an Eppendorf tube and the resulting solution was transferred to the reaction syringe. Next, the Eppendorf tube was washed with 1 M DIPEA in DMF (0.6 mL, 12.0 eq) and subsequently the solution was transferred to the reaction syringe. The syringe was shaken for 2h at RT. Acetylation of the was achieved with 10% Ac2O in DMF (2x 5 mL, 5 and 10 min)

After the acetylation the resin was washed with DMF (3x 4 mL), DCM (3x 4 mL) and dried with N_2 flow. The crude product was cleaved off the resin using TFA/TIS/H_2O (95:2.5:2.5, 2 mL) by shaking the syringe for 2h. The cleavage solution was collected in a centrifuge tube containing 1:1 Et_2O:Pnt solution and stored over night at -40°C. The tubes were centrifuged, the solution was decantated and the pellets were dried over N_2 flow.

The Fmoc protected amino acids used for the amino acid variation: Ser(*t*Bu), Dab(Boc), Abu, AlGly, 2-NaI.

General procedure for alkyl urea variation

For the various alkyl urea derivatives coupling the PEG-, amino acid- and mini-Upam coupling steps were repeated as described earlier using 100 µmol resin. Generally for the isocyanate derivatives, the isocyanate (0.67 mmol, 6.67 eq) and isopropanol (1.32 mmol, 13.22 eq) were dissolved in anhydrous DCM (2 mL) and transferred to the reaction syringe and shake for 2.5h at RT. After urea coupling the resin was washed with DCM (3x 4 mL) and dried with N_2 flow. The crude product was cleaved off the resin using TFA/TIS/H_2O (95:2.5:2.5, 2 mL) by shaking the syringe for 2h. The cleavage solution was collected in a centrifuge tube containing 1:1 Et_2O: Pnt solution and stored over night at -40°C. The tubes were centrifuged, the solution was decantated and the pellets were dried over N_2 flow.

The used isocyanates for the alkyl urea variation: trimethylsilyl isocyanate, butyl isocyanate, hexyl isocyanate, dodecyl isocyanate, tetradecyl isocyanate.

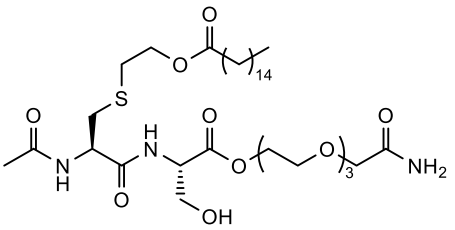

(14S, 17R)-17-acetamido-1-amino-14-(hydroxymethyl)-1, 13, 16-trioxo-3, 6, 9, 12-tetraoxa-19-thia-15-azahenicosan-21-yl palmitate (1)

Purification by HPLC provided **1** (5.2 mg, 7.2 µmol, 14%) as white solid. **LC-MS:** R_t= 11.724 (Phenomenex Gemini® 3 µm C_{18} 110 Å 50x4.6 mm, 10-90% MeCN, 10 min); **^{1}H NMR (850 MHz, $CDCl_3$):** δ 7.91 (d, 1H, *J* = 7.5 Hz), 7.42 (s, 1H), 6.90 (d, 1H, *J* = 7.7 Hz), 6.53 (s, 1H), 4.69 (q, 1H, *J* = 6.8 Hz), 4.63 – 4.56 (m, 1H), 4.50 – 4.45 (m, 1H), 4.31 – 4.17 (m, 3H), 4.09 – 3.99 (m, 3H), 3.89 (dd, 1H, *J* = 11.6, 3.4 Hz), 3.77 – 3.58 (m, 8H), 2.95 (dd, 1H, *J* = 6.4, 1.7 Hz), 2.87 – 2.75 (m, 2H), 2.32 (t, 2H, *J* = 7.6 Hz), 2.05 (s, 3H), 1.65 – 1.56 (m, 2H), 1.25 (s, 24H), 0.88 (t, 3H, *J* = 7.2 Hz); **^{13}C NMR (214 MHz, $CDCl_3$):** δ 174.42, 174.16, 170.92, 170.56, 70.94 , 70.32, 70.28, 70.17, 69.25, 64.46, 62.96, 62.42, 55.40, 52.75, 34.40, 34.37, 32.07, 31.03, 29.85, 29.84, 29.81, 29.81, 29.78, 29.64, 29.51, 29.44, 29.30, 25.04, 23.21, 22.84, 14.29; **HRMS:** $[M+H]^+$ calculated for $C_{34}H_{64}N_3O_{11}S$ 722.4256, found 722.4253.

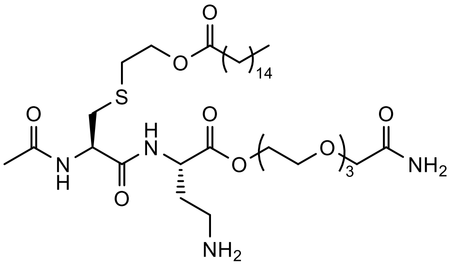

14S, 17R)-17-acetamido-1-amino-14-(2-aminoethyl)-1, 13, 16-trioxo-3, 6, 9, 12-tetraoxa-19-thia-15-azahenicosan-21-yl palmitate (2)

Purification by HPLC provided **2** (8.2 mg, 11.2 µmol, 22%) as brown solid. **LC-MS:** R_t= 10.956 (Phenomenex Gemini® 3 µm C_{18} 110 Å 50x4.6 mm, 10-90% MeCN, 10 min); **^{1}H NMR (850 MHz, $CDCl_3$)** δ 8.16, 7.51, 7.35, 6.99 (s, 1H), 4.72 (s, 2H, CH Cys), 4.59 (d, 1H, *J* = 7.1 Hz, CH Dab), 4.34 (s, 2H, CH_2 Peg), 4.25 – 4.19 (m, 2H, S-$CH_2C\mathit{H}_2$-O), 4.03 (s, 2H, CH_2 C-amide), 3.77 – 3.59 (m, 9H), 3.15 – 3.06 (m, 2H), 3.00 – 2.95 (m, 2H), 2.90 – 2.85 (m, 2H), 2.79 (t, 2H, *J* = 6.7 Hz, S-$C\mathit{H}_2CH_2$-O), 2.38 (s, 2H), 2.31 (t, 2H, *J* = 7.6 Hz), 2.04 (s, 3H), 1.63 – 1.58 (m, 3H), 1.34 – 1.20 (m, 24H), 0.88 (t, 3H, *J* = 7.1 Hz); **^{13}C NMR (214 MHz, $CDCl_3$)**: δ 174.51, 174.21, 174.14, 172.10, 171.93, 70.97, 70.36, 70.31, 70.23, 70.09, 68.95, 65.09, 62.85, 53.14, 50.14, 34.34, 33.58, 32.07, 31.00, 29.85, 29.82, 29.81, 29.79, 29.65, 29.52, 29.45, 29.31, 25.03, 24.89, 22.84, 14.29; **HRMS:** $[M+H]^+$ calculated for $C_{35}H_{67}N_4O_{10}S$ 735.4572, found 735.4569.

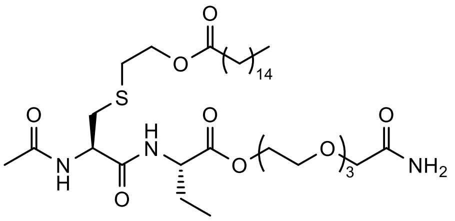

(14S, 17R)-17-acetamido-1-amino-14-ethyl-1, 13, 16-trioxo-3, 6, 9, 12-tetraoxa-19-thia-15-azahenicosan-21-yl palmitate (3)

Purification by HPLC provided **3** (9.3 mg, 12.9 µmol, 26%) as white solid. **LC-MS:** R_t= 12.277 (Phenomenex Gemini® 3 µm C_{18} 110 Å 50x4.6 mm, 10-90% MeCN, 10 min); **^{1}H NMR (850 MHz, $CDCl_3$):** δ 7.53 (s, 2H), 7.17 (d, 1H, *J* = 7.4 Hz), 7.08 (d, 1H, *J* = 7.7 Hz), 6.50 (s, 1H), 4.75 – 4.71 (m, 1H), 4.69 – 4.65 (m, 1H), 4.39 – 4.31 (m, 2H), 4.27 (t, 1H, *J* = 13.3, 6.7 Hz), 4.26 – 4.23 (m, 1H), 4.02 (s, 2H), 3.75 – 3.72 (m, 2H), 3.70 – 3.62 (m, 8H), 3.08 (dd, 1H, *J* = 13.9, 4.9 Hz), 3.01 (dd, 1H, *J* = 13.9, 6.6 Hz), 2.94 – 2.89 (m, 2H), 2.84 (t, *J* = 6.8 Hz, 1H), 2.77 (t, 1H, *J* = 6.7 Hz), 2.33 – 2.30 (m, 2H), 2.05 (d, 3H, *J* = 7.0 Hz), 1.62 – 1.59 (m, 2H), 1.25 (s, 24H), 0.88 (t, 3H, *J* = 7.2 Hz); **^{13}C NMR (214 MHz, $CDCl_3$)**: δ 174.09, 173.86, 171.99, 171.08, 170.84, 71.40, 70.55, 70.47, 70.40, 70.16, 69.16, 64.46, 62.86, 54.72, 52.37, 34.48, 34.36, 34.32, 33.98, 32.07, 31.16, 30.97, 29.85, 29.84, 29.81, 29.77, 29.63, 29.51, 29.44, 29.30, 25.02, 24.74, 22.99, 22.85, 14.29, 10.20; **HRMS:** $[M+H]^+$ calculated for $C_{35}H_{66}N_3O_{10}S$ 720.4463, found 720.4459.

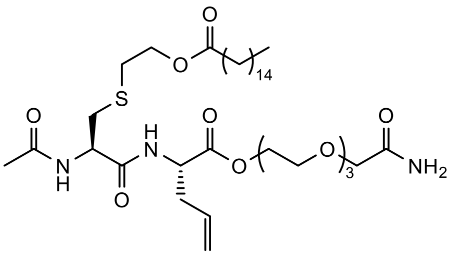

14S, 17R)-17-acetamido-14-allyl-1-amino-1, 13, 16-trioxo-3, 6, 9, 12-tetraoxa-19-thia-15-azahenicosan-21-yl palmitate (4)

Purification by HPLC provided **4** (12.3 mg, 16.8 µmol, 34%) as white solid. **LC-MS:** R_t= 12.419 (Phenomenex Gemini® 3 µm C_{18} 110 Å 50x4.6 mm, 10-90% MeCN, 10 min); **^{1}H NMR (850 MHz, CD_2Cl_2):** δ 7.66 (d, 1H, *J* = 6.9 Hz), 7.54 (s, 1H), 7.20 (d, 1H, *J* = 7.6 Hz), 6.45 (s, 1H), 5.80 – 5.70 (m, 1H), 5.19 – 5.09 (m, 2H), 4.66 – 4.60 (m, 1H), 4.46 (td, 1H, *J* = 7.6, 5.3 Hz), 4.38 – 4.31 (m, 1H), 4.26 – 4.14 (m, 3H), 3.74 – 3.59 (m, 7H), 2.91 (dd, 1H, *J* = 13.9, 6.6 Hz), 2.86 (dd, 1H, *J* = 13.8, 6.6 Hz), 2.78 (t, 2H, *J* = 6.8, 1.5 Hz), 2.61 – 2.56 (m, 1H), 2.53 – 2.48 (m, 1H), 2.30 (t, 2H, *J* = 7.7 Hz), 1.98 (s, 3H), 1.62 – 1.56 (m, 2H), 1.26 (s, 24H), 0.87 (t, 3H, *J* = 7.1 Hz); **^{13}C NMR (214 MHz, CD_2Cl_2)**: δ 174.62, 173.98, 171.86, 171.22, 170.97, 132.88, 119.35, 71.66, 70.70, 70.58, 70.52, 70.23, 69.49, 64.88, 63.25, 52.91, 52.84, 36.00, 34.48, 34.28, 32.31, 31.20, 30.08, 30.05, 30.04, 30.01, 30.00, 29.88, 29.74, 29.68, 29.51, 29.49, 25.27, 23.08, 23.03, 14.27; **HRMS:** $[M+H]^+$ calculated for $C_{36}H_{66}N_3O_{10}S$ 732.44634, found 732.46615.

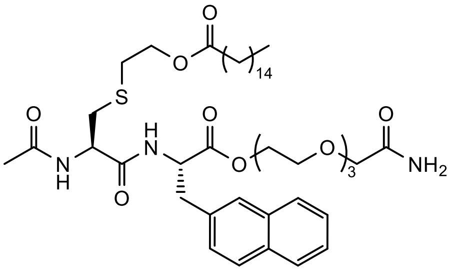

14S, 17R)-17-acetamido-1-amino-14-(naphtalen-2-ylmethyl)-1, 13, 16-trioxo-3, 6, 9, 12-tetraoxa-19-thia-15-azahenicosan-21-yl palmitate (5)

Purification by HPLC provided **5** (14.3 mg, 17.2 µmol, 34%) as white solid. **LC-MS:** R_t= 13.151 (Phenomenex Gemini® 3 µm C_{18} 110 Å 50x4.6 mm, 10-90% MeCN, 10 min); **^{1}H NMR (850 MHz, CD_2Cl_2)**: δ 8.09 (d, 1H, *J* = 8.4 Hz), 7.88 (d, 1H, *J* = 8.2 Hz), 7.78 (d, 1H, *J* = 8.1 Hz), 7.73 (d, 1H, *J* = 7.1 Hz), 7.58 – 7.53 (m, 1H), 7.51 (t, 1H, *J* = 7.1 Hz), 7.42 – 7.38 (m, 1H), 7.36 (s, 1H), 6.80 (d, 1H, *J* = 7.7 Hz), 6.41 (s, 1H), 4.79 – 4.73 (m, 1H), 4.54 – 4.49 (m, 1H), 4.28 (ddd, 1H, *J* = 12.1, 6.5, 2.6 Hz), 4.16 (m, 3H, *J* = 6.9 Hz), 3.72 – 3.65 (m, 2H), 3.65 – 3.59 (m, 4H), 3.59 – 3.53 (m, 2H), 3.45 (dd, 1H, *J* = 14.4, 8.4 Hz), 2.81 (d, 1H, *J* = 6.5 Hz), 2.74 – 2.68 (m, 2H), 2.30 – 2.25 (m, 2H), 1.92 (s, 3H), 1.61 – 1.54 (m, 2H), 1.26 (d, 24H, *J* = 19.1 Hz), 0.88 (t, 3H, *J* = 7.1 Hz); **^{13}C NMR (214 MHz, CD_2Cl_2)**: δ 174.43, 174.02, 171.62, 171.07, 170.59, 134.25, 132.90, 132.25, 129.24, 128.23, 128.16, 126.75, 126.15, 125.76, 123.77, 71.69, 70.75, 70.64, 70.56, 70.34, 69.26, 65.04, 63.11, 54.09, 52.64, 34.78, 34.47, 34.32, 32.31, 31.11, 30.08, 30.05, 30.04, 30.01, 29.88, 29.74, 29.69, 29.68, 29.51, 29.49, 25.26, 23.09, 23.08, 14.27; **HRMS:** $[M+H]^+$ calculated for $C_{44}H_{70}N_3O_{10}S$ 832.4776, found 832.4771.

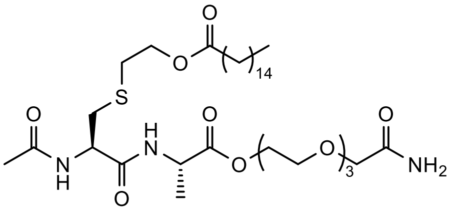

(14S,17R)-17-acetamido-1-amino-14-ethyl-1,13,16-trioxo-3, 6, 9, 12-tetraoxa-19-thia-15-azahenicosan-21-yl palmitate (6)

Purification by HPLC provided **6** (5.3 mg, 7.51 µmol, 15%) as white solid. **LC-MS:** R_t= 11.318 (Phenomenex Gemini® 3 µm C_{18} 110 Å 50x4.6 mm, 10-90% MeCN, 10 min); **^{1}H NMR (600 MHz, CD_2Cl_2):** δ 7.64 (d, *J* = 6.6 Hz, 1H), 7.48 (s, 1H), 6.90 (d, *J* = 7.9 Hz, 1H), 6.33 (s, 1H), 4.52 (dt, *J* = 7.9, 6.4 Hz, 1H), 4.35 – 4.28 (m, 1H), 4.28 – 4.22 (m, 1H), 4.18 – 4.10 (m, 3H), 3.89 (s, 2H), 3.65 – 3.47 (m, 11H), 2.86 – 2.76 (m, 2H), 2.72 (t, *J* = 6.7 Hz, 2H), 2.22 (t, *J* = 15.2, 7.3 Hz, 2H), 1.54 – 1.47 (m, 2H), 1.34 (d, *J* = 7.3 Hz, 3H), 1.18 (s, 25H), 0.80 (t, *J* = 7.0 Hz, 3H). **^{13}C NMR (151 MHz, CD_2Cl_2):** δ 174.41, 174.06, 172.76, 170.99, 170.55, 71.80, 70.80, 70.68, 70.65, 70.36, 69.48, 64.91, 63.19, 54.20, 54.02, 53.84, 53.66, 53.48, 52.70, 49.36, 34.62, 34.50, 32.31, 31.22, 30.09, 30.08, 30.06, 30.04, 30.02, 29.89, 29.75, 29.69, 29.52, 25.27, 23.17, 23.08, 17.28, 14.28; **HRMS:** $[M+H]^+$ calculated for $C_{34}H_{64}N_3O_{10}S$ 706.4307, found 706.4308.

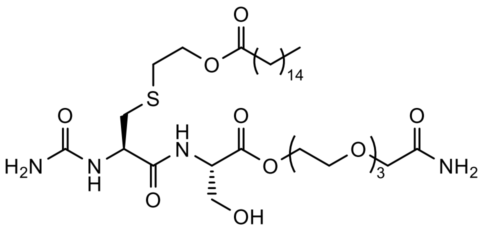

(14S, 17R)-1-amino-14-(hydroxymethyl)-1, 13, 16-trioxo-17-ureido-3, 6, 9, 12-tetraoxa-19-thia-15-azahenicosan-21-yl palmitate (7)

Purification by HPLC provided **7** (8.6 mg, 11.9 µmol, 12%) as white solid. **LC-MS:** R_t= 11.488 (Phenomenex Gemini® 3 µm C_{18} 110 Å 50x4.6 mm, 10-90% MeCN, 10 min); **^{1}H NMR (850 MHz, CD_2Cl_2)**: δ 7.82 (s, 1H), 7.49 (s, 1H), 6.75 (s, 1H), 6.39 (s, 1H), 5.24 (s, 2H), 4.61 – 4.50 (m, 2H), 4.47 – 4.42 (m, 1H), 4.20 (t, *J* = 5.6 Hz, 2H), 4.02 – 3.93 (m, 3H), 3.84 (d, *J* = 10.9 Hz, 1H), 3.73 – 3.57 (m, 9H), 3.01 – 2.90 (m, 2H), 2.82 – 2.74 (m, 2H), 2.29 (t, *J* = 7.6 Hz, 2H), 1.77 (s, 1H), 1.62 – 1.54 (m, 3H), 1.25 (s, 24H), 0.87 (t, *J* = 7.1 Hz, 3H). **^{13}C NMR (214 MHz, CD_2Cl_2):** δ 174.96, 174.23, 174.05, 172.18, 170.79, 159.34, 71.47, 70.80, 70.77, 70.71, 70.68, 70.57, 70.53, 70.50, 69.44, 69.35, 64.74, 63.41, 62.60, 55.80, 54.17, 35.10, 34.55, 32.34, 31.50, 30.11, 30.09, 30.07, 30.06, 29.93, 29.77, 29.73, 29.57, 25.31, 23.10, 14.29; **HRMS:** $[M+H]^+$ calculated for $C_{33}H_{63}N_4O_{11}S$ 723.4209, found 723.4202.

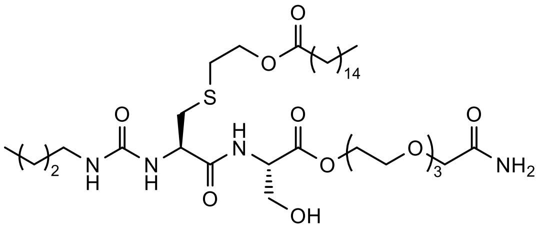

(14S, 17R)-1-amino-17-(3-butylureido)-14-(hydroxymethyl)-1, 13, 16-trioxo-3, 6, 9, 12-tetraoxa-19-thia-15-azahenicosan-21-yl palmitate (8)

Purification by HPLC provided **8** (11.4 mg, 14.6 µmol, 15%) as white solid. **LC-MS:** R_t= 12.265 (Phenomenex Gemini® 3 µm C_{18} 110 Å 50x4.6 mm, 10-90% MeCN, 10 min); **^{1}H NMR (850 MHz, CD_2Cl_2):** δ 7.91 (s, 1H), 7.43 (s, 1H), 6.76 (s, 1H), 6.09 (s, 1H), 5.52 (s, 1H), 4.56 – 4.53 (m, 1H), 4.53 – 4.50 (m, 1H), 4.46 – 4.41 (m, 1H), 4.23 (ddd, *J* = 12.2, 5.7, 2.5 Hz, 1H), 4.20 (t, *J* = 6.8 Hz, 2H), 3.99 (s, 2H), 3.96 (dd, *J* = 11.3, 4.0 Hz, 1H), 3.83 (dd, *J* = 11.6, 3.4 Hz, 1H), 3.73 – 3.65 (m, 4H), 3.65 – 3.58 (m, 5H), 3.18 – 3.07 (m, 2H), 2.97 – 2.90 (m, 2H), 2.78 (td, *J* = 6.8, 1.4 Hz, 2H), 2.32 – 2.28 (m, 2H), 1.59 (m, 2H), 1.47 – 1.41 (m, 2H), 1.36 – 1.31 (m, 2H), 1.26 (s, 28H), 0.91 (t, *J* = 7.4 Hz, 3H), 0.87 (t, *J* = 7.1 Hz, 3H); **^{13}C NMR (214 MHz, CD_2Cl_2)**: δ 174.76, 174.24, 172.66, 170.89, 158.55, 71.26, 70.60, 70.33, 69.44, 64.68, 63.38, 62.46, 55.75, 53.98, 40.39, 35.13, 34.51, 32.57, 32.31, 31.32, 30.08, 30.06, 30.04, 30.03, 29.90, 29.75, 29.70, 29.53, 25.28, 23.08, 20.36, 14.27, 13.98; **HRMS:** $[M+H]^+$ calculated for $C_{37}H_{71}N_4O_{11}S$ 779.4835, found 779.4831.

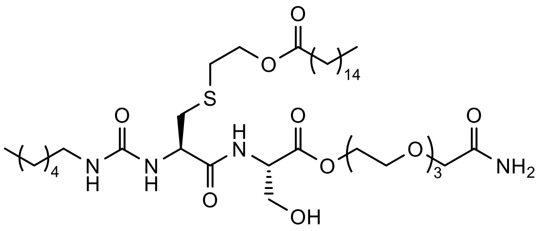

(14S, 17R)-1-amino-17-(3-hexylureido)-14-(hydroxy-methyl)-1, 13, 16-trioxo-3, 6, 9, 12-tetraoxa-19-thia-15-azahenicosan-21-yl palmitate (9)

Purification by HPLC provided **9** (7.6 mg, 9.4 µmol, 9%) as white solid. **LC-MS:** R_t= 12.265 (Phenomenex Gemini® 3 µm C_{18} 110 Å 50x4.6 mm, 10-90% MeCN, 10 min); **^{1}H NMR (850 MHz, CD_2Cl_2)**: δ 7.86 (d, *J* = 7.5 Hz, 1H), 7.41 (s, 1H), 6.70 (s, 1H), 6.03 (s, 1H), 5.46 (s, 1H), 4.56 – 4.52 (m, 1H), 4.52 – 4.49 (m, 1H), 4.47 – 4.43 (m, 1H), 4.24 – 4.19 (m, 2H), 3.99 (s, 2H), 3.97 (dd, *J* = 11.6, 4.1 Hz, 1H), 3.83 (dd, *J* = 11.7, 3.4 Hz, 1H), 3.73 – 3.65 (m, 4H), 3.65 – 3.59 (m, 5H), 3.17 – 3.07 (m, 2H), 2.97 (dd, *J* = 13.9, 6.6 Hz, 1H), 2.92 (dd, *J* = 13.9, 5.6 Hz, 1H), 2.81 – 2.75 (m, 2H), 2.30 (t, *J* = 7.5 Hz, 2H), 1.62 – 1.55 (m, 2H), 1.49 – 1.42 (m, 2H), 1.26 (s, 29H), 0.88 (td, *J* = 7.0, 5.0 Hz, 6H); **^{13}C NMR (214 MHz, CD_2Cl_2)**: δ 174.71, 174.23, 172.54, 170.90, 158.45, 71.26, 70.61, 70.57, 70.34, 69.47, 64.64, 63.37, 62.49, 55.78, 54.09, 40.73, 35.13, 34.51, 34.02, 32.31, 31.96, 31.36, 30.47, 30.08, 30.06, 30.04, 30.03, 30.01, 29.90, 29.88, 29.75, 29.71, 29.69, 29.53, 29.50, 26.95, 25.28, 23.08, 23.00, 14.27, 14.22; **HRMS**: $[M+H]^+$ calculated for $C_{39}H_{75}N_4O_{11}S$ 807.5148, found 807.5141.

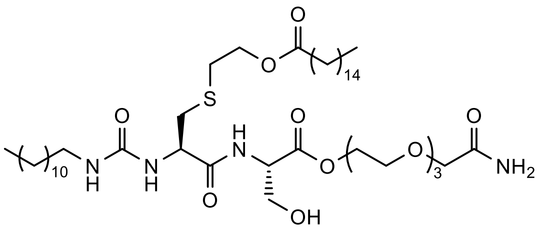

(14S, 17R)-1-amino-17-(3-dodecylureido)-14-(hydroxymethyl)-1, 13, 16-trioxo-3, 6, 9, 12-tetraoxa-19-thia-15-azahenicosan-21-yl palmitate (10)

Purification by HPLC provided **10** (3.1 mg, 3.5 µmol, 4%) as white solid. **LC-MS:** R_t= 12.656 (Phenomenex Gemini® 3 µm C_{18} 110 Å 50x4.6 mm, 10-90% MeCN, 10 min); **^{1}H NMR (850 MHz, CD_2Cl_2)**: δ 8.13 (d, *J* = 6.0 Hz, 1H), 7.11 (s, 1H), 6.89 (s, 1H), 6.10 (s, 1H), 5.53 (s, 1H), 4.59 – 4.52 (m, 2H), 4.48 (ddd, *J* = 12.5, 6.7, 2.6 Hz, 1H), 4.29 (ddd, *J* = 12.3, 5.8, 2.6 Hz, 1H), 4.26 – 4.20 (m, 2H), 4.00 (dd, *J* = 11.8, 4.2 Hz, 1H), 3.97 (s, 1H), 3.88 (dd, *J* = 11.7, 3.4 Hz, 1H), 3.79 – 3.60 (m, 9H), 3.18 (dd, *J* = 13.2, 6.4 Hz, 1H), 3.14 – 3.09 (m, 1H), 3.01 – 2.93 (m, 2H), 2.81 (t, *J* = 6.8 Hz, 1H), 2.81 – 2.76 (m, 1H), 2.36 – 2.31 (m, 2H), 1.66 – 1.60 (m, 3H), 1.52 – 1.47 (m, 2H), 1.30 (s, 32H), 0.92 (t, *J* = 7.1 Hz, 6H); **^{13}C NMR (151 MHz, CD_2Cl_2):** δ 175.25, 174.30, 174.28, 172.23, 171.30, 158.90, 72.07, 71.98, 71.89, 71.49, 71.46, 71.43, 71.35, 71.30, 71.15, 71.12, 71.06, 70.97, 70.20, 70.14, 70.11, 65.38, 65.29, 65.25, 62.92, 62.89, 62.46, 57.99, 57.75, 56.76, 54.79, 54.61, 54.43, 54.25, 54.07, 34.94, 34.93, 34.92, 32.94, 30.71, 30.69, 30.67, 30.67, 30.65, 30.52, 30.52, 30.47, 30.45, 30.38, 30.37, 30.33, 30.31, 30.31, 30.14, 27.99, 27.97, 25.78, 23.70, 14.88; **HRMS**: $[M+H]^+$ calculated for $C_{45}H_{87}N_4O_{11}S$ 891.6087, found 891.6084.

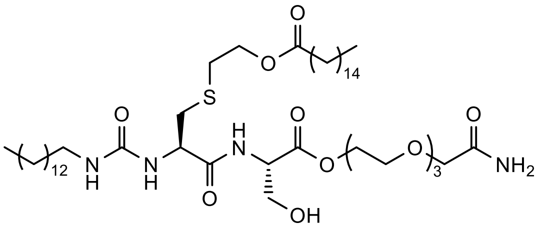

(14S,17R)-1-amino-14-(hydroxymethyl)-1,13,16-trioxo-17-(3-tetradecylureido)-3,6,9,12-tetraoxa-19-thia-15-azahenicosan-21-yl palmitate (11)

Purification by HPLC provided **11** (5.9 mg, 6.4 µmol, 6%) as white solid. **LC-MS:** R_t= 10.301 (Phenomenex Gemini® 3 µm C_{18} 110 Å 50x4.6 mm, 10-90% MeCN, 10 min); **^{1}H NMR (600 MHz, $CDCl_3$):** δ 8.52 (s, 1H), 8.03 (s, 1H), 6.82 (s, 1H), 6.07 (s, 1H), 5.53 (s, 1H), 4.57 (dt, *J* = 28.8, 5.0 Hz, 2H), 4.54 – 4.43 (m, 2H), 4.29 – 4.16 (m, 4H), 4.02 (s, 2H), 4.02 – 3.85 (m, 4H), 3.75 – 3.58 (m, 13H), 3.20 – 3.07 (m, 3H), 3.02 (dd, *J* = 13.9, 6.2 Hz, 2H), 2.98 – 2.86 (m, 2H), 2.85 – 2.71 (m, 3H), 2.31 (t, *J* = 7.5 Hz, 4H), 1.65 – 1.55 (m, 4H), 1.52 – 1.40 (m, 3H), 1.25 (s, 71H), 0.88 (t, *J* = 7.0 Hz, 11H); **^{13}C NMR (151 MHz, $CDCl_3$)**: δ 174.78, 173.88, 171.67, 170.84, 158.35, 71.53, 71.40, 71.29, 71.03, 70.97, 70.94, 70.85, 70.76, 70.70, 70.65, 70.58, 70.47, 69.82, 69.72, 69.66, 64.86, 64.74, 62.56, 62.50, 62.45, 62.00, 57.53, 57.23, 57.19, 41.57, 40.94, 34.51, 34.49, 32.47, 30.73, 30.67, 30.26, 30.24, 30.23, 30.21, 30.20, 30.18, 30.04, 29.98, 29.96, 29.90, 29.85, 29.83, 29.67, 27.50, 25.32, 25.31, 23.23, 14.51; **HRMS**: $[M+H]^+$ calculated for $C_{47}H_{91}N_4O_{11}S$ 919.6400, found 919.6404.

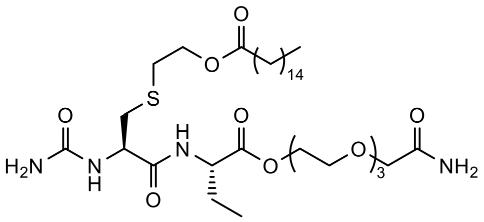

(14S,17R)-1-amino-14-ethyl-1,13,16-trioxo-17-ureido-3,6,9,12-tetraoxa-19-thia-15-azahenicosan-21-yl palmitate (19)

Purification by HPLC provided **19** (9.1 mg, 12.6 µmol, 13%) as white solid. **LC-MS:** R_t= 11.819 (Phenomenex Gemini® 3 µm C_{18} 110 Å 50x4.6 mm, 10-90% MeCN, 10 min); **^{1}H NMR (400 MHz, $CDCl_3$)**: δ 7.64 (s, 1H), 7.46 (d, *J* = 6.3 Hz, 1H), 7.21 (s, 1H), 6.79 (d, *J* = 7.7 Hz, 1H), 5.06 (s, 2H), 4.56 (q, *J* = 6.9 Hz, 1H), 4.48 – 4.39 (m, 1H), 4.34 – 4.28 (m, 2H), 4.28 – 4.15 (m, 1H), 4.09 – 3.93 (m, 2H), 3.81 – 3.61 (m, 8H), 3.03 (dd, *J* = 14.0, 5.4 Hz, 1H), 2.96 – 2.84 (m, 2H), 2.35 (t, *J* = 7.6 Hz, 2H), 1.91 (dq, *J* = 14.4, 7.7, 7.0 Hz, 1H), 1.81 (dt, *J* = 14.1, 7.4 Hz, 1H), 1.28 (s, 21H), 1.01 (t, *J* = 7.5 Hz, 2H), 0.91 (t, *J* = 6.8 Hz, 3H); **^{13}C NMR (214 MHz, $CDCl_3$):** δ 174.72, 174.14, 172.03, 171.73, 158.93, 77.16, 77.01, 76.86, 71.24, 70.55, 70.49, 70.12, 69.93, 69.43, 64.23, 62.81, 54.95, 53.08, 35.18, 34.27, 31.93, 31.11, 29.70, 29.69, 29.66, 29.63, 29.48, 29.37, 29.29, 29.16, 24.89, 24.38, 22.70, 14.13, 10.03; **HRMS:** $[M+H]^+$ calculated for $C_{34}H_{65}N_4O_{10}S$ 721.4416, found 721.4433.

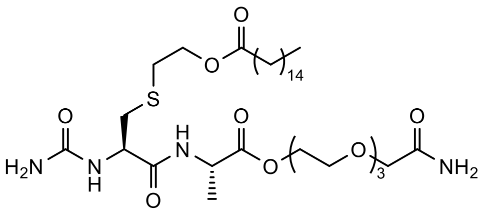

(14S,17R)-1-amino-14-methyl-1,13,16-trioxo-17-ureido-3,6,9,12-tetraoxa-19-thia-15-azahenicosan-21-yl palmitate (20)

Purification by HPLC provided **20** (7.2 mg, 10.2 µmol, 10%) as white solid. **LC-MS:** R_t= 11.636 (Phenomenex Gemini® 3 µm C_{18} 110 Å 50x4.6 mm, 10-90% MeCN, 10 min); **^{1}H NMR (400 MHz, $CDCl_3$)**: δ 7.60 (s, 1H), 7.54 (d, *J* = 6.0 Hz, 1H), 7.12 (s, 1H), 6.79 (d, *J* = 7.9 Hz, 1H), 5.07 (s, 2H), 4.51 (q, *J* = 6.7 Hz, 1H), 4.43 – 4.32 (m, 2H), 4.29 – 4.18 (m, 3H), 3.98 (q, 2H), 3.77 – 3.60 (m, 10H), 2.99 (dd, *J* = 14.0, 5.7 Hz, 1H), 2.91 – 2.80 (m, 3H), 2.32 (t, *J* = 7.6 Hz, 2H), 1.61 (t, *J* = 7.3 Hz, 2H), 1.44 (d, *J* = 7.2 Hz, 3H), 1.25 (s, 23H), 0.88 (t, *J* = 13.8, 6.5 Hz, 3H); **^{13}C NMR (101 MHz, $CDCl_3$)**: δ 174.64, 174.24, 172.90, 172.04, 158.84, 77.48, 77.16, 76.84, 71.23, 70.53, 70.49, 70.17, 70.00, 69.52, 64.39, 63.05, 53.44, 49.32, 35.12, 34.40, 32.07, 31.24, 29.85, 29.81, 29.78, 29.74, 29.63, 29.51, 29.44, 29.30, 25.04, 22.84, 16.95, 14.28, 0.14; **HRMS:** $[M+H]^+$ calculated for $C_{33}H_{63}N_4O_{10}S$ 707.4260, found 707.4279.

Cell culture

The HEK-hTLR2 reporter cell line are Hek293 cells stably transfected with human TLR2, and were obtained from InvivoGen. They were cultured at 37°C in a 5% CO_2 atmosphere in Iscove's Modified Dulbecco's Medium (IMDM, Gibco) containing 8% fetal calf serum (FCS, Bodinco B.V.), 2 mM L-glutamine (Gibco), 100 IU/ml penicillin and 100 µg/ml streptomycin (Pen Strep, Gibco), 25 µM β-mercaptoethanol (Gibco), 500ug/ml blasticidin.

The D1 dendritic cell line is a long-term growth factor-dependent, spleen-derived DC from a female C57BL/6 (H-2^b) mouse. D1 cells were cultured as previously described.[1] Bone marrow-derived dendritic cells (BMDCs) isolated from the bone marrow of C57BL/6 mice and cultured for 10 days as described elsewhere.[1] D1 dendritic cells and BMDCs were cultured at 37°C in a 5% CO_2 atmosphere in IMDM (Gibco) containing 8% FCS (Bodinco B.V.), 4 mM glutamax (Gibco), 100 IU/ml penicillin and 100 µg/ml streptomycin (Pen Strep, Gibco), 50 µM β-mercaptoethanol (Gibco), and supplemented with GM-CSF supernatant.[1]

Monocyte-derived dendritic cells (moDCs) were obtained from peripheral blood mononuclear cells (PBMCs) and cultured as previously described.[2] Briefly, buffy coats from healthy donors (Sanquin Blood Bank) were centrifuged over a ficoll gradient Leucosep tube (Bio Greiner) to obtain peripheral blood mononuclear cells (PBMCs). $CD14^+$ monocytes were isolated by using magnetic $CD14^+$ microbeads (Miltenyi Biotec) on the PBMCs. The $CD14^+$ monocytes were cultured in IMDM (Gibco) supplemented with 8% FCS (Bodinco B.V.), 2 mM L-glutamine (Gibco), 100 IU/ml penicillin and 100 µg/ml streptomycin (Pen Strep, Gibco), 25 µM β-mercaptoethanol (Gibco), 500 U/ml of human IL-4 and 800 U/ml human GM-CSF (Mitenyi Biotec) at 37°C in a 5% CO_2 atmosphere. After 3 days, fresh medium was added with 1000 U/ml human IL-4 and 1600 human GM-CSF. On day 5, resulting moDCs were used for experiments.

HEK-Blue hTLR2 hTLR1-KO hTLR6-KO cells are reporter cell lines bought from InvivoGen; they are generated by the double knockout (KO) of the endogenous hTLR1 and hTLR6 genes in the HEK-Blue hTLR2 cell line, a human embryonic kidney (HEK) 293-derived cell line that overexpresses the human TLR2, CD14, and NF-κB-inducible SEAP (secreted embryonic alkaline phosphatase) reporter gene. HEK-Blue hTLR2-hTLR1, generated by stable transfection of the human TLR1 gene into the HEK-Blue hTLR2 hTLR1-KO TLR6-KO cell line, and HEK-Blue hTLR2-hTLR6 cells, generated by stable transfection of the human TLR6 gene into the HEK-Blue hTLR2 hTLR1-KO hTLR6-KO cell line was bought from InvivoGen. HEK-Blue hTLR2 hTLR1-KO hTLR6-KO, HEK-Blue hTLR2-hTLR1, and HEK-Blue hTLR2-hTLR6 cells were cultured at 37°C in a 5% CO_2 atmosphere in IMDM (Gibco) supplemented with 8% FCS (Bodinco B.V.), 2 mM L-glutamine (Gibco), 100 IU/ml penicillin, 100 µg/ml streptomycin (Pen Strep, Gibco), 100 µg/ml normocin (InvivoGen), 25 µM β-mercaptoethanol (Gibco), and 1X Hek-Blue Selection (InvivoGen).

Activation human TLR2 transfected HEK cells

50,000 Hek-hTLR2 were added to 96-well round-bottom plates (Greiner) containing titrated test compounds in complete IMDM medium. After overnight incubation at 37°C in a 5% CO_2 atmosphere, the supernatant was harvested to measure the amount of produced human IL-8 by a sandwich ELISA (BioLegend).

Dendritic cell maturation determination

The dendritic cells (murine D1s, murine BMDCs, or human moDCs) were seeded in a 96-well round-bottom plate (Greiner), 50,000 cells per well, in complete IMDM medium, with titrated amounts of the test compounds. LPS at 2µg/ml was used as a positive control. After overnight incubation at 37°C and 5% CO_2, the supernatant was harvested to measure the amount of produced murine (for D1 dendritic cell line and bone marrow-derived dendritic cells) or human (for monocyte-derived dendritic cells) IL-12p40 by a sandwich ELISA (BioLegend).

Specificity of hTLR2/1 and hTLR2/6 heterodimers

The cell lines were seeded in a 96-well flat-bottom plate (Greiner), 50,000 cells per well, in Hek-Blue Detection medium (InvivoGen). Titrated test compounds, in PBS, were added to the plate. After 24 hours of incubation at 37°C and 5% CO_2, TLR signaling activation was detected by measuring the absorbance at 655 nm with a microplate reader (Tecan Infinite 200 Pro M Plex Microplate Reader).

Statistical analysis

Graphical representation and statistics were performed using GraphPad Prism 8.02 (San Diego, USA). A 2-way ANOVA with Tukey's multiple comparisons was performed for all figures unless specified in the legend. Significance was determined with p-values below 0.05. *p<0.05; **p<0.01; ***p<0.001

Bibliography

1. Winzler, C. *et al.* Maturation stages of mouse dendritic cells in growth factor-dependent long-term cultures. *J. Exp. Med.* **185**, 317–328 (1997).

2. Ende, T. C. *et al.* Simplified Monopalmitoyl Toll-like Receptor 2 Ligand Mini-UPam for Self-Adjuvanting Neoantigen-Based Synthetic Cancer Vaccines. *ChemBioChem* **22**, 1215–1222 (2020).

NMR spectra for synthetic ligands

Compound **1**

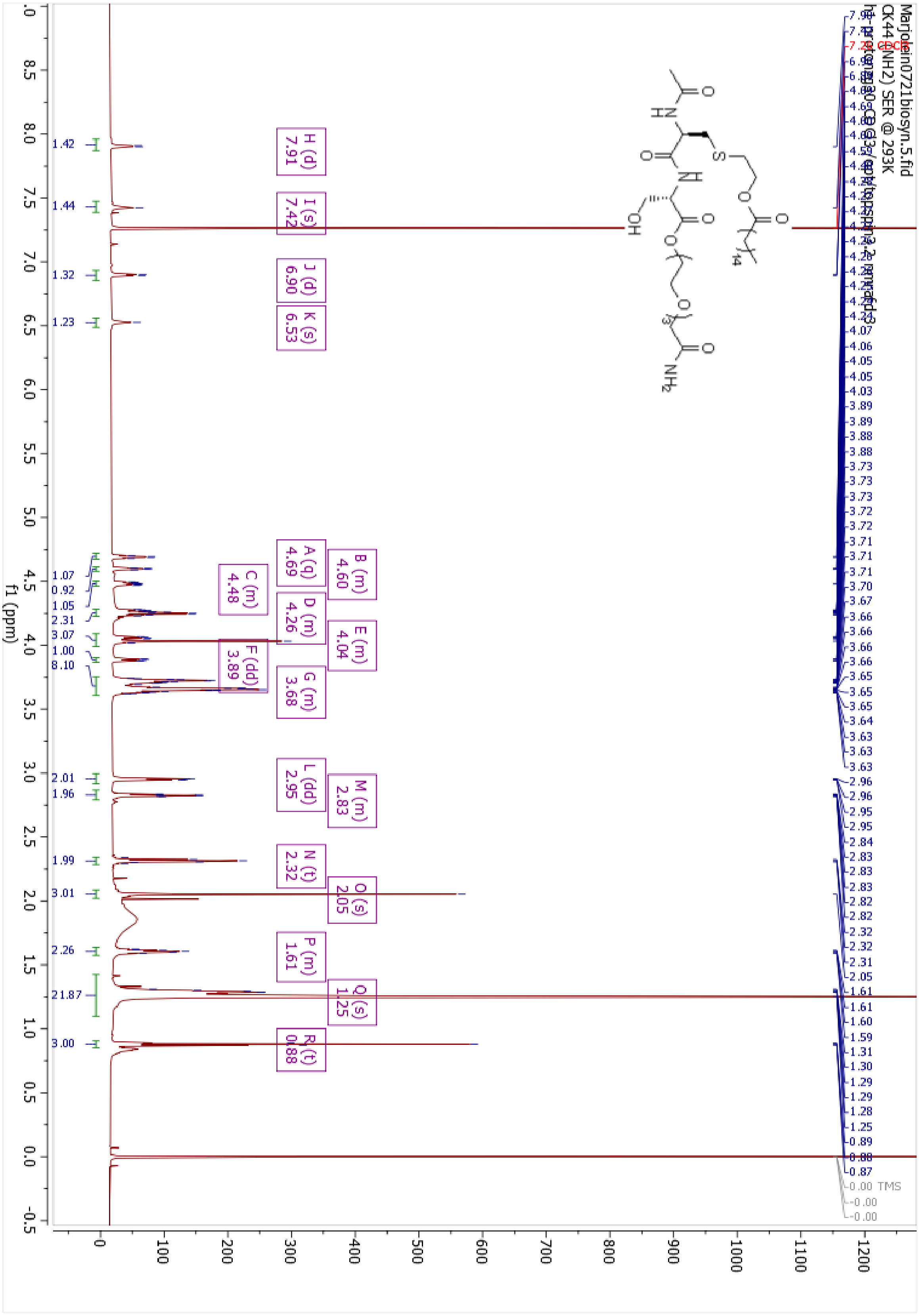

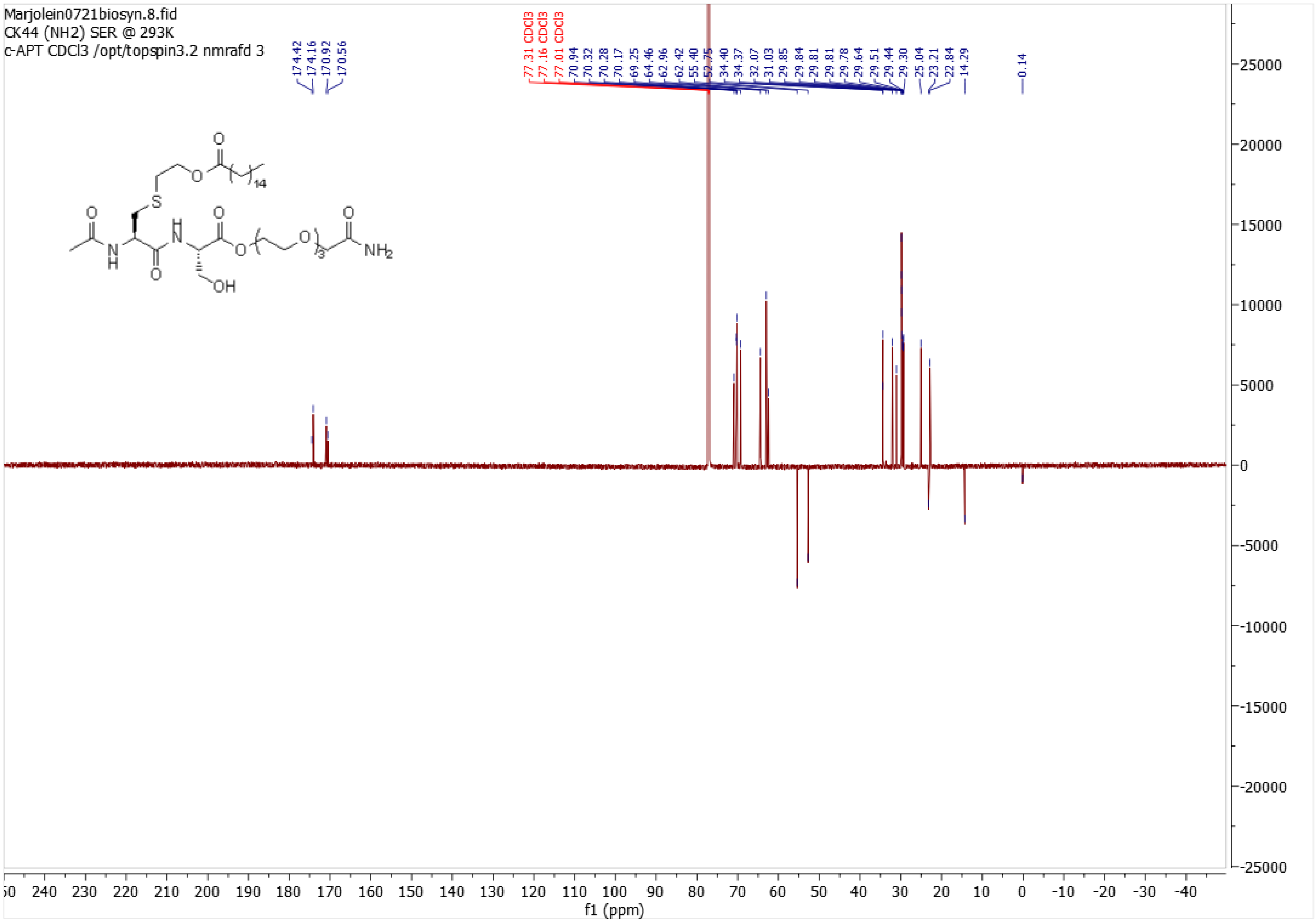
Marjolein0721biosyn.8.fid
CK44 (NH2) SER @ 293K
c-APT CDCl3 /opt/topspin3.2 nmrafd 3
174.42
174.16
170.92
170.56
77.31 CDCl3
77.16 CDCl3
77.01 CDCl3
70.94
70.32
70.28
70.17
69.25
64.46
62.96
62.42
55.40
52.75
34.40
34.37
32.07
31.03
29.85
29.84
29.81
29.81
29.78
29.64
29.51
29.44
29.30
25.04
23.21
22.84
14.29
0.14
f1 (ppm)

Compound **2**

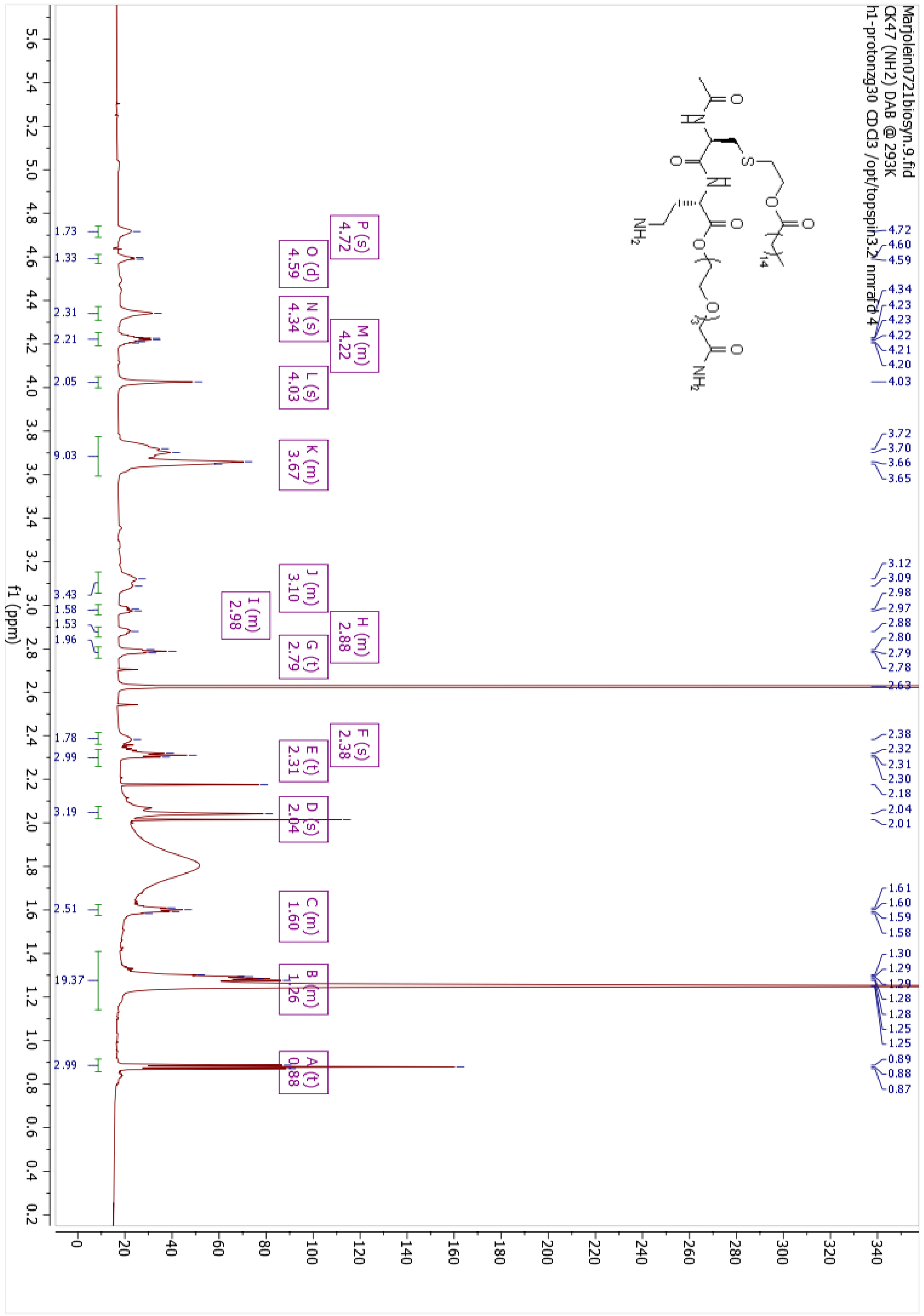

Marjolein0721biosyn.9.fid
CK47 (NH2) DAB @ 293K
h1-protonzg30 CDCl3 /opt/topspin3.2 nmrafd 4
f1 (ppm)

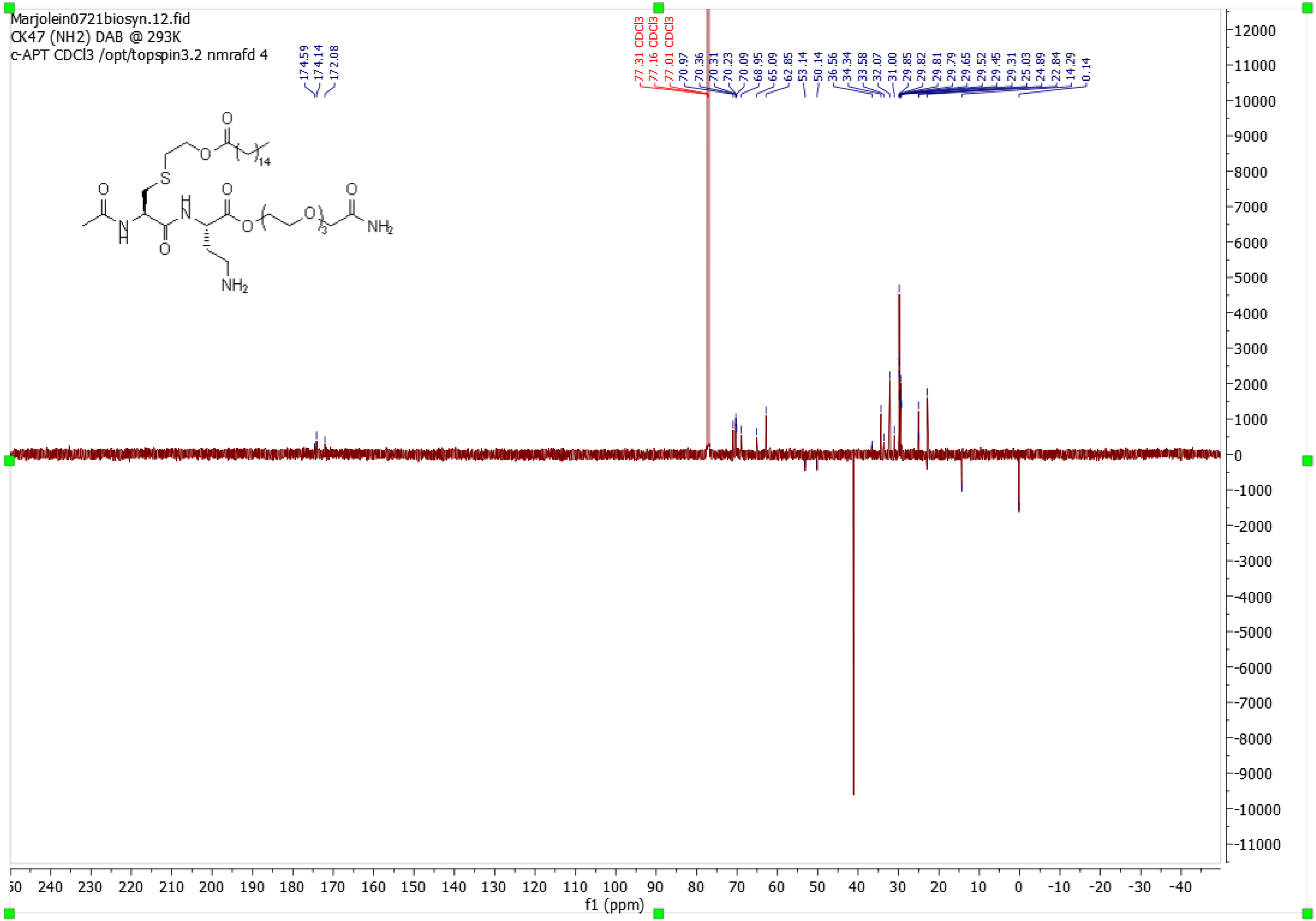
Marjolein0721biosyn.12.fid
CK47 (NH2) DAB @ 293K
c-APT CDCl3 /opt/topspin3.2 nmrafd 4
174.59
174.14
172.08
77.31 CDCl3
77.16 CDCl3
77.01 CDCl3
70.97
70.36
70.31
70.23
70.09
68.95
65.09
62.85
53.14
50.14
36.56
34.34
33.58
32.07
31.00
29.85
29.82
29.81
29.79
29.65
29.52
29.45
29.31
25.03
24.89
22.84
14.29
0.14
f1 (ppm)

Compound **3**

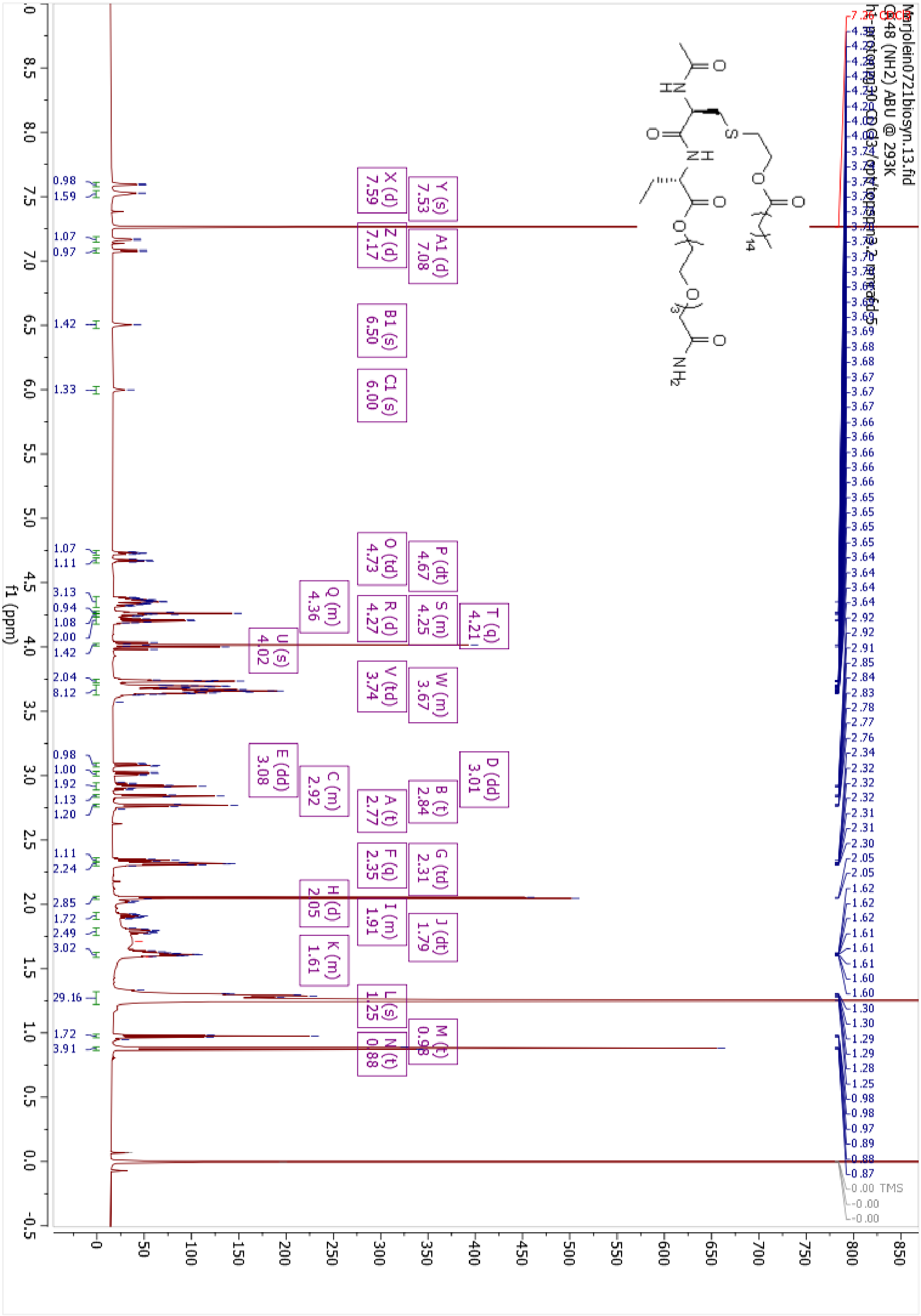
Marjolein0721biosyn.13.fid
C48 (NH2) ABU @ 293K
f1 (ppm)
Y (s) 7.53
X (d) 7.59
A1 (d) 7.08
Z (d) 7.17
B1 (s) 6.50
C1 (s) 6.00
P (dt) 4.67
O (td) 4.73
Q (m) 4.36
S (m) 4.25
R (d) 4.27
T (q) 4.21
U (s) 4.02
W (m) 3.67
V (td) 3.74
D (dd) 3.01
E (dd) 3.08
C (m) 2.92
B (t) 2.84
A (t) 2.77
G (td) 2.31
F (q) 2.35
H (d) 2.05
I (m) 1.91
J (dt) 1.79
K (m) 1.61
L (s) 1.25
M (t) 0.98
N (t) 0.88
TMS

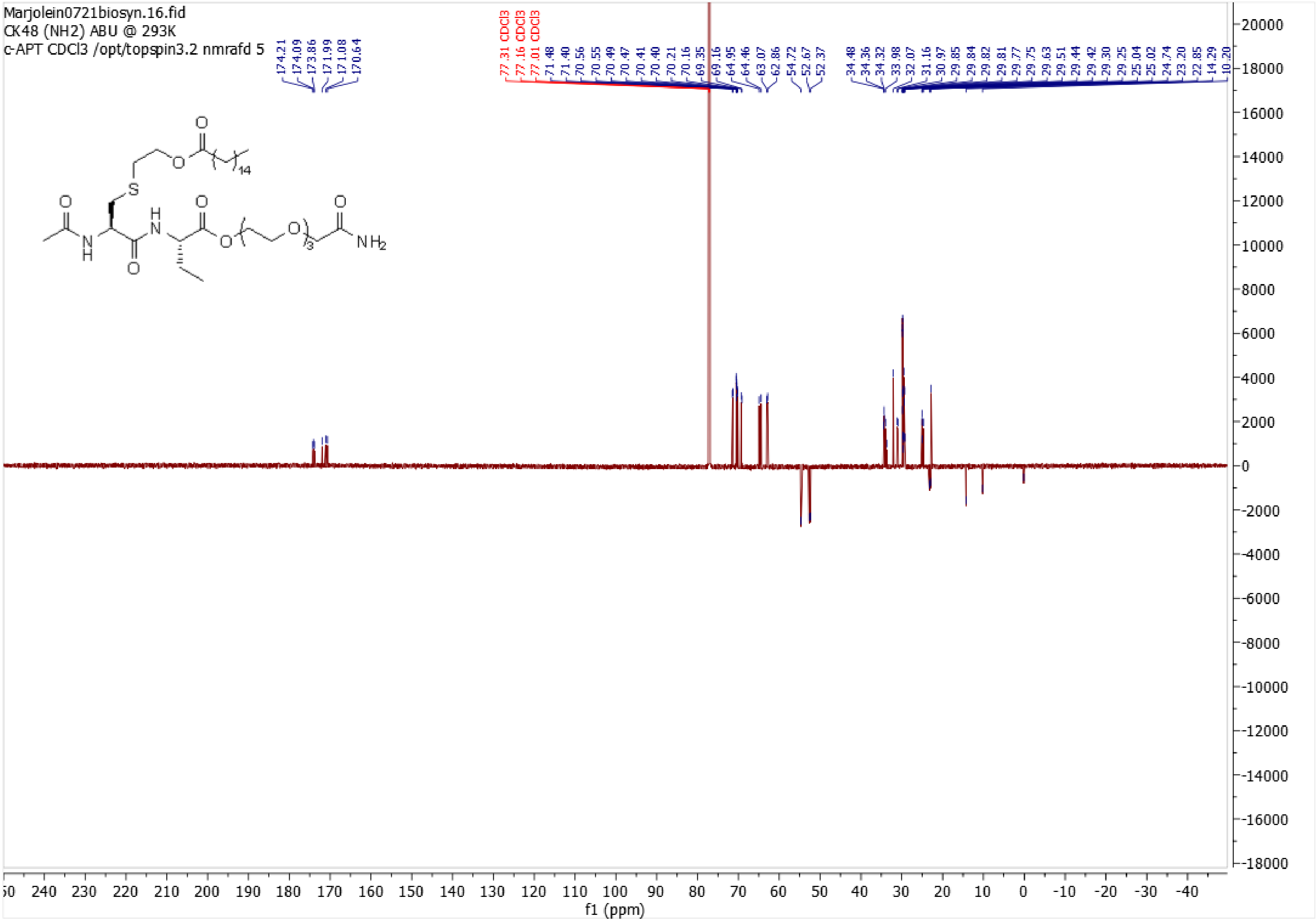
Marjolein0721biosyn.16.fid
CK48 (NH2) ABU @ 293K
c-APT CDCl3 /opt/topspin3.2 nmrafd 5
174.21
174.09
173.86
171.99
171.08
170.64
77.31 CDCl3
77.16 CDCl3
77.01 CDCl3
71.48
71.40
70.56
70.55
70.49
70.47
70.41
70.40
70.21
70.16
69.35
69.16
64.95
64.46
63.07
62.86
54.72
52.67
52.37
34.48
34.36
34.32
33.98
32.07
31.16
30.97
29.85
29.84
29.82
29.81
29.77
29.75
29.63
29.51
29.44
29.42
29.30
29.25
25.04
25.02
24.74
23.20
22.85
14.29
10.20
f1 (ppm)

Compound **4**

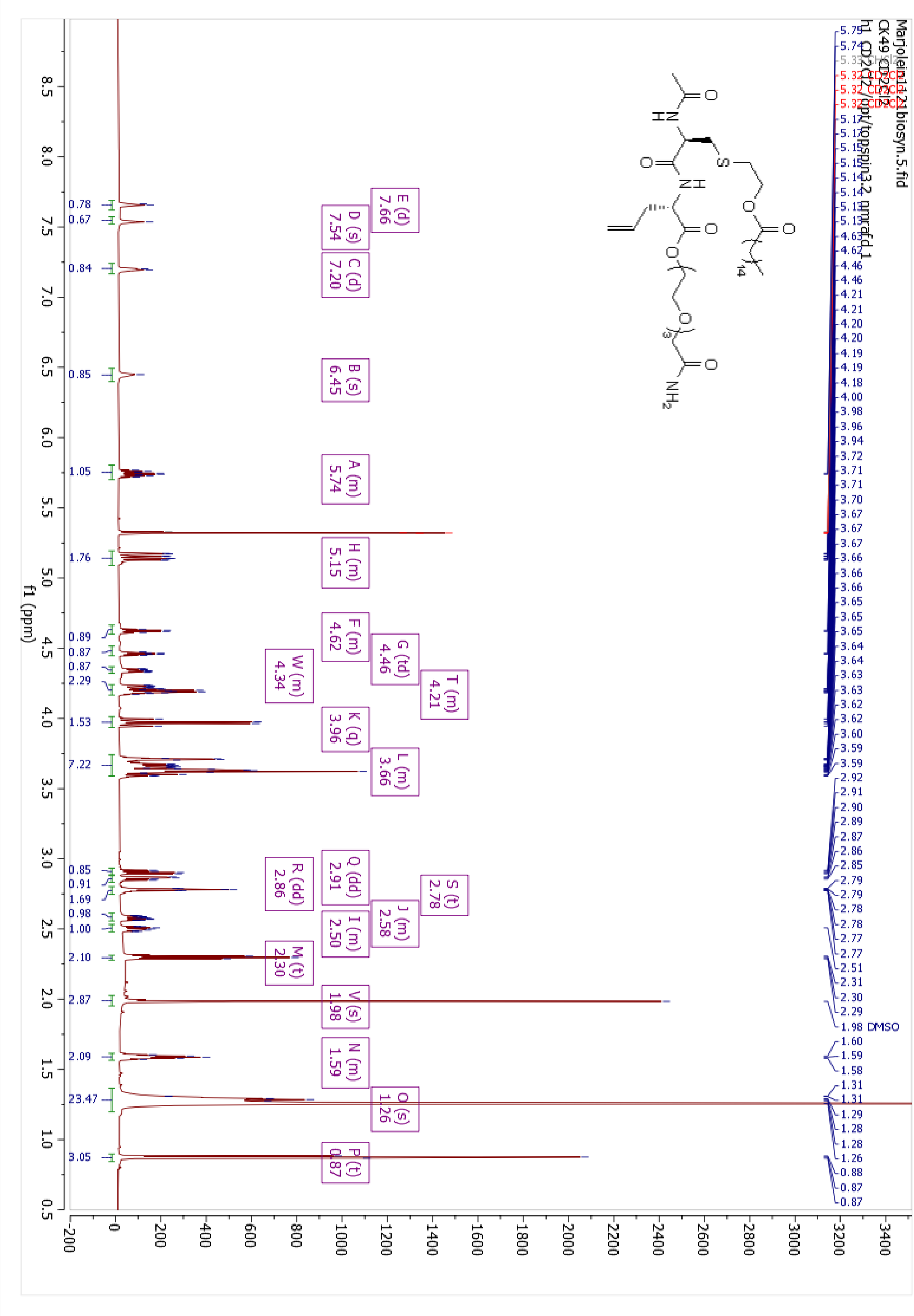

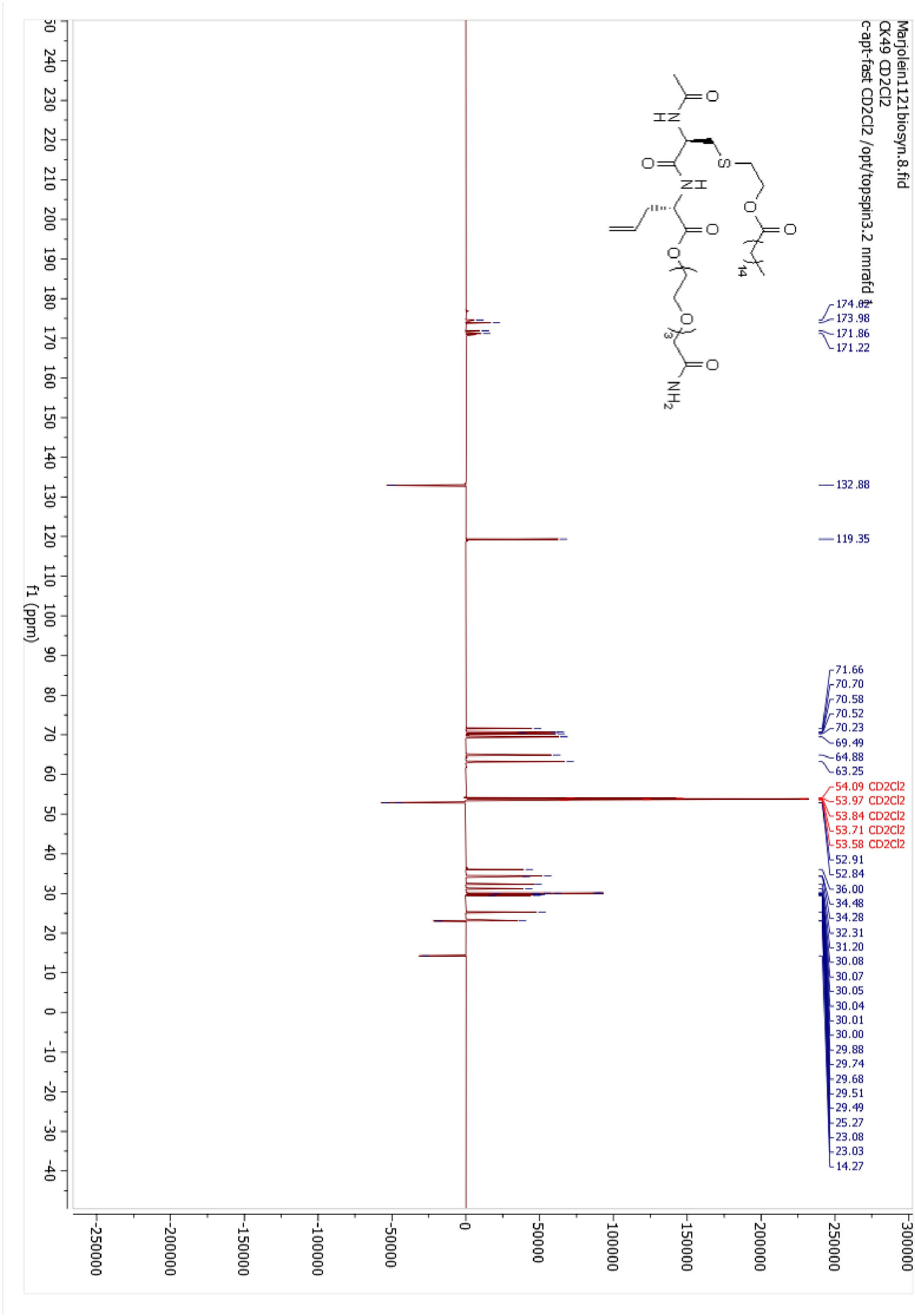
Marjolein1121biosyn.8.fid
CK49 CD2Cl2
c-apt-fast CD2Cl2 /opt/topspin3.2 nmrafd
174.02
173.98
171.86
171.22
132.88
119.35
71.66
70.70
70.58
70.52
70.23
69.49
64.88
63.25
54.09 CD2Cl2
53.97 CD2Cl2
53.84 CD2Cl2
53.71 CD2Cl2
53.58 CD2Cl2
52.91
52.84
36.00
34.48
34.28
32.31
31.20
30.08
30.07
30.05
30.04
30.01
30.00
29.88
29.74
29.68
29.51
29.49
25.27
23.08
23.03
14.27
f1 (ppm)

Compound **5**

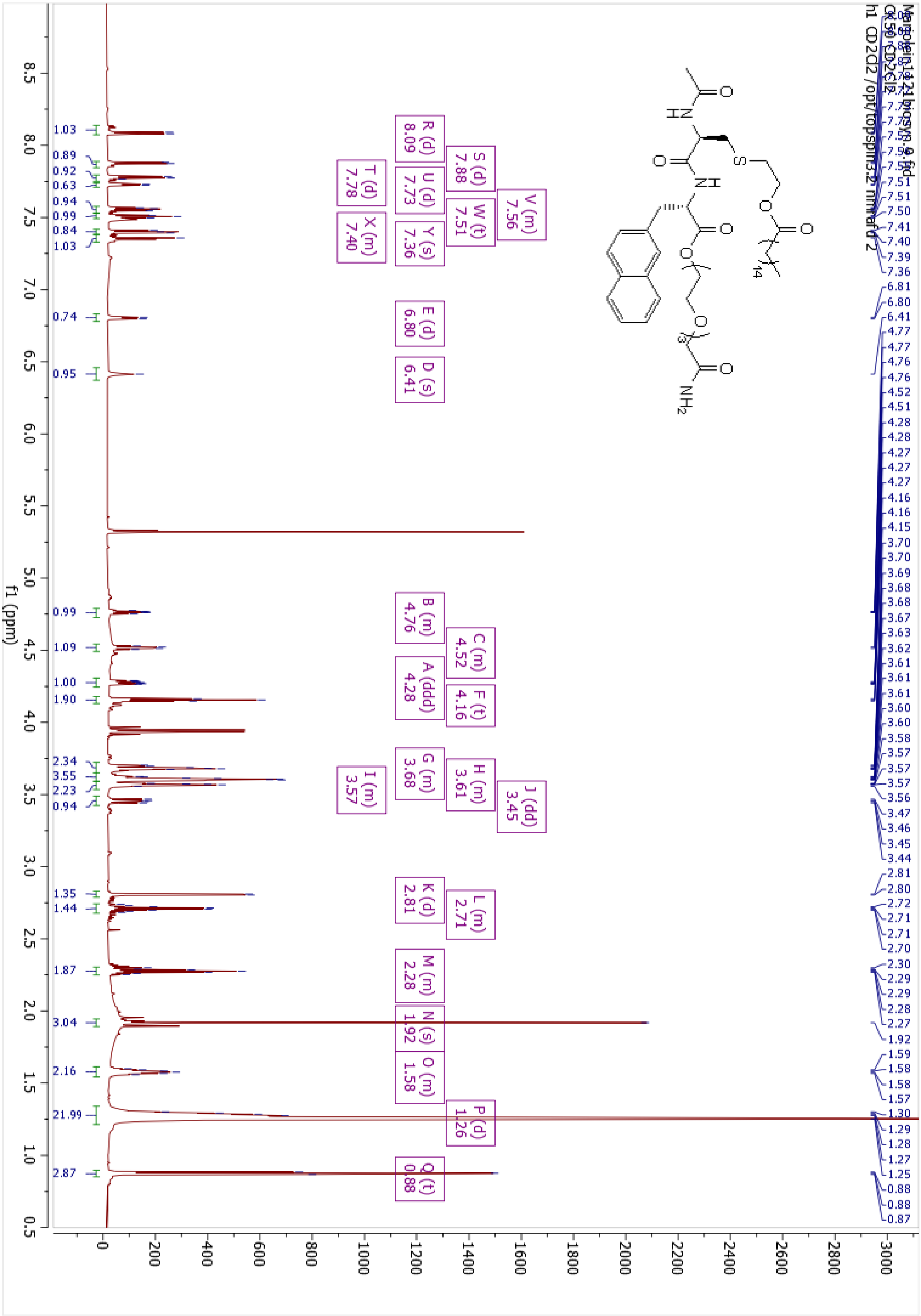

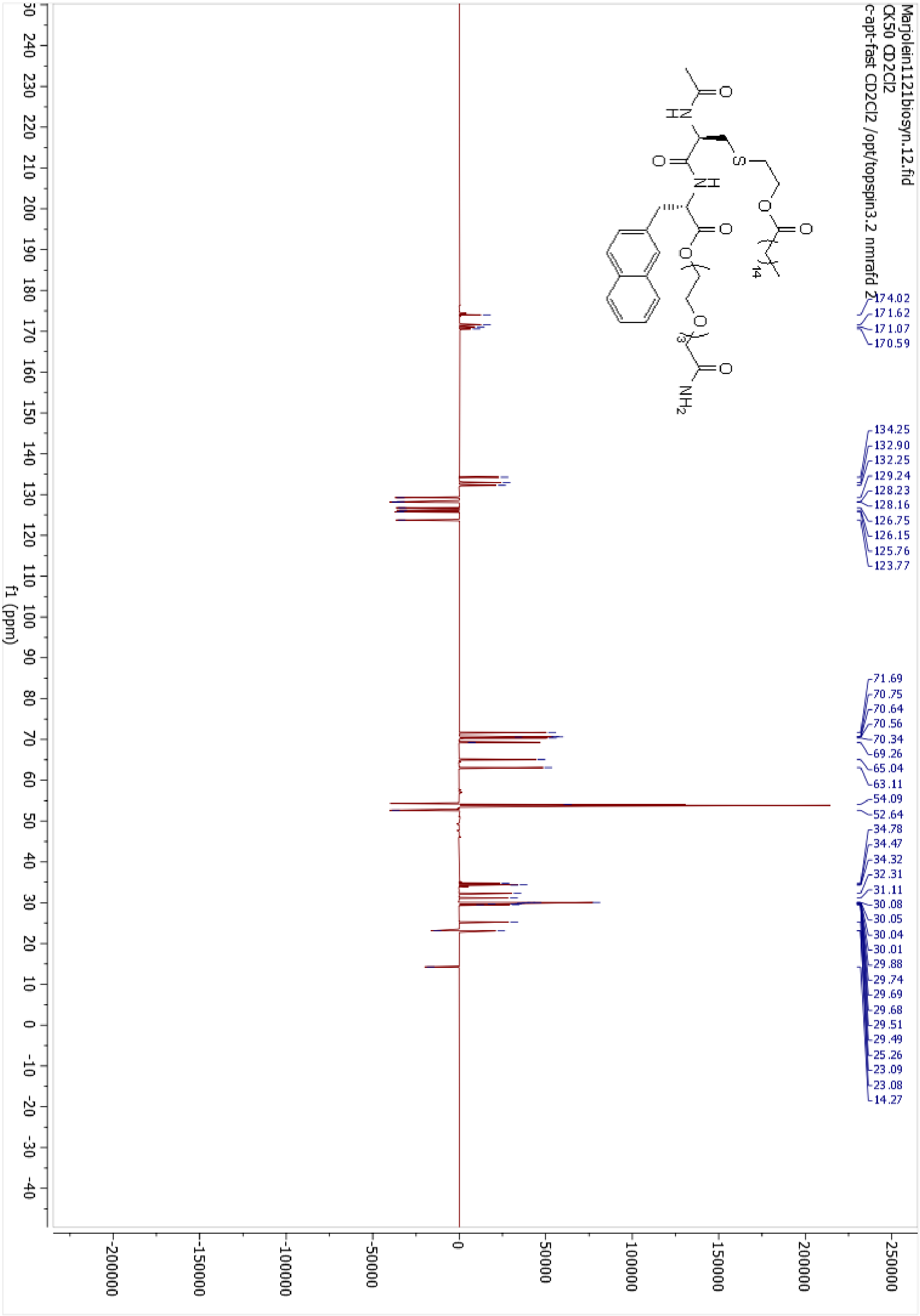
Marjolein1121biosyn.12.fid
CK50 CD2Cl2
c-apt-fast CD2Cl2 /opt/topspin3.2 nmrafd 2
174.02
171.62
171.07
170.59
134.25
132.90
132.25
129.24
128.23
128.16
126.75
126.15
125.76
123.77
71.69
70.75
70.64
70.56
70.34
69.26
65.04
63.11
54.09
52.64
34.78
34.47
34.32
32.31
31.11
30.08
30.05
30.04
30.01
29.88
29.74
29.69
29.68
29.51
29.49
25.26
23.09
23.08
14.27
f1 (ppm)

Compound **6**

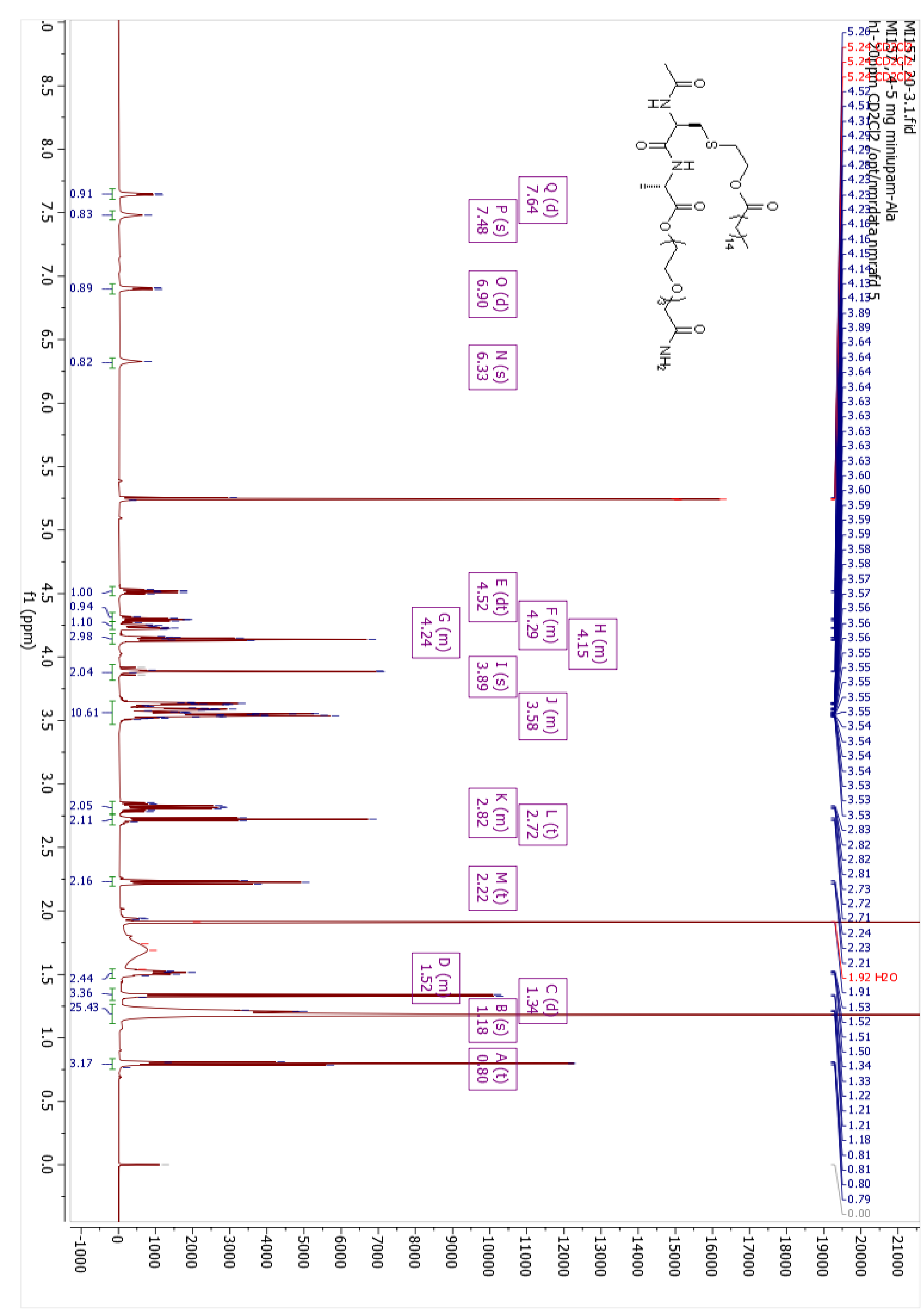

Q (d) 7.64
P (s) 7.48
O (d) 6.90
N (s) 6.33
E (dt) 4.52
F (m) 4.29
H (m) 4.15
G (m) 4.24
I (s) 3.89
J (m) 3.58
K (m) 2.82
L (t) 2.72
M (t) 2.22
D (m) 1.52
C (d) 1.34
B (s) 1.18
A (t) 0.80
f1 (ppm)

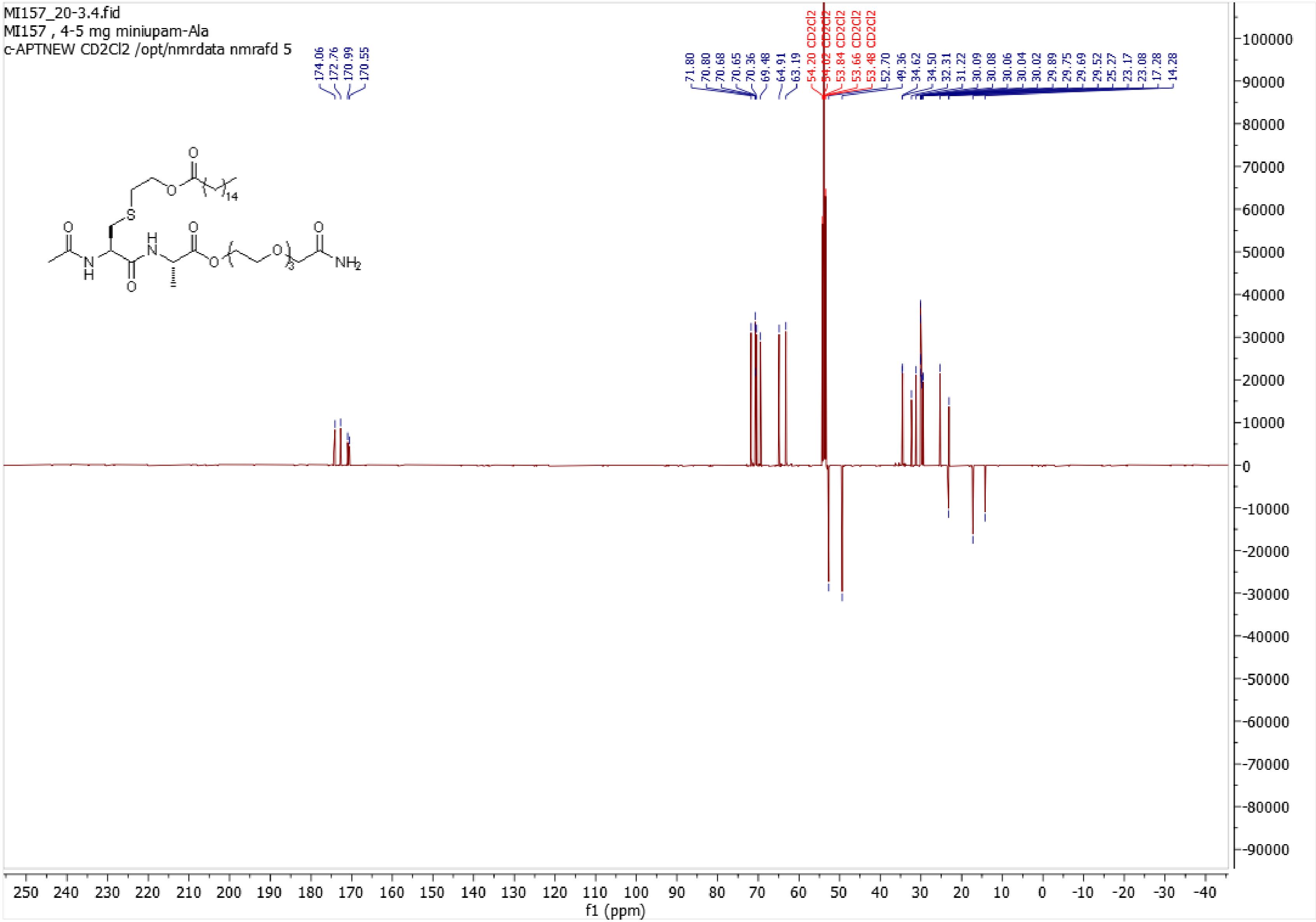
MI157_20-3.4.fid
MI157 , 4-5 mg miniupam-Ala
c-APTNEW CD2Cl2 /opt/nmrdata nmrafd 5
174.06
172.76
170.99
170.55
71.80
70.80
70.68
70.65
70.36
69.48
64.91
63.19
54.20 CD2Cl2
54.02 CD2Cl2
53.84 CD2Cl2
53.66 CD2Cl2
53.48 CD2Cl2
52.70
49.36
34.62
34.50
32.31
31.22
30.09
30.08
30.06
30.04
30.02
29.89
29.75
29.69
29.52
25.27
23.17
23.08
17.28
14.28
f1 (ppm)

Compound **7**

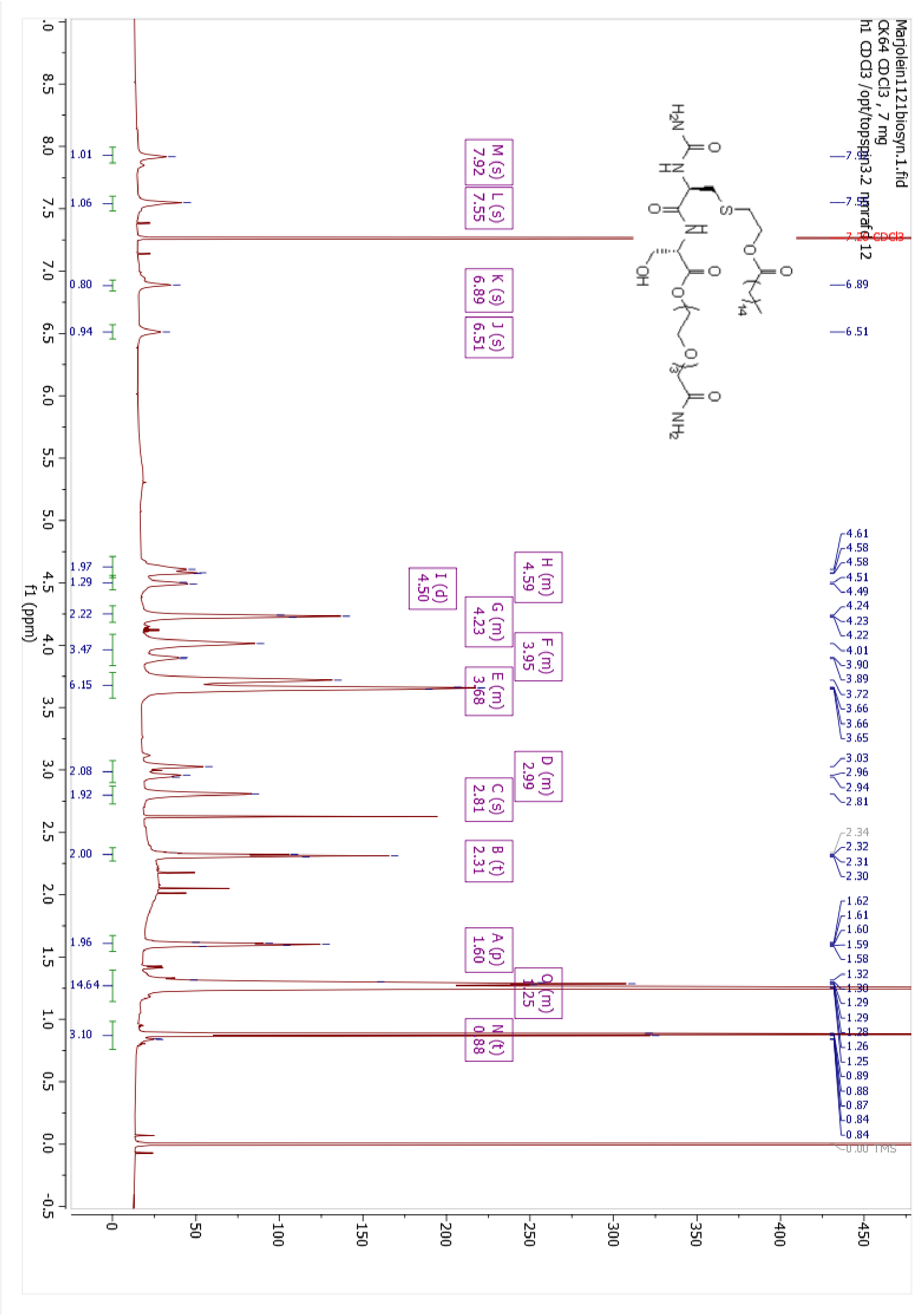

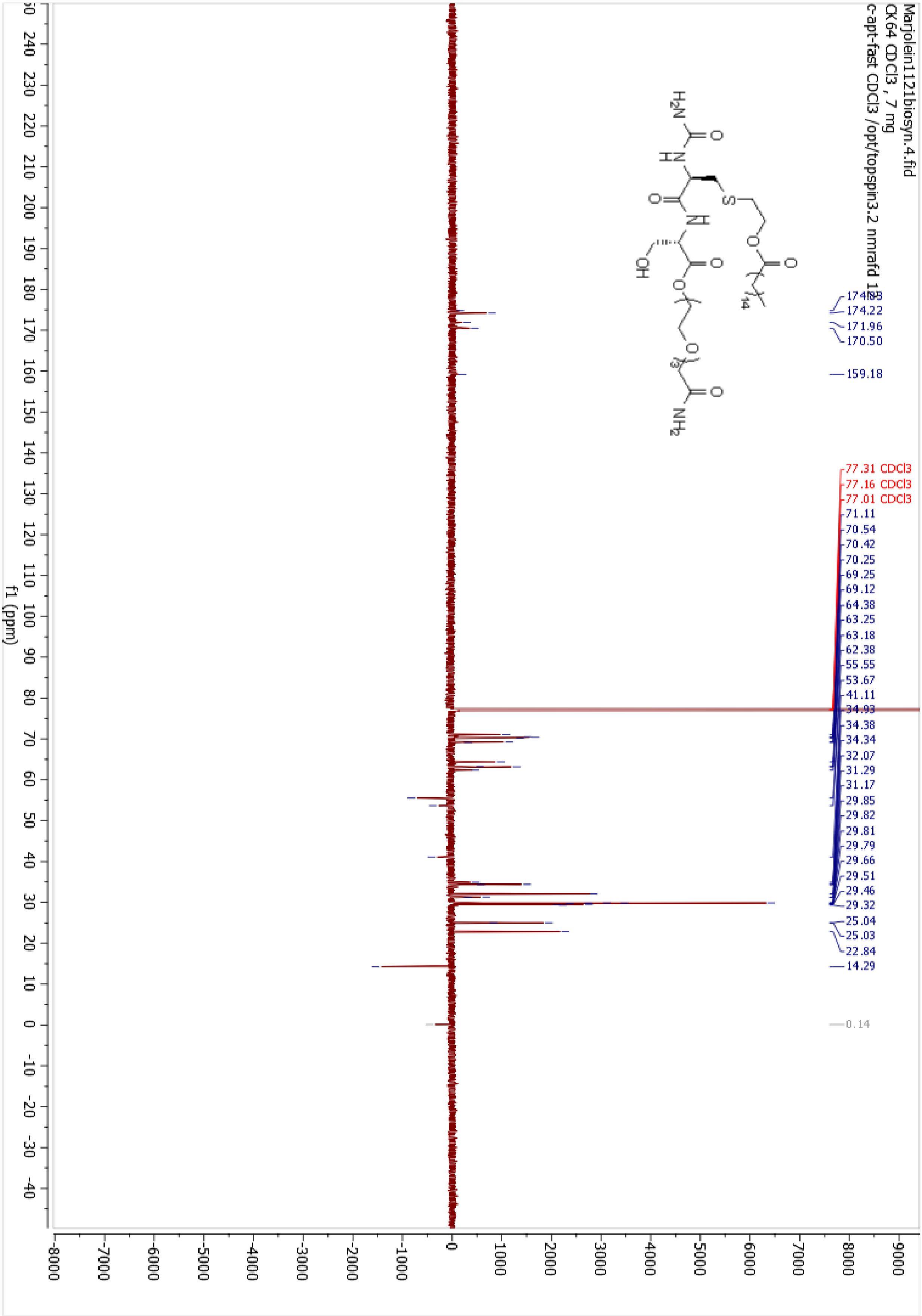
Marjolein1121biosyn.4.fid
CK64 CDCl3 , 7 mg
c-apt-fast CDCl3 /opt/topspin3.2 nmrafd
174.22
171.96
170.50
159.18
77.31 CDCl3
77.16 CDCl3
77.01 CDCl3
71.11
70.54
70.42
70.25
69.25
69.12
64.38
63.25
63.18
62.38
55.55
53.67
41.11
34.93
34.38
34.34
32.07
31.29
31.17
29.85
29.82
29.81
29.79
29.66
29.51
29.46
29.32
25.04
25.03
22.84
14.29
0.14
f1 (ppm)

Compound **8**

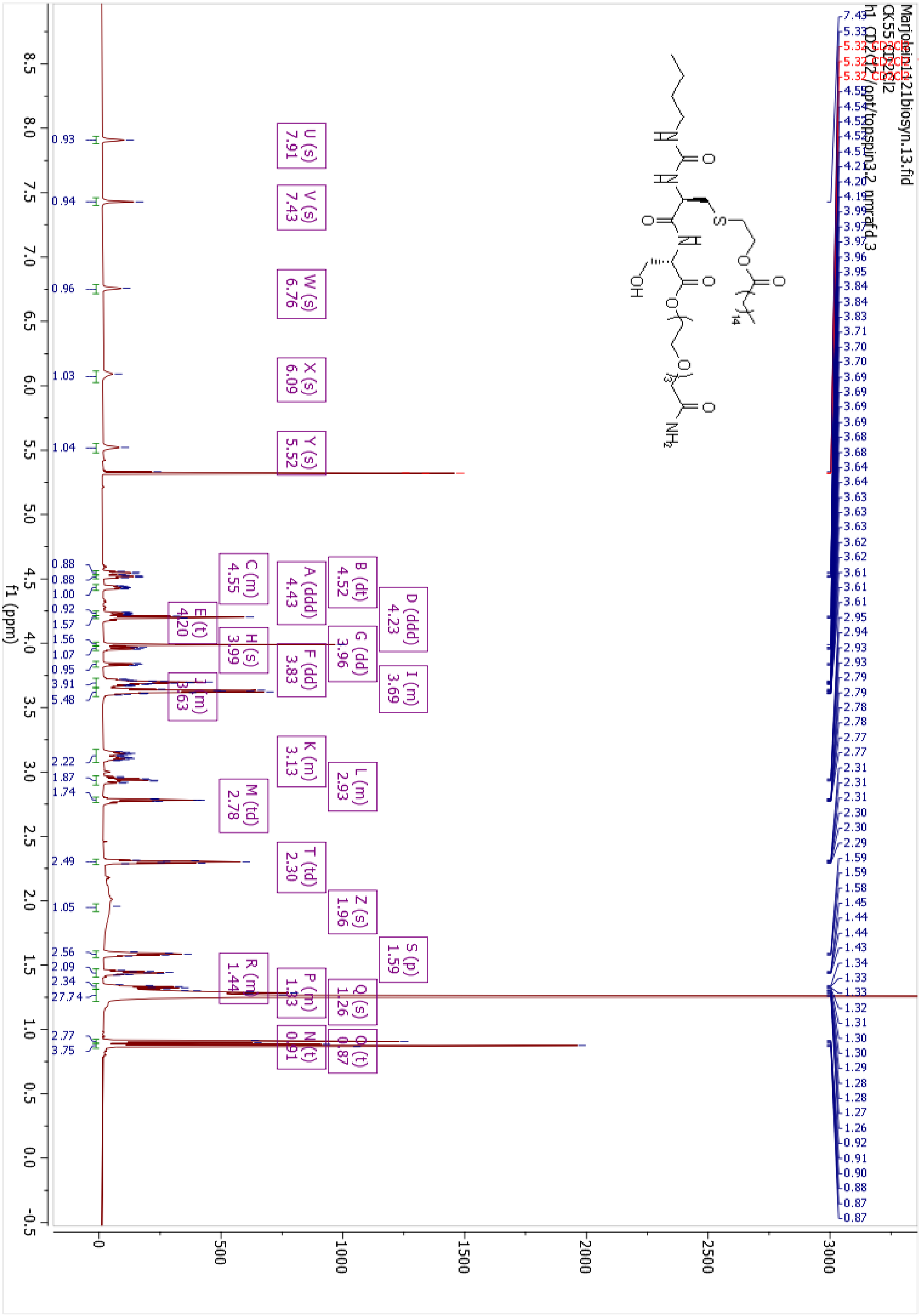

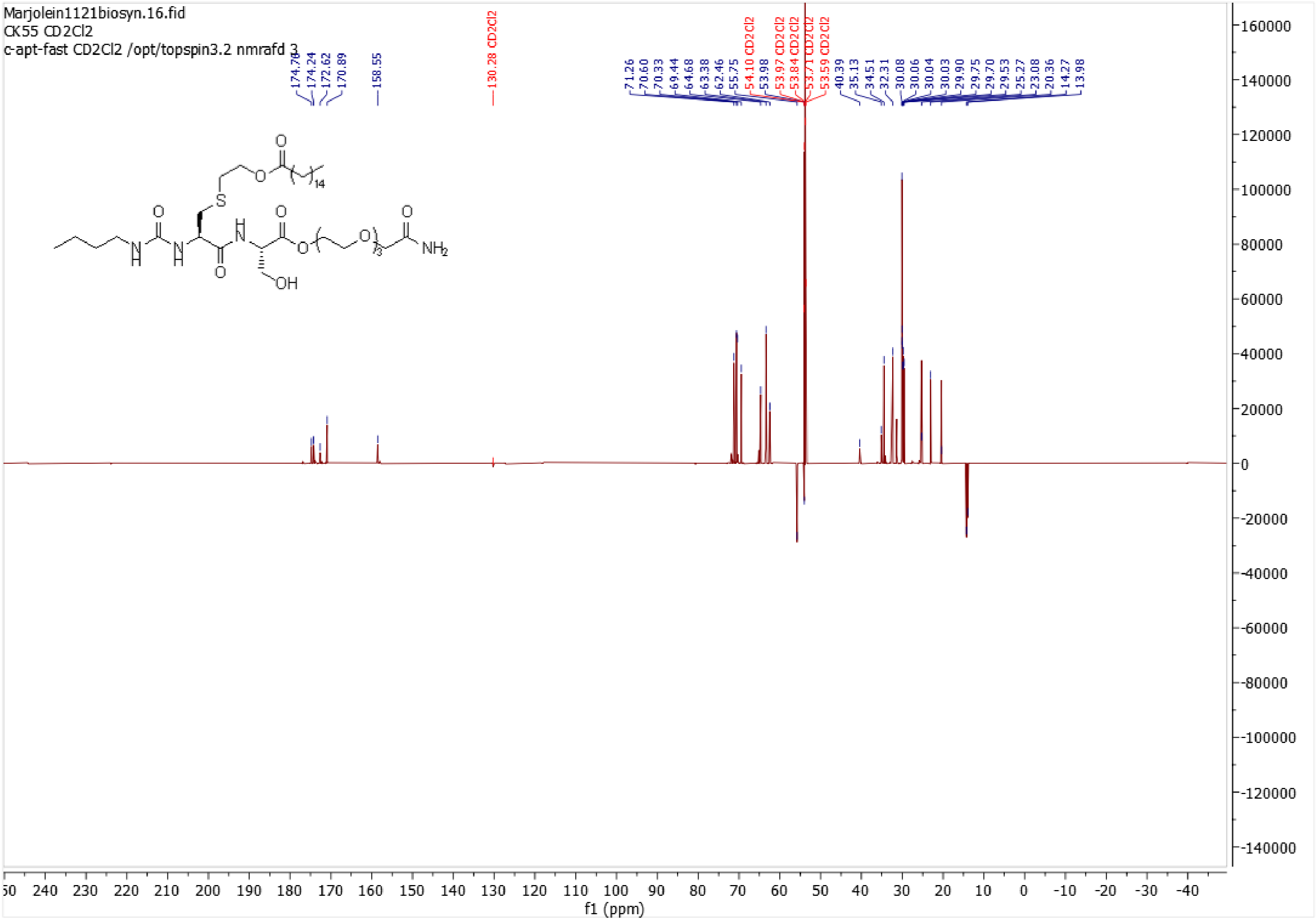
Marjolein1121biosyn.16.fid
CK55 CD2Cl2
c-apt-fast CD2Cl2 /opt/topspin3.2 nmrafd 3
174.70
174.24
172.62
170.89
158.55
130.28 CD2Cl2
71.26
70.60
70.33
69.44
64.68
63.38
62.46
55.75
54.10 CD2Cl2
53.98
53.97 CD2Cl2
53.84 CD2Cl2
53.71 CD2Cl2
53.59 CD2Cl2
40.39
35.13
34.51
32.31
30.08
30.06
30.04
30.03
29.90
29.75
29.70
29.53
25.27
23.08
20.36
14.27
13.98
O
S
O
14
O
N
H
N
H
O
H
N
O
O
O
3
O
NH2
OH
f1 (ppm)

Compound **9**

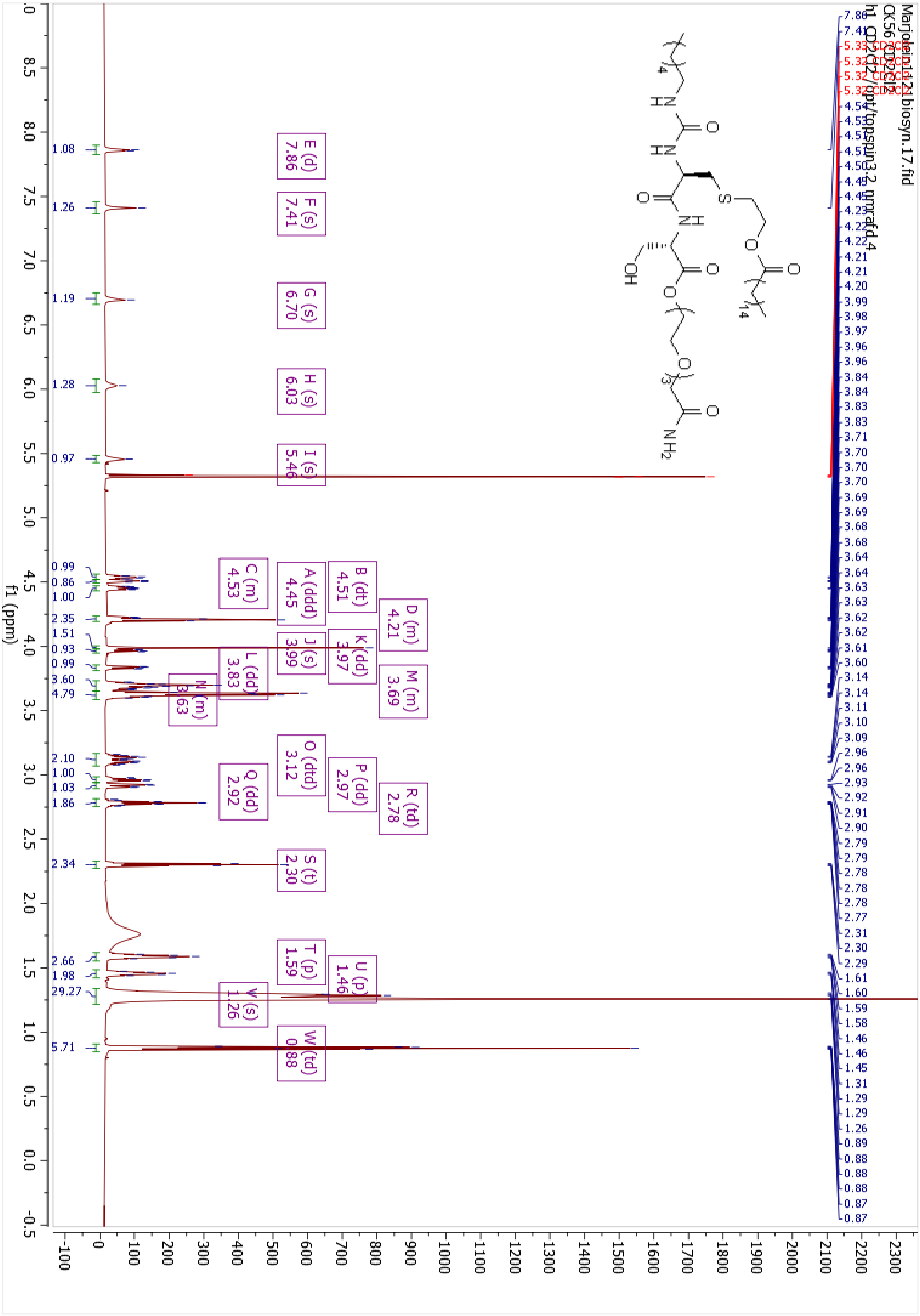

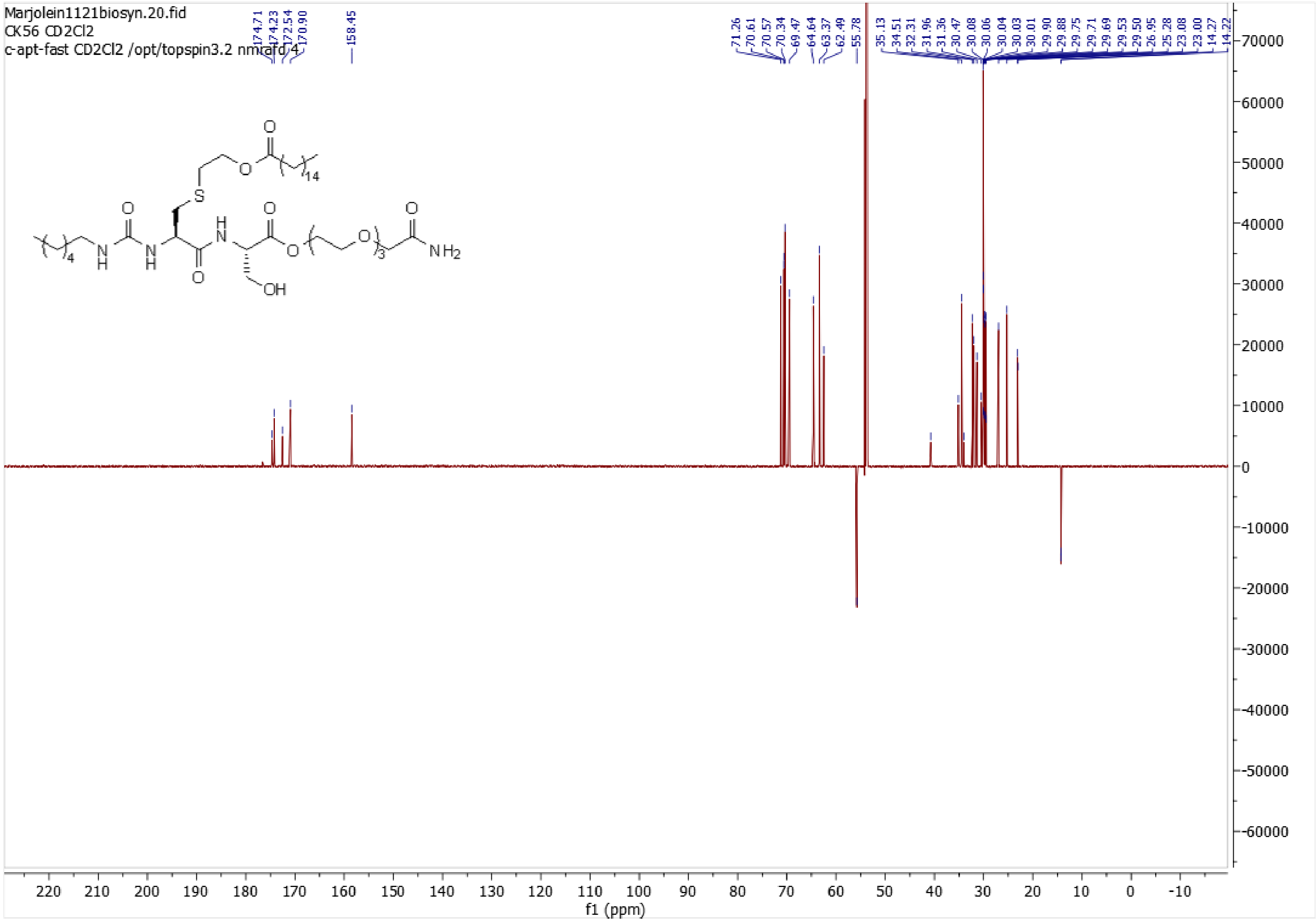
Marjolein1121biosyn.20.fid
CK56 CD2Cl2
c-apt-fast CD2Cl2 /opt/topspin3.2 nmrafd 4
174.71
174.23
172.54
170.90
158.45
71.26
70.61
70.57
70.34
69.47
64.64
63.37
62.49
55.78
35.13
34.51
32.31
31.96
31.36
30.47
30.08
30.06
30.04
30.03
30.01
29.90
29.88
29.75
29.71
29.69
29.53
29.50
26.95
25.28
23.08
23.00
14.27
14.22
f1 (ppm)

Compound **10**

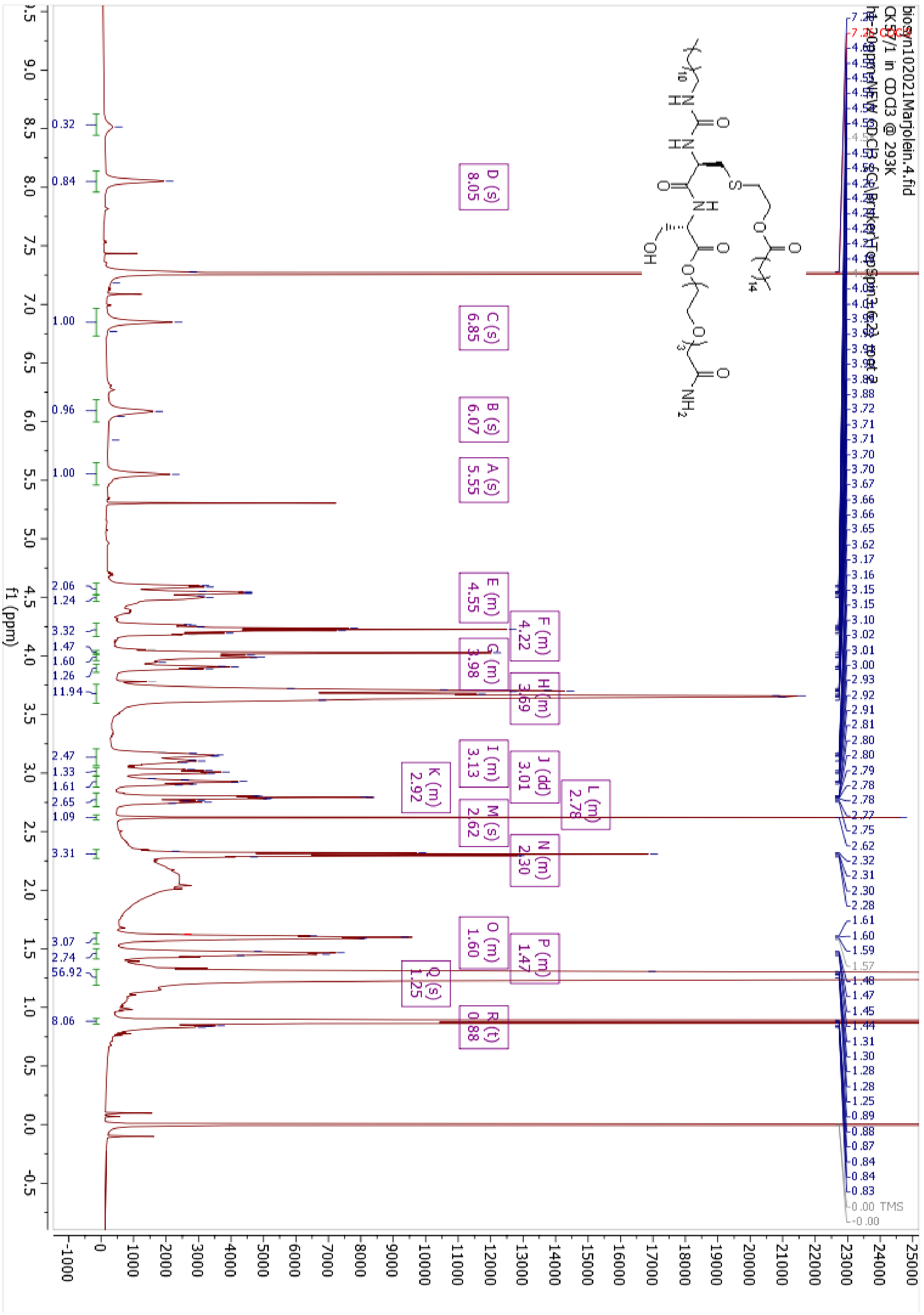

bioSyn102021Marjolein.4.fid
CK5/1 in CDCl3 @ 293K
D (s) 8.05
C (s) 6.85
B (s) 6.07
A (s) 5.55
E (m) 4.55
F (m) 4.22
G (m) 3.98
H (m) 3.69
I (m) 3.13
J (dd) 3.01
K (m) 2.92
L (m) 2.78
M (s) 2.62
N (m) 2.30
O (m) 1.60
P (m) 1.47
Q (s) 1.25
R (t) 0.88
f1 (ppm)

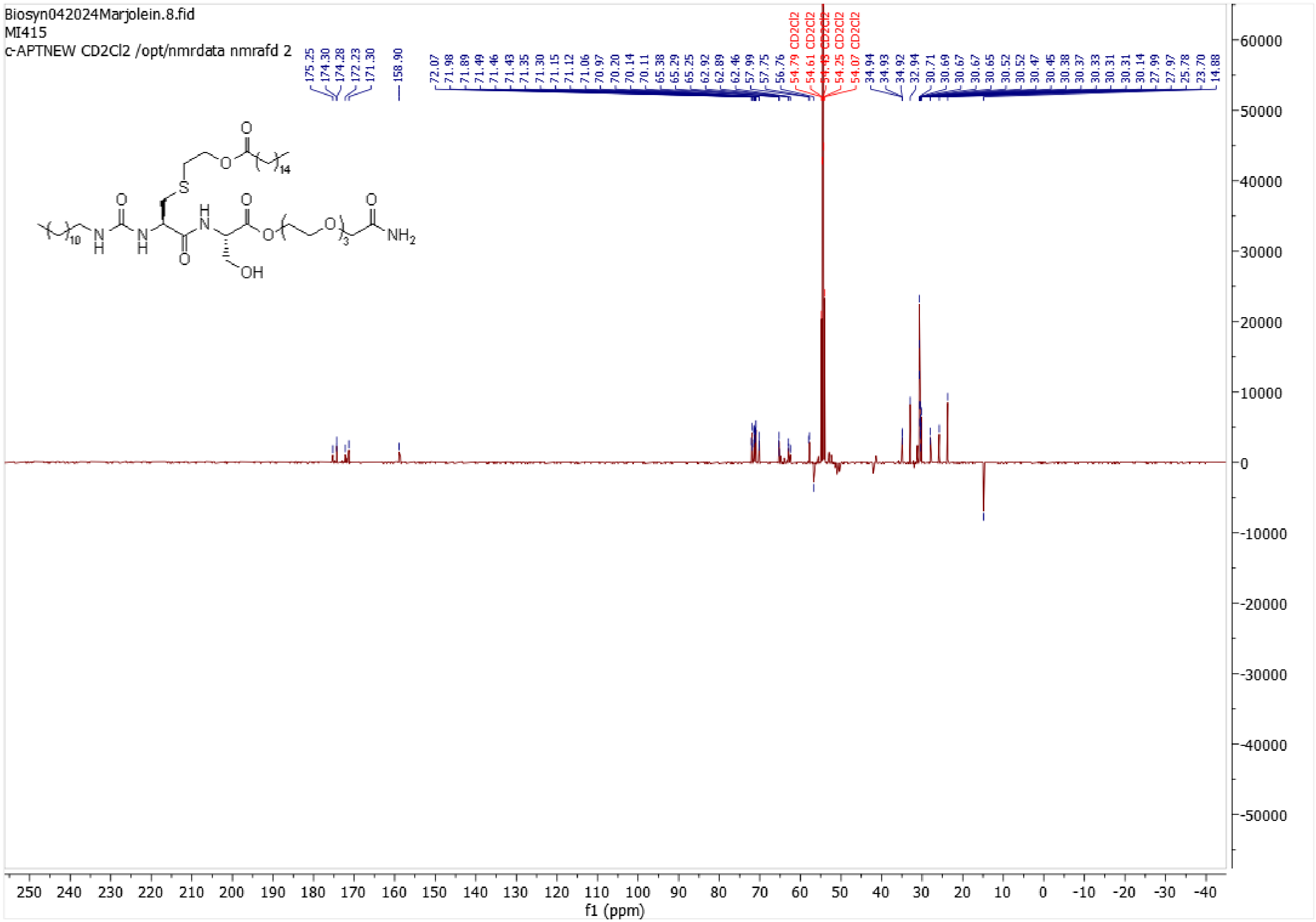

Biosyn042024Marjolein.8.fid
MI415
c-APTNEW CD2Cl2 /opt/nmrdata nmrafd 2
175.25
174.30
174.28
172.23
171.30
158.90
72.07
71.98
71.89
71.49
71.46
71.43
71.35
71.30
71.15
71.12
71.06
70.97
70.20
70.14
70.11
65.38
65.29
65.25
62.92
62.89
62.46
57.99
57.75
56.76
54.79 CD2Cl2
54.61 CD2Cl2
54.43 CD2Cl2
54.25 CD2Cl2
54.07 CD2Cl2
34.94
34.93
34.92
32.94
30.71
30.69
30.67
30.67
30.65
30.52
30.52
30.47
30.45
30.38
30.37
30.33
30.31
30.31
30.14
27.99
27.97
25.78
23.70
14.88
f1 (ppm)

Compound **11**

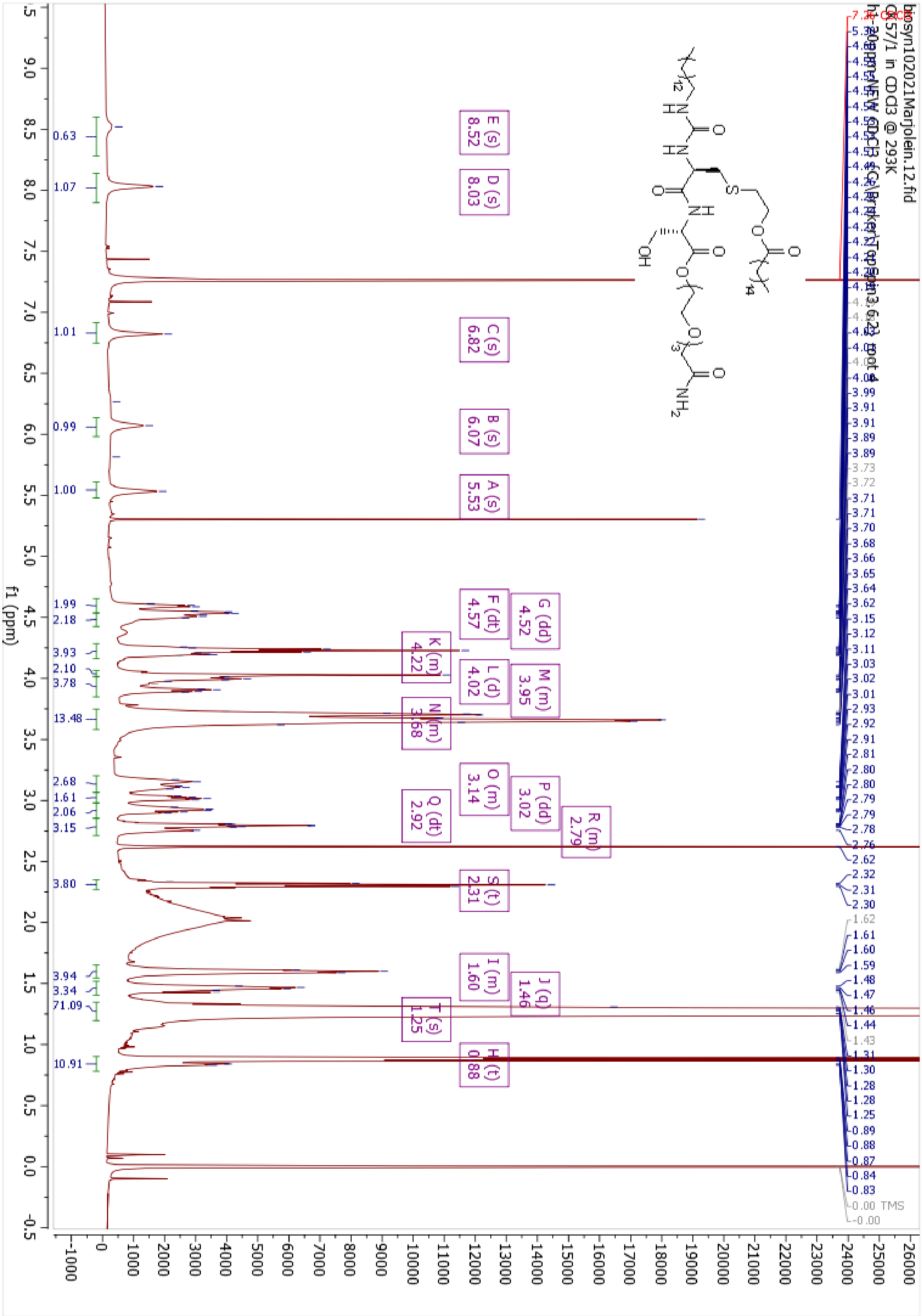

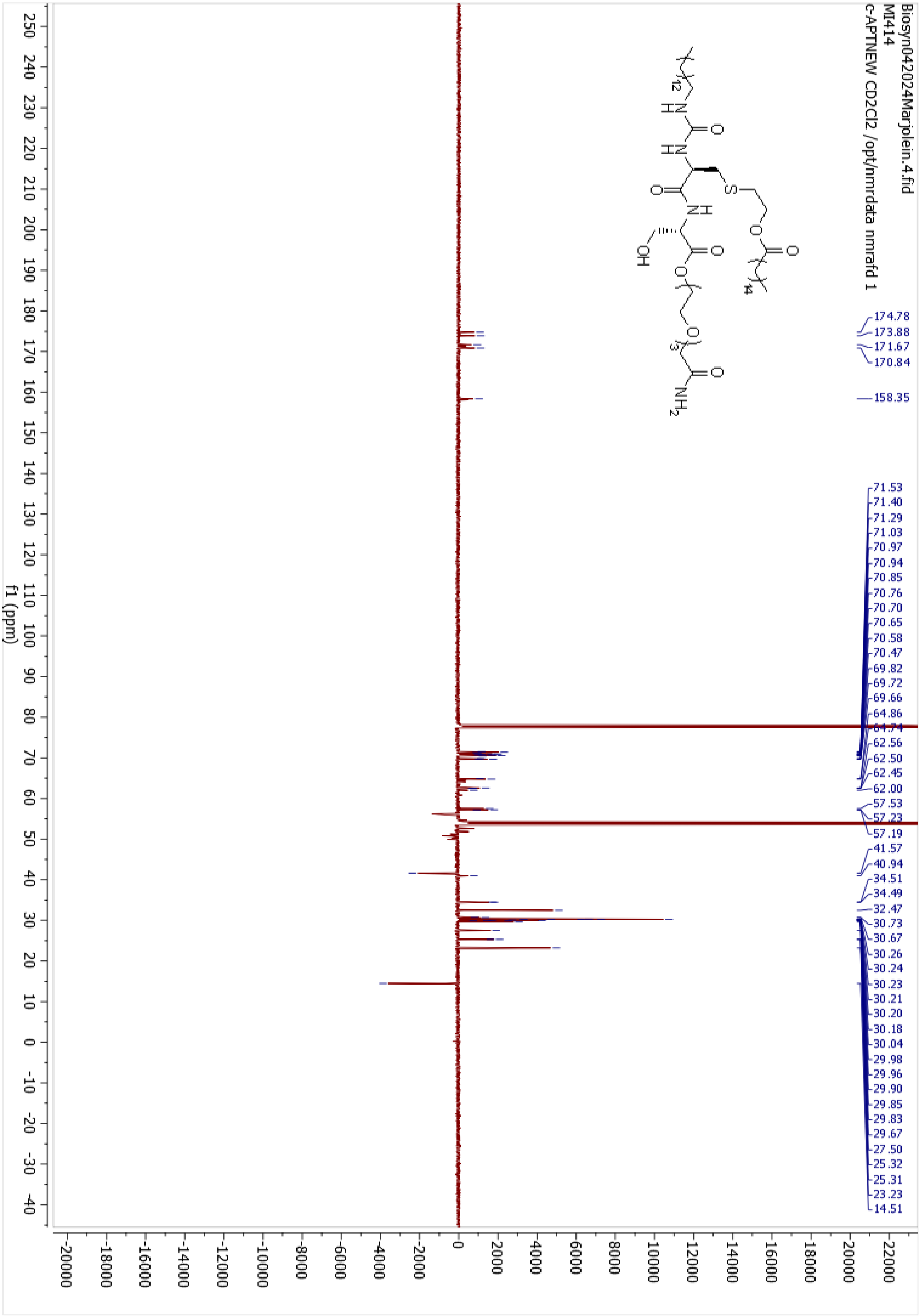
Biosyn042024Marjolein.4.fid
MI414
c-APTNEW CD2Cl2 /opt/nmrdata nmrafd 1
174.78
173.88
171.67
170.84
158.35
71.53
71.40
71.29
71.03
70.97
70.94
70.85
70.76
70.70
70.65
70.58
70.47
69.82
69.72
69.66
64.86
64.74
62.56
62.50
62.45
62.00
57.53
57.23
57.19
41.57
40.94
34.51
34.49
32.47
30.73
30.67
30.26
30.24
30.23
30.21
30.20
30.18
30.04
29.98
29.96
29.90
29.85
29.83
29.67
27.50
25.32
25.31
23.23
14.51
f1 (ppm)

Compound **19**

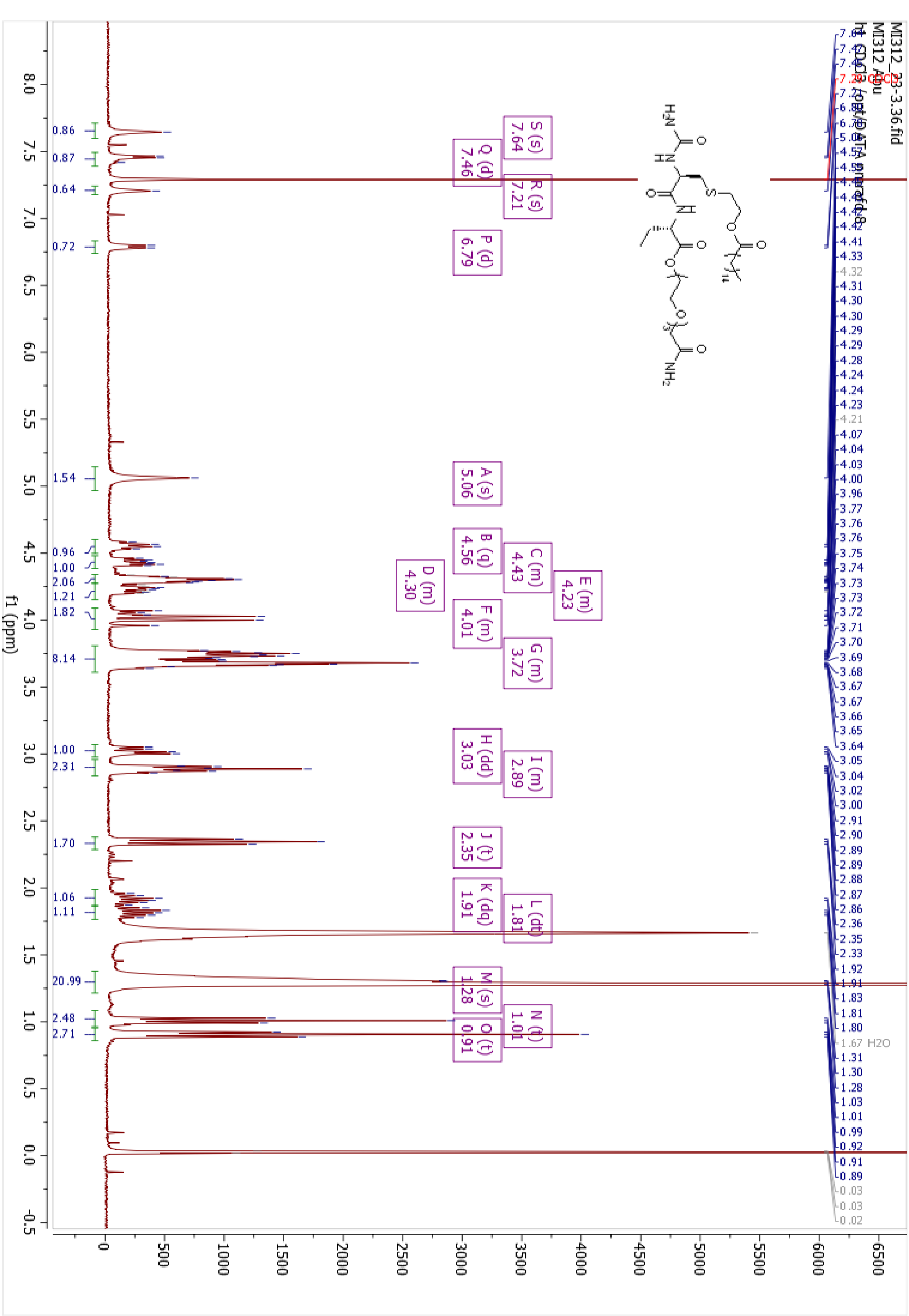

S (s) 7.64
Q (d) 7.46
R (s) 7.21
P (d) 6.79
A (s) 5.06
B (q) 4.56
C (m) 4.43
D (m) 4.30
E (m) 4.23
F (m) 4.01
G (m) 3.72
H (dd) 3.03
I (m) 2.89
J (t) 2.35
K (dq) 1.91
L (dt) 1.81
M (s) 1.28
N (t) 1.01
O (t) 0.91
f1 (ppm)

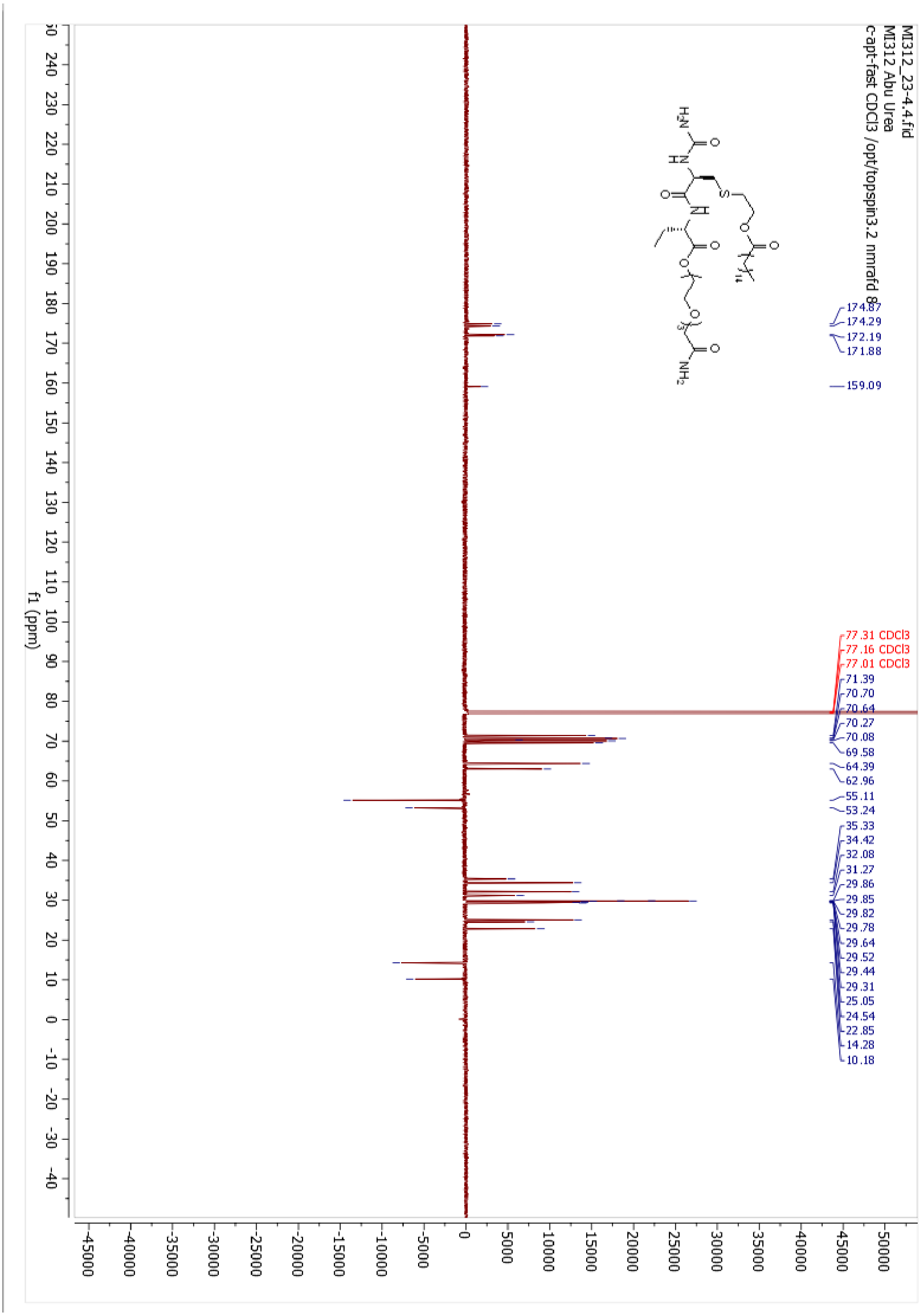
MI312_23-4.4.fid
MI312 Abu Urea
c-apt-fast CDCl3 /opt/topspin3.2 nmrafd 8
174.87
174.29
172.19
171.88
159.09
77.31 CDCl3
77.16 CDCl3
77.01 CDCl3
71.39
70.70
70.64
70.27
70.08
69.58
64.39
62.96
55.11
53.24
35.33
34.42
32.08
31.27
29.86
29.85
29.82
29.78
29.64
29.52
29.44
29.31
25.05
24.54
22.85
14.28
10.18
f1 (ppm)

Compound **20**

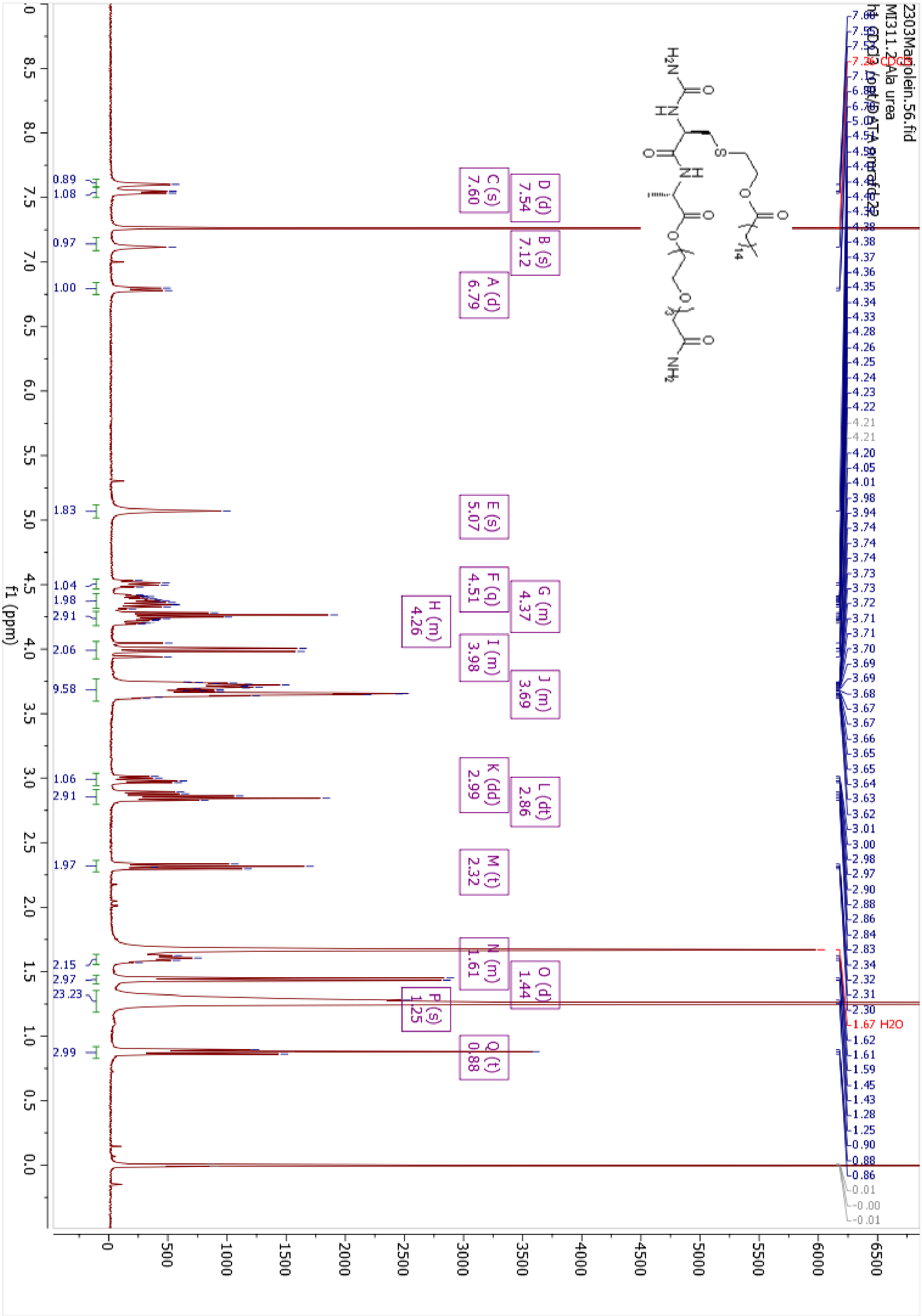

A (d) 6.79
B (s) 7.12
C (s) 7.60
D (d) 7.54
E (s) 5.07
F (q) 4.51
G (m) 4.37
H (m) 4.26
I (m) 3.98
J (m) 3.69
K (dd) 2.99
L (dt) 2.86
M (t) 2.32
N (m) 1.61
O (d) 1.44
P (s) 1.25
Q (t) 0.88
7.26 CDCl3
1.67 H2O
f1 (ppm)

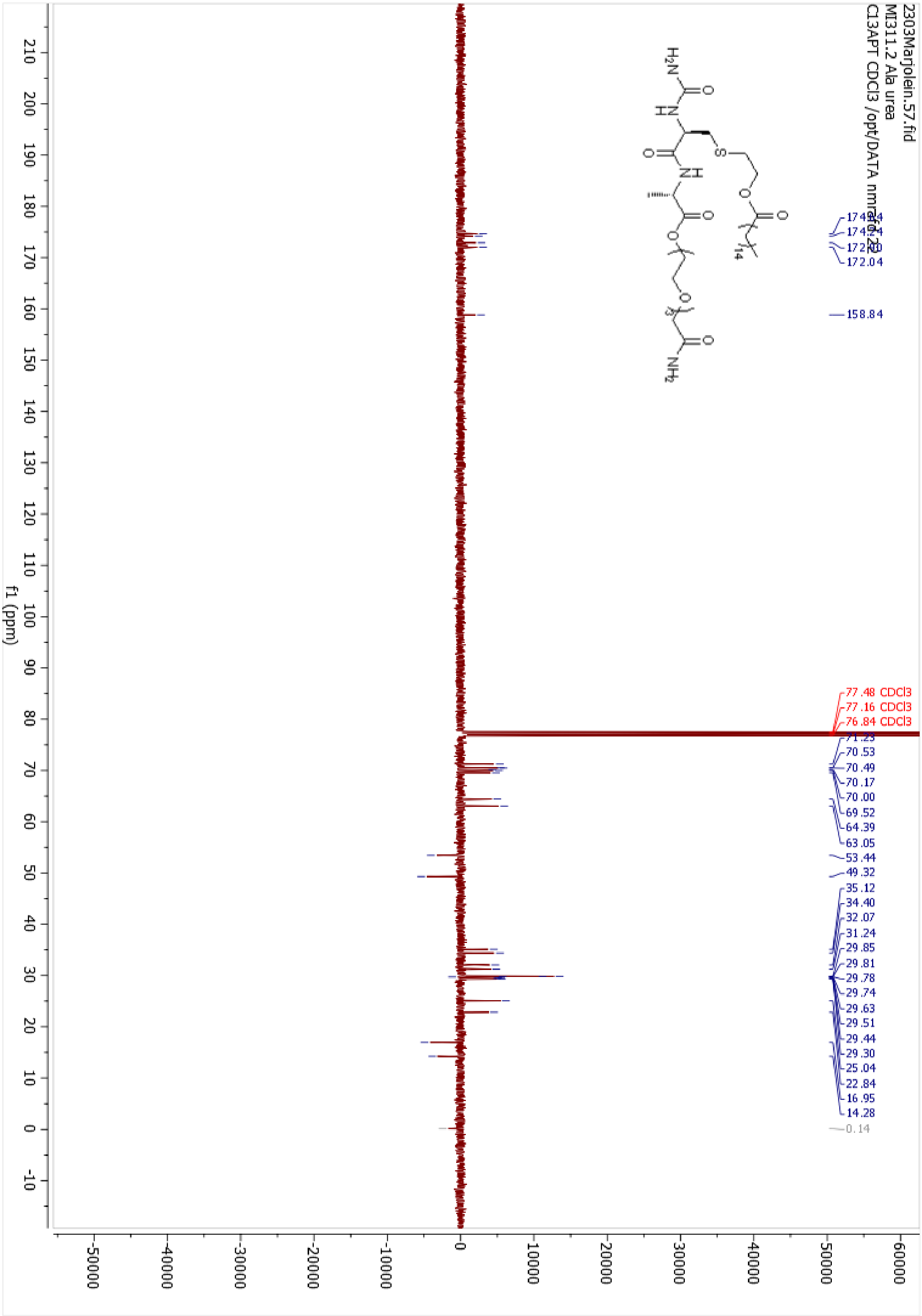

2303Marjolein.57.fid
MI311.2 Ala urea
174.24
172.04
158.84
77.48 CDCl3
77.16 CDCl3
76.84 CDCl3
70.53
70.49
70.17
70.00
69.52
64.39
63.05
53.44
49.32
35.12
34.40
32.07
31.24
29.85
29.81
29.78
29.74
29.63
29.51
29.44
29.30
25.04
22.84
16.95
14.28
0.14
f1 (ppm)

LCMS figures synthetic ligands

Compound **1**

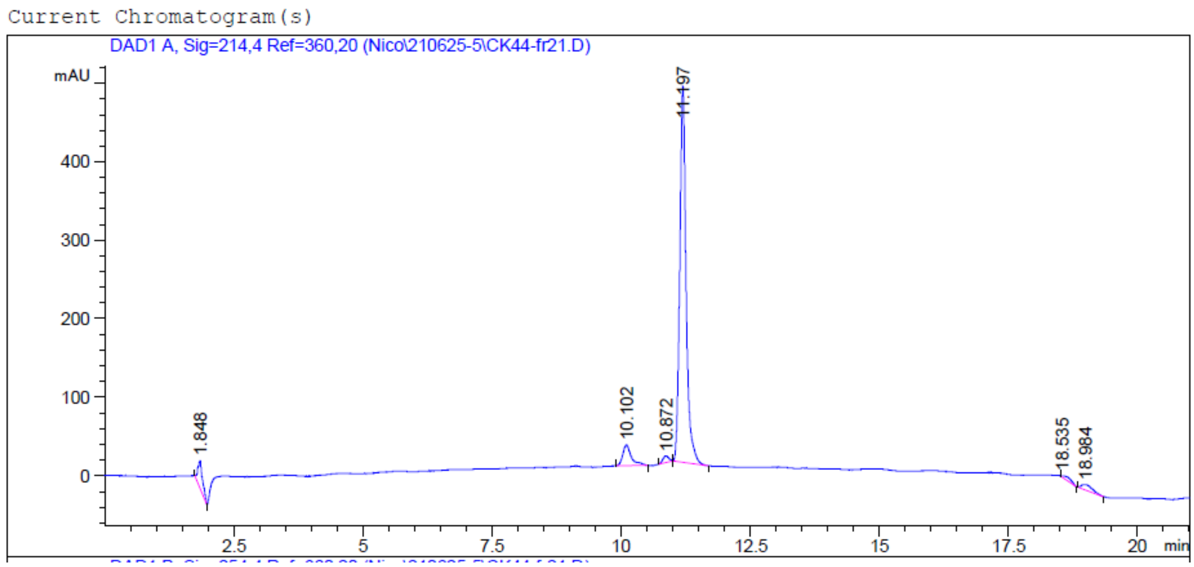

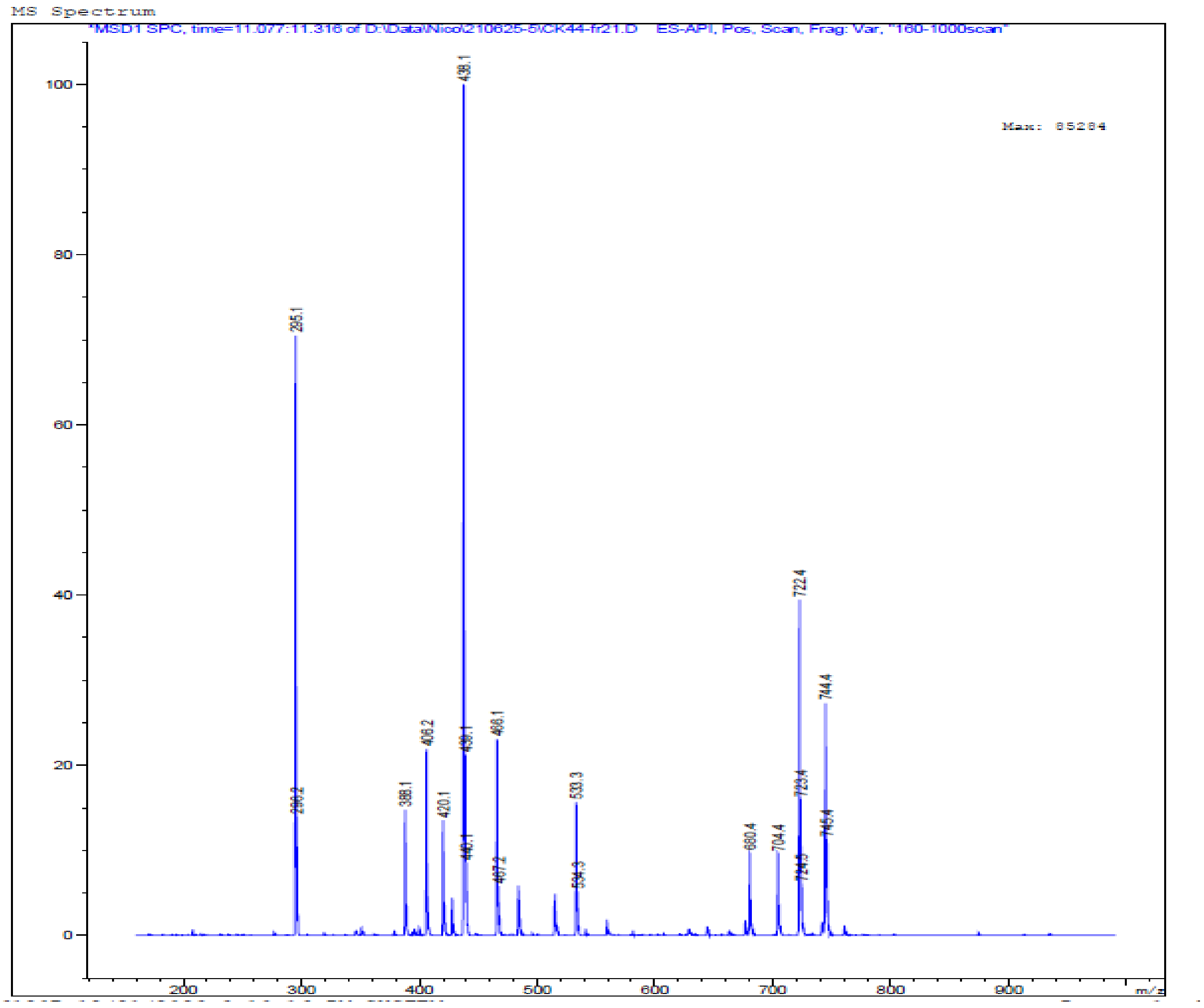

Compound **2**

Current Chromatogram(s)

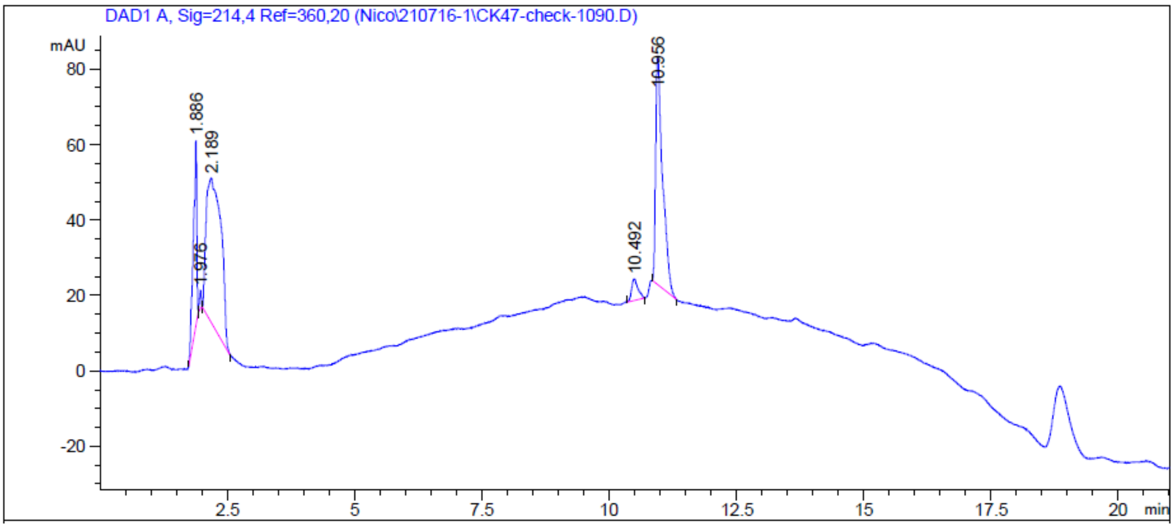

MS Spectrum

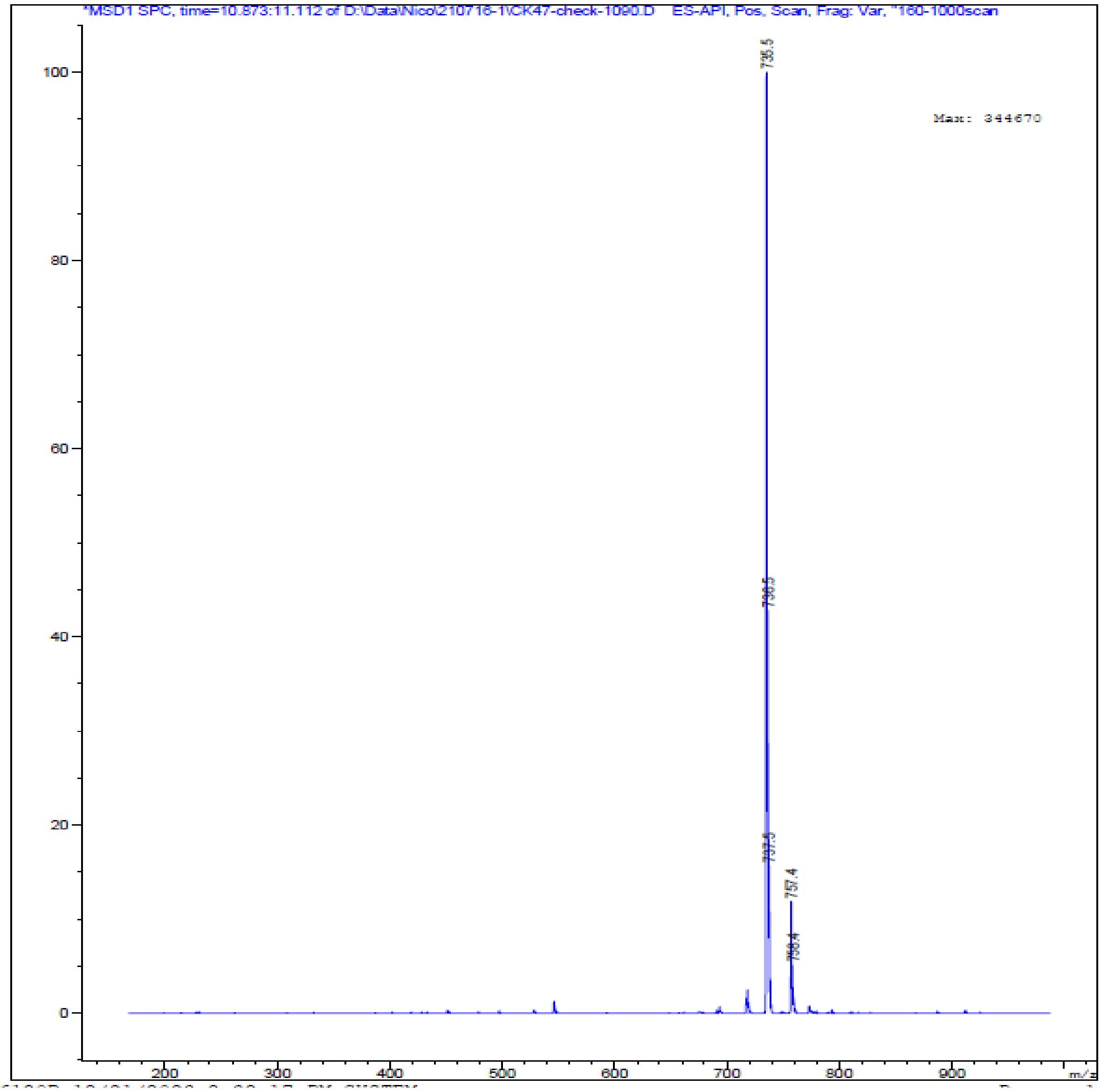

Compound **3**

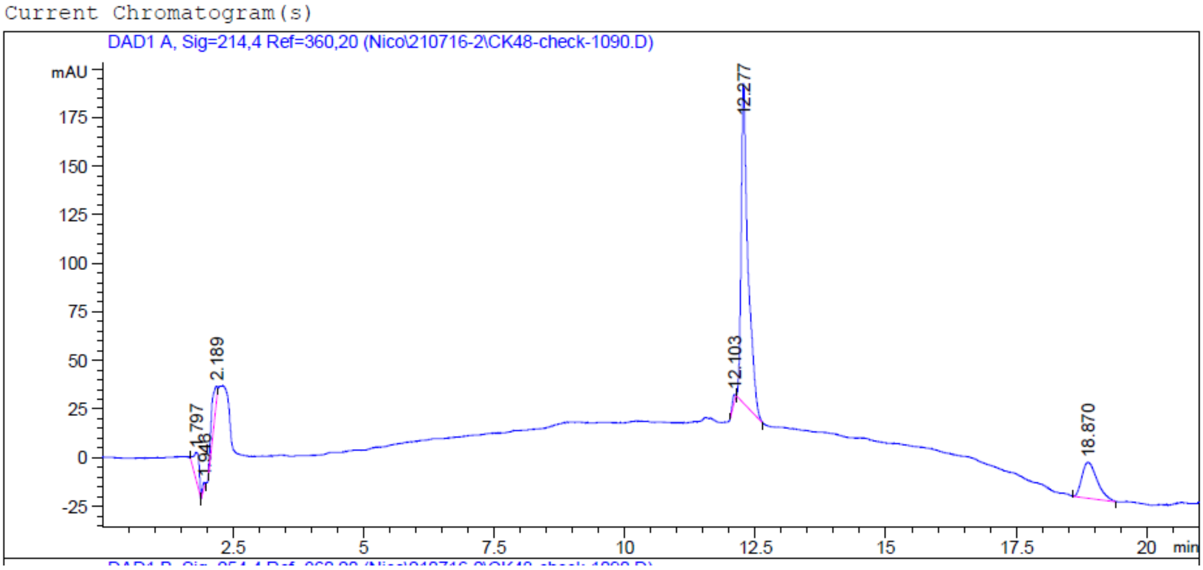

Current Chromatogram(s)
DAD1 A, Sig=214,4 Ref=360,20 (Nico\210716-2\CK48-check-1090.D)
mAU
175
150
125
100
75
50
25
0
-25
2.5
5
7.5
10
12.5
15
17.5
20
min
1.797
1.948
2.189
12.103
12.277
18.870

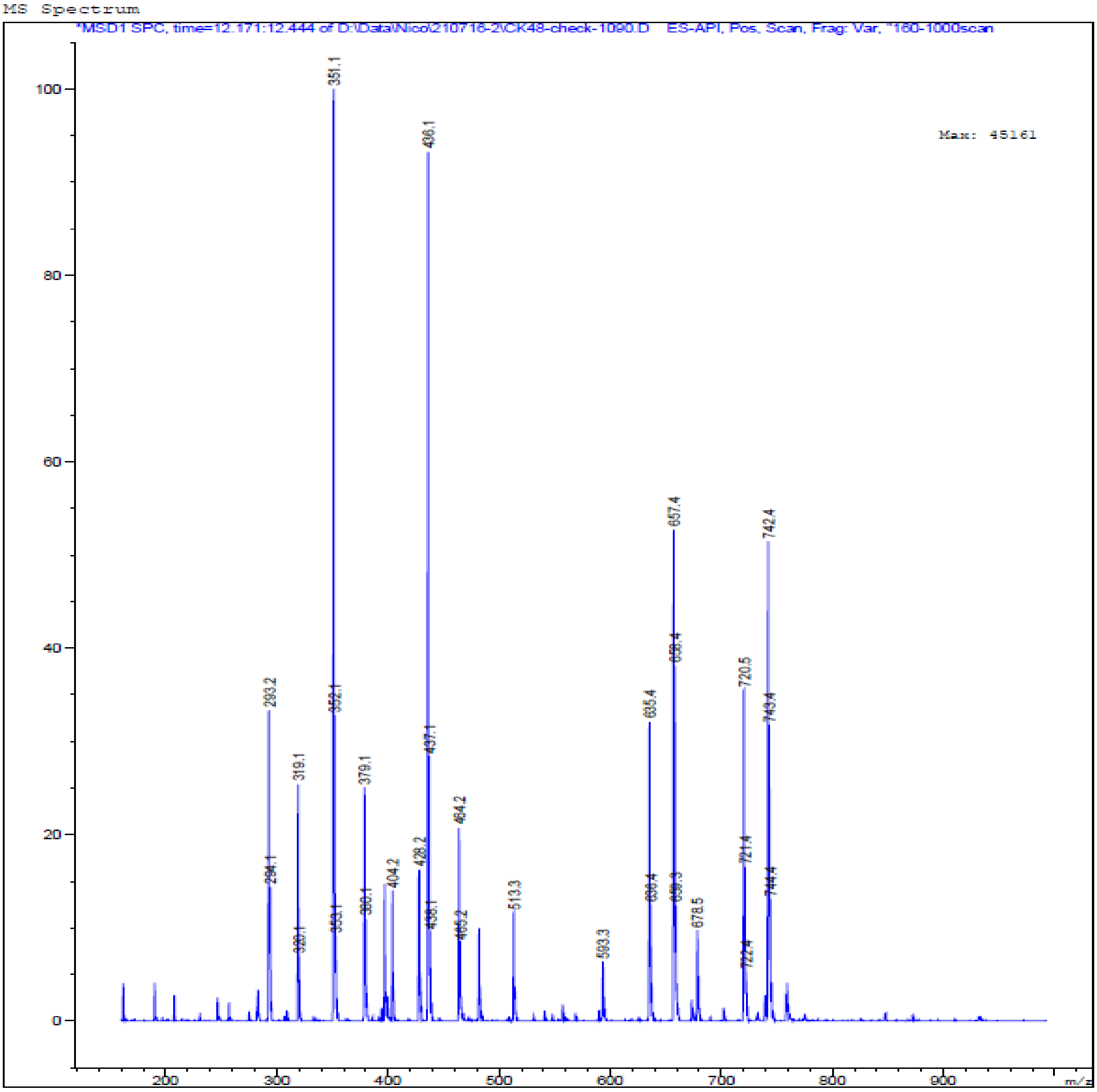

MS Spectrum
*MSD1 SPC, time=12.171:12.444 of D:\Data\Nico\210716-2\CK48-check-1090.D ES-API, Pos, Scan, Frag: Var, "160-1000scan
Max: 45161
100
80
60
40
20
0
200
300
400
500
600
700
800
900
351.1
436.1
657.4
742.4
293.2
319.1
379.1
437.1
464.2
635.4
720.5
743.4
404.2
428.2
513.3
593.3
678.5

Compound **4**

Current Chromatogram(s)

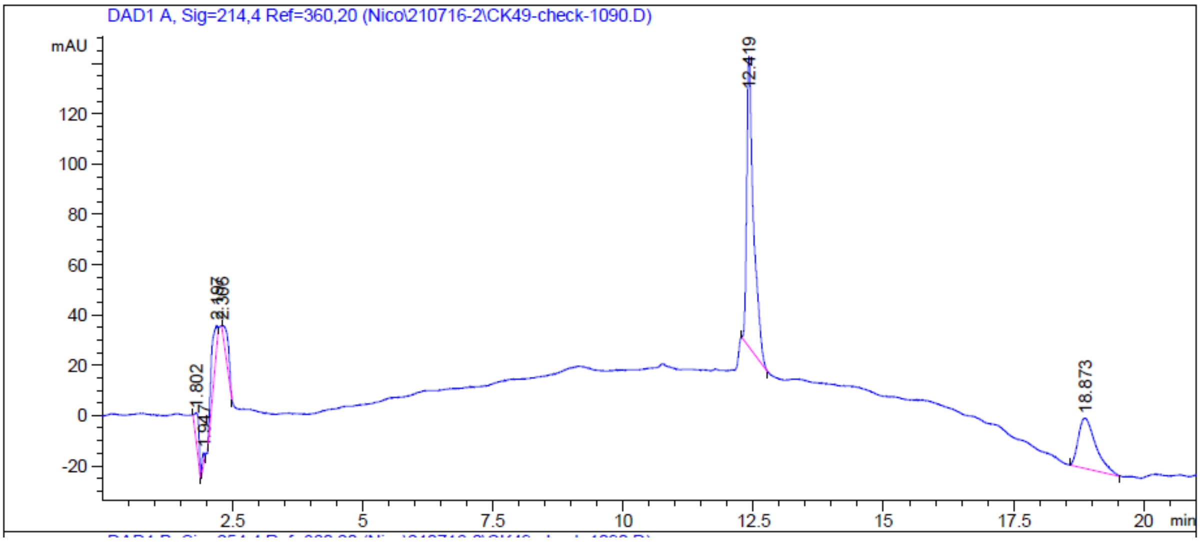

MS Spectrum

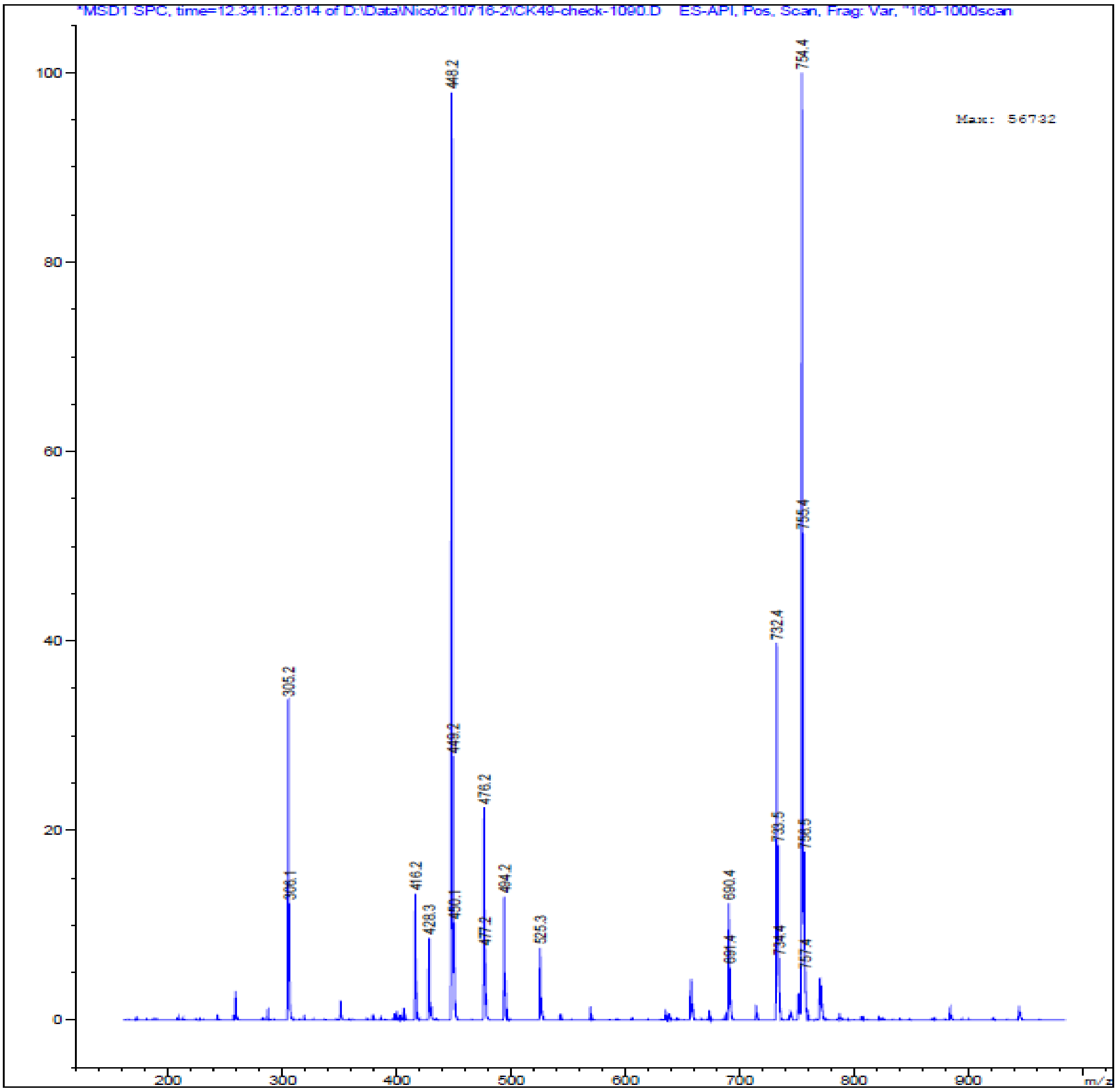

Compound **5**

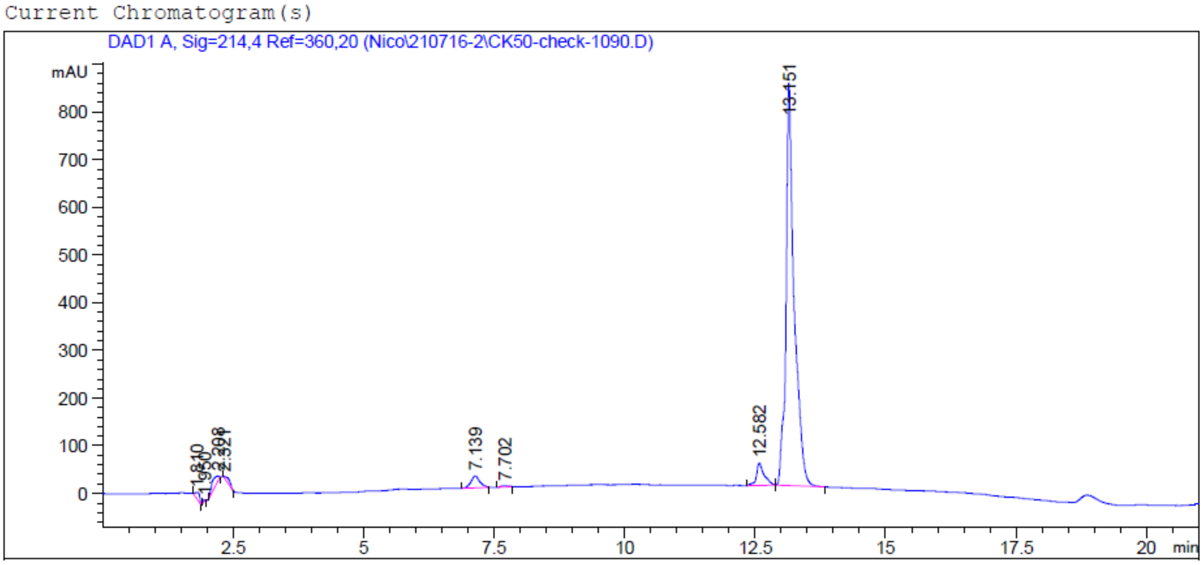
Current Chromatogram(s)
DAD1 A, Sig=214,4 Ref=360,20 (Nico\210716-2\CK50-check-1090.D)
mAU
13.151
12.582
7.139
7.702
min

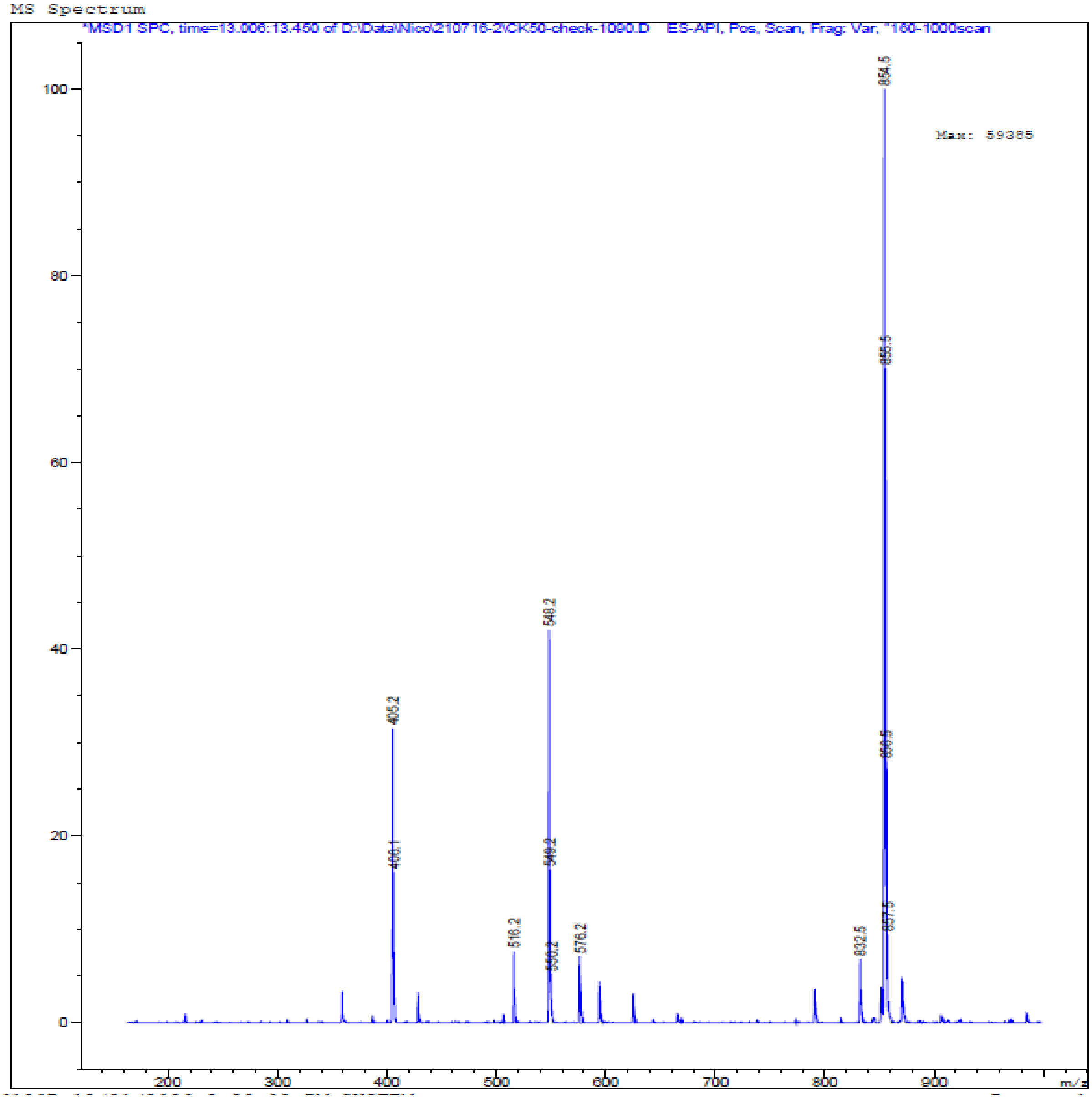
MS Spectrum
*MSD1 SPC, time=13.006:13.450 of D:\Data\Nico\210716-2\CK50-check-1090.D ES-API, Pos, Scan, Frag: Var, "160-1000scan
Max: 59385
854.5
855.5
548.2
405.2
406.1
549.2
516.2
576.2
832.5
m/z

Compound **6**

Current Chromatogram(s)

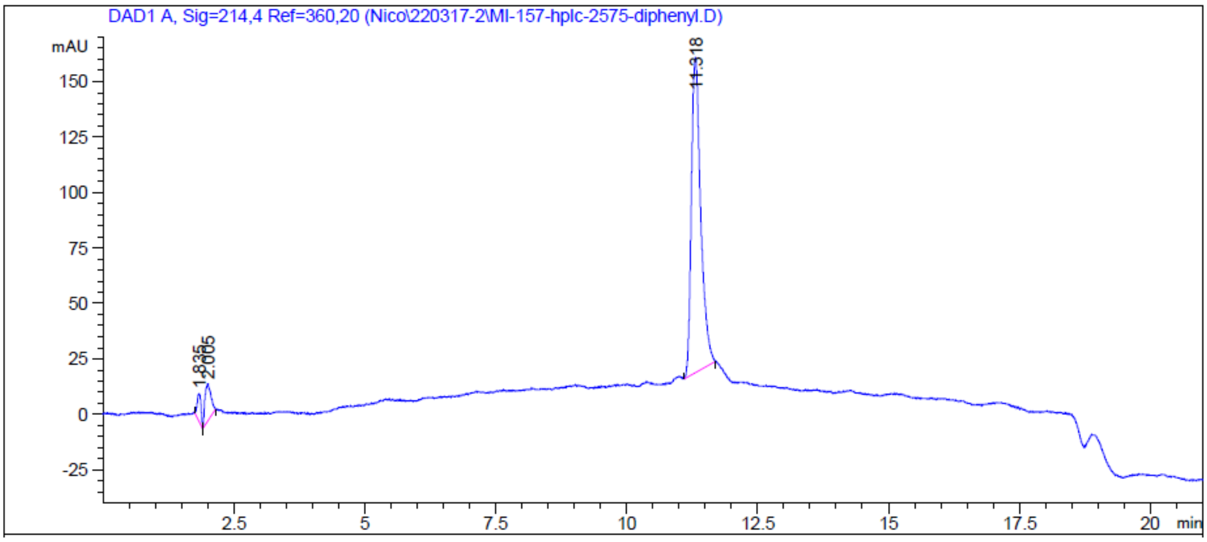

MS Spectrum

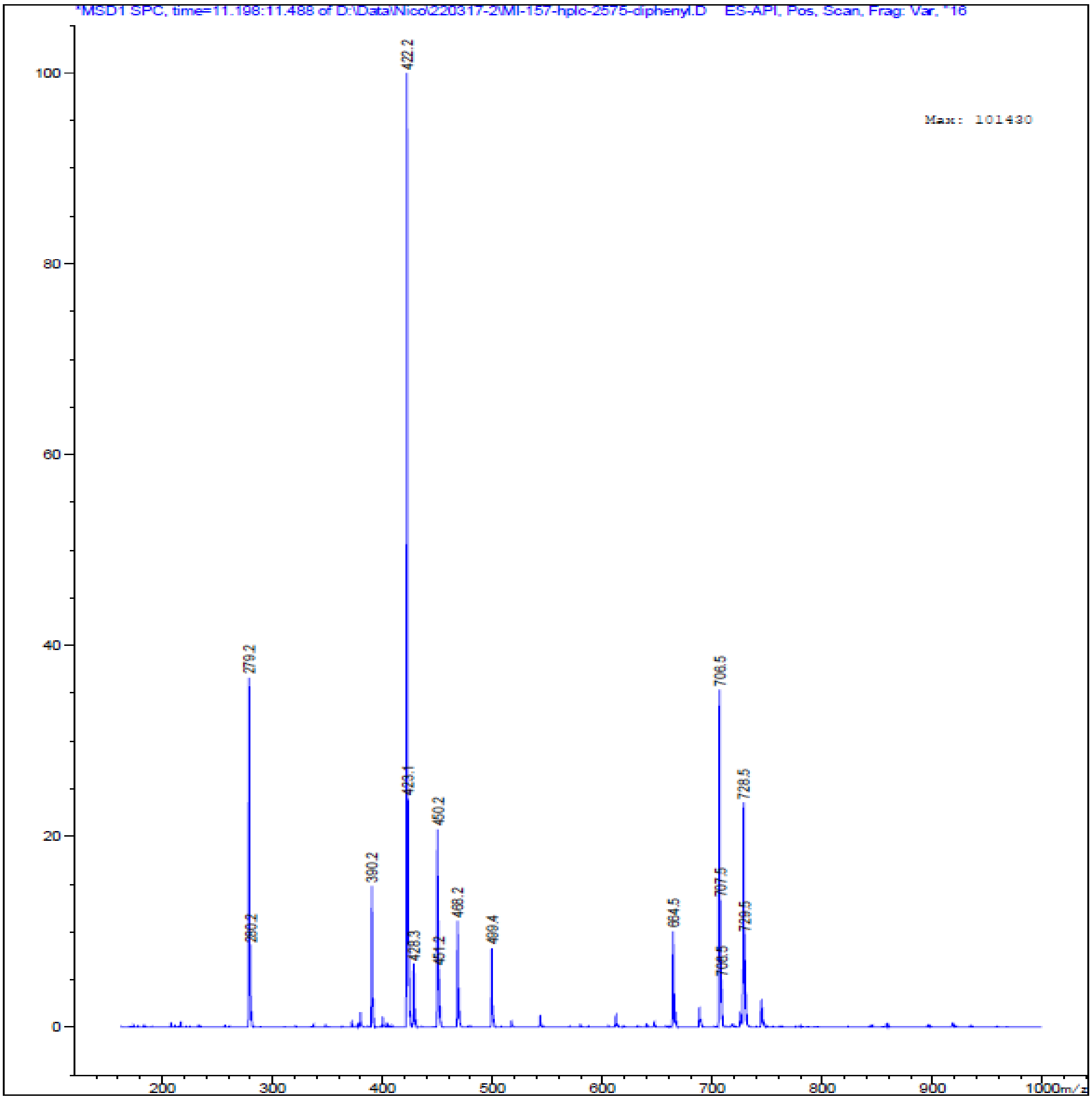

Compound **7**

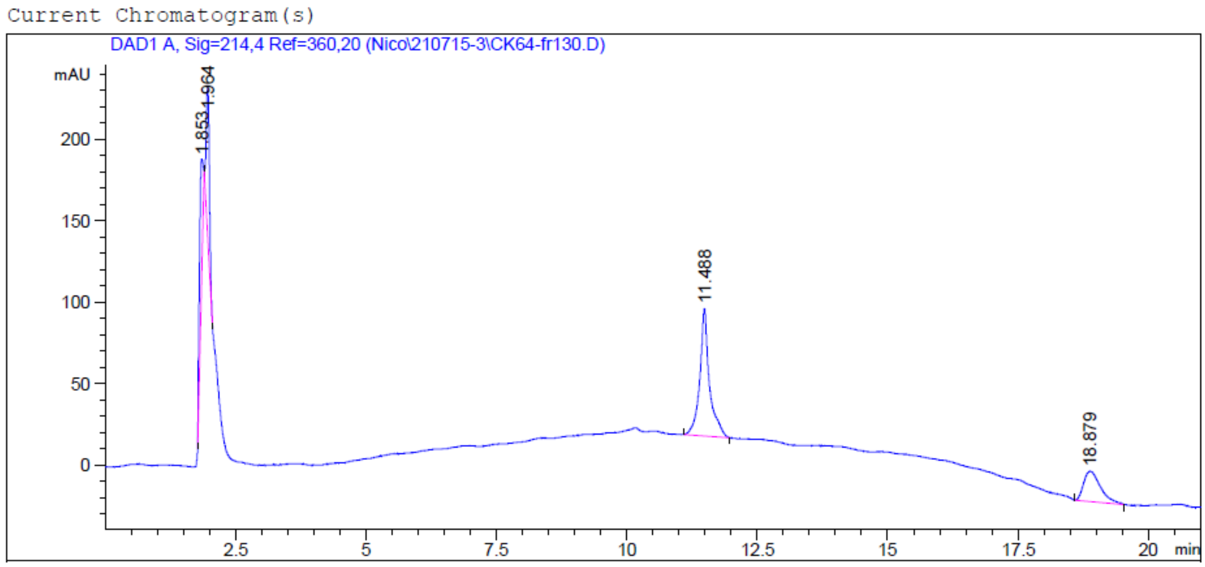
Current Chromatogram(s)
DAD1 A, Sig=214,4 Ref=360,20 (Nico\210715-3\CK64-fr130.D)
mAU
1.853
1.964
11.488
18.879
min

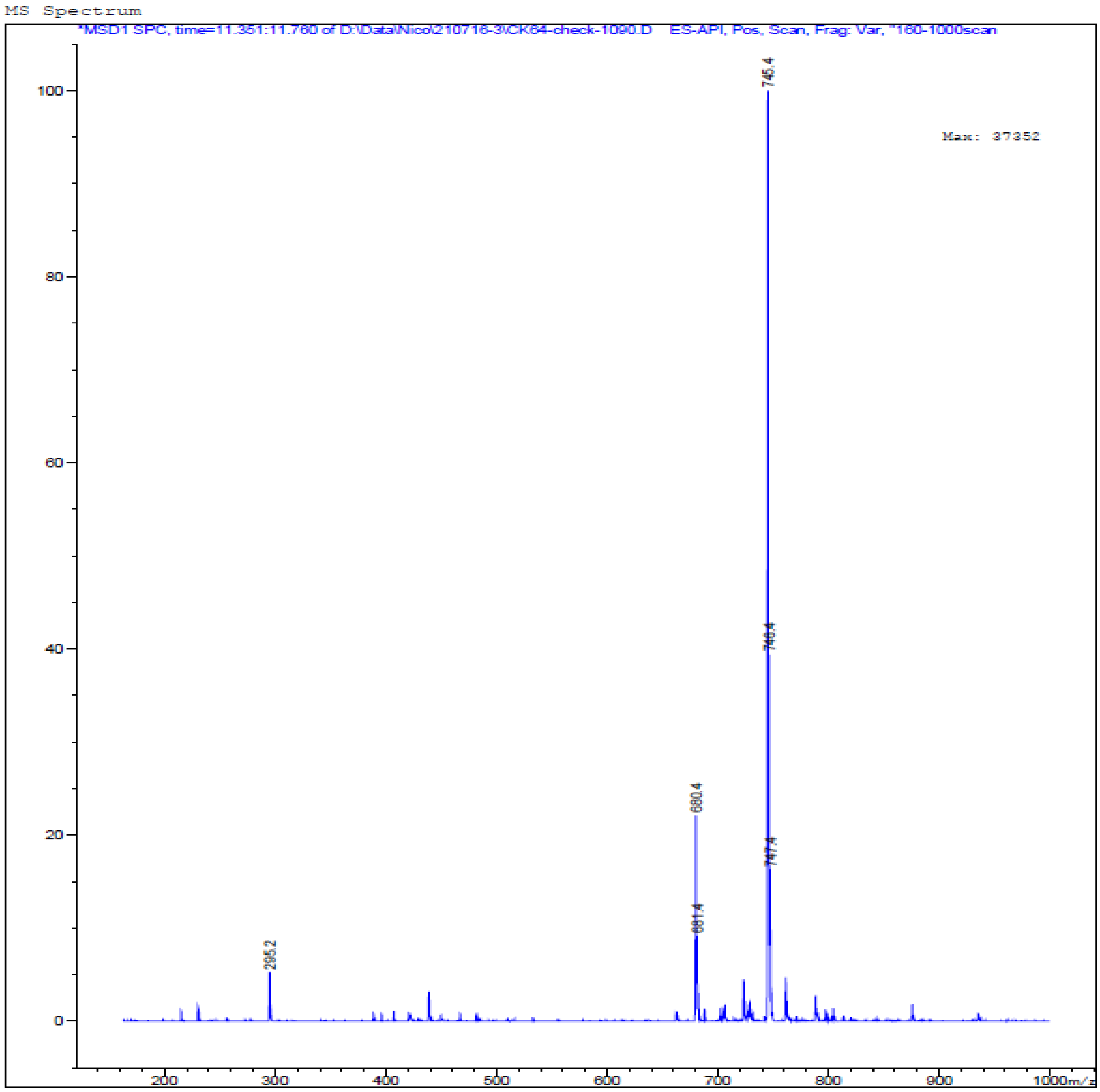
MS Spectrum
*MSD1 SPC, time=11.351:11.760 of D:\Data\Nico\210716-3\CK64-check-1090.D ES-API, Pos, Scan, Frag: Var, "160-1000scan
Max: 37352
745.4
746.4
747.4
680.4
681.4
295.2
m/z

Compound **8**

Current Chromatogram(s)

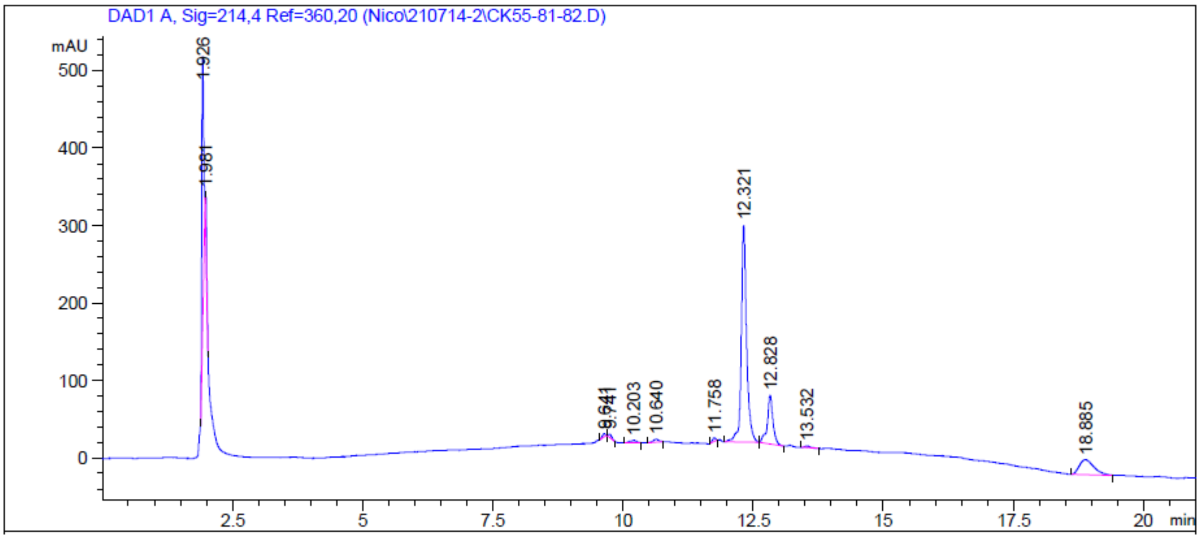

MS Spectrum

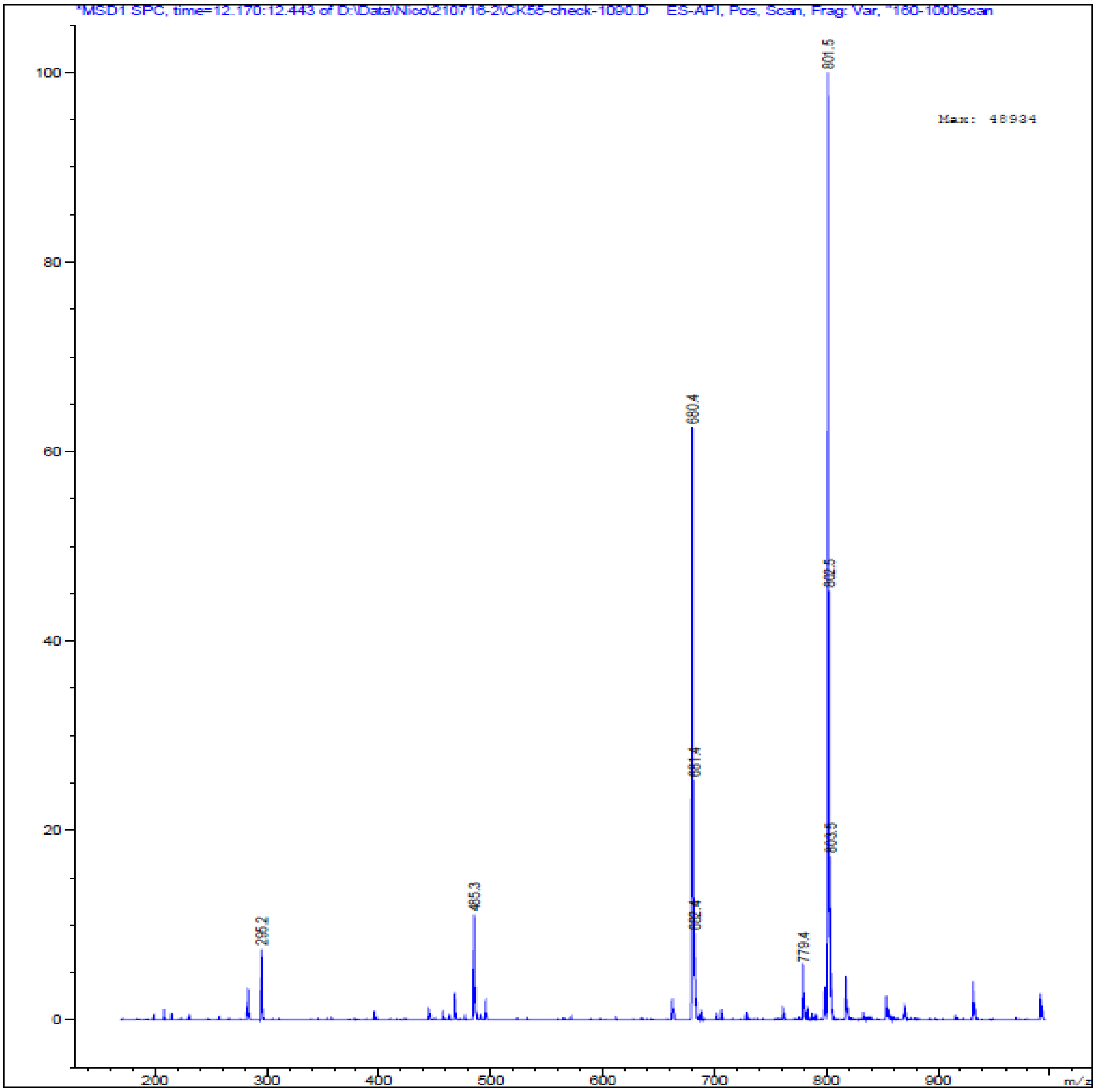

Compound **9**

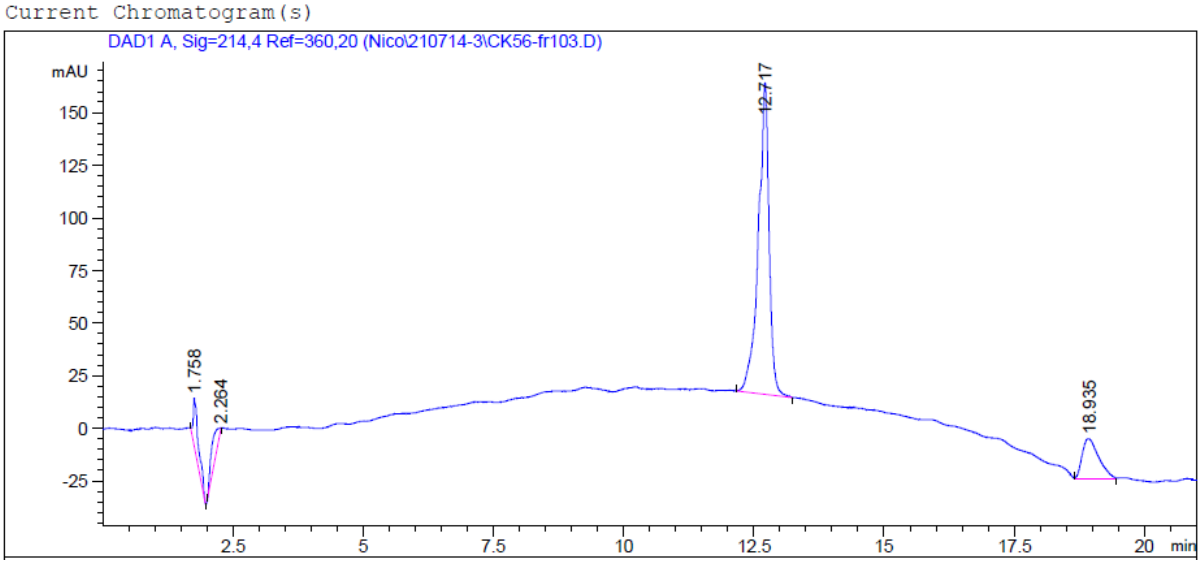

Current Chromatogram(s)
DAD1 A, Sig=214,4 Ref=360,20 (Nico\210714-3\CK56-fr103.D)
mAU
150
125
100
75
50
25
0
-25
1.758
2.264
12.717
18.935
2.5
5
7.5
10
12.5
15
17.5
20
min

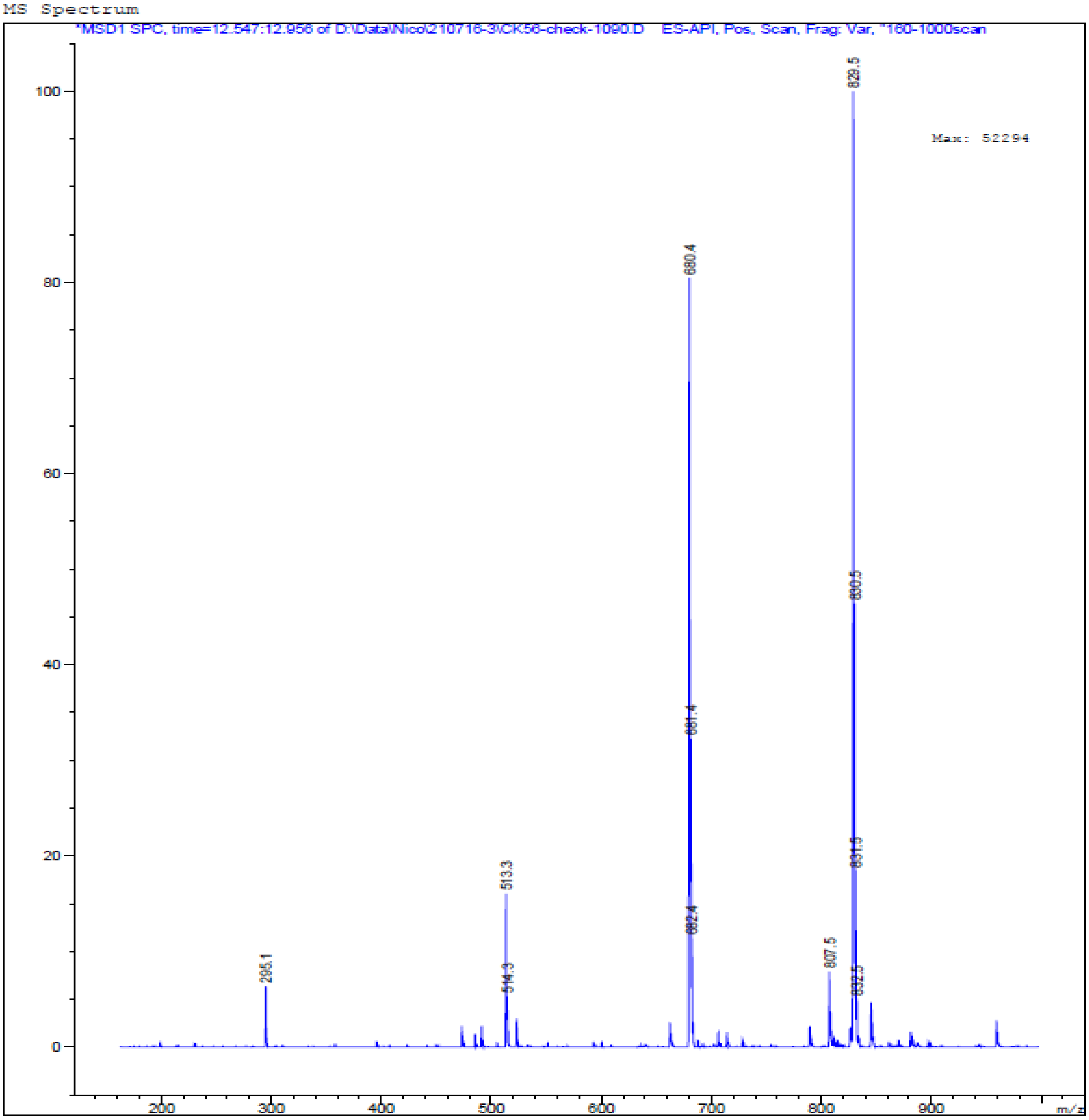

MS Spectrum
*MSD1 SPC, time=12.547:12.956 of D:\Data\Nico\210716-3\CK56-check-1090.D ES-API, Pos, Scan, Frag: Var, "160-1000scan
Max: 52294
100
80
60
40
20
0
295.1
513.3
514.3
680.4
681.4
682.4
807.5
829.5
830.5
831.5
832.5
200
300
400
500
600
700
800
900
m/z

Compound **10**

Current Chromatogram(s)

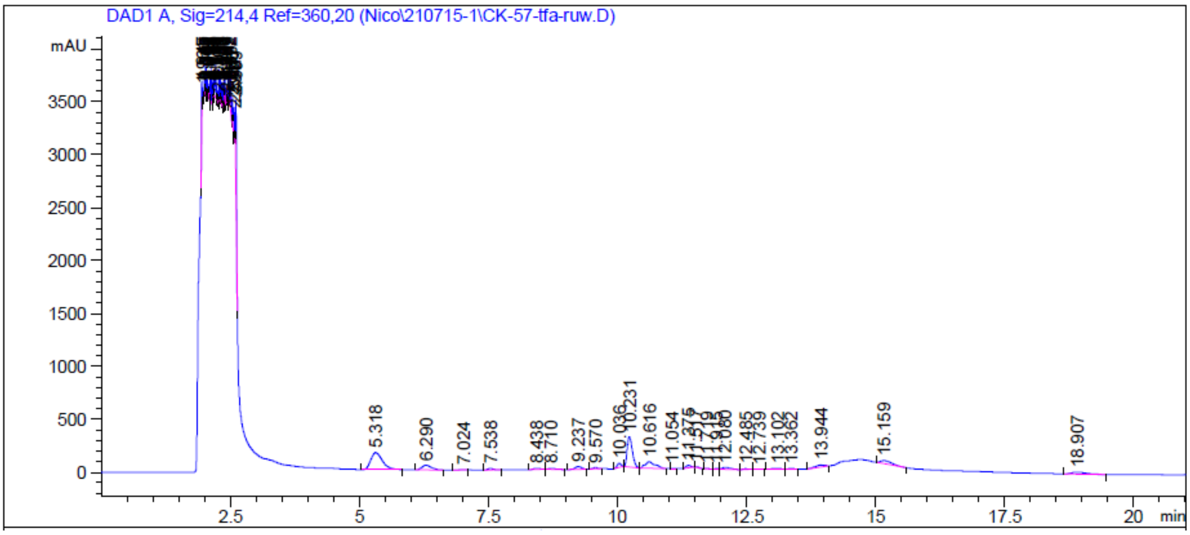

MS Spectrum

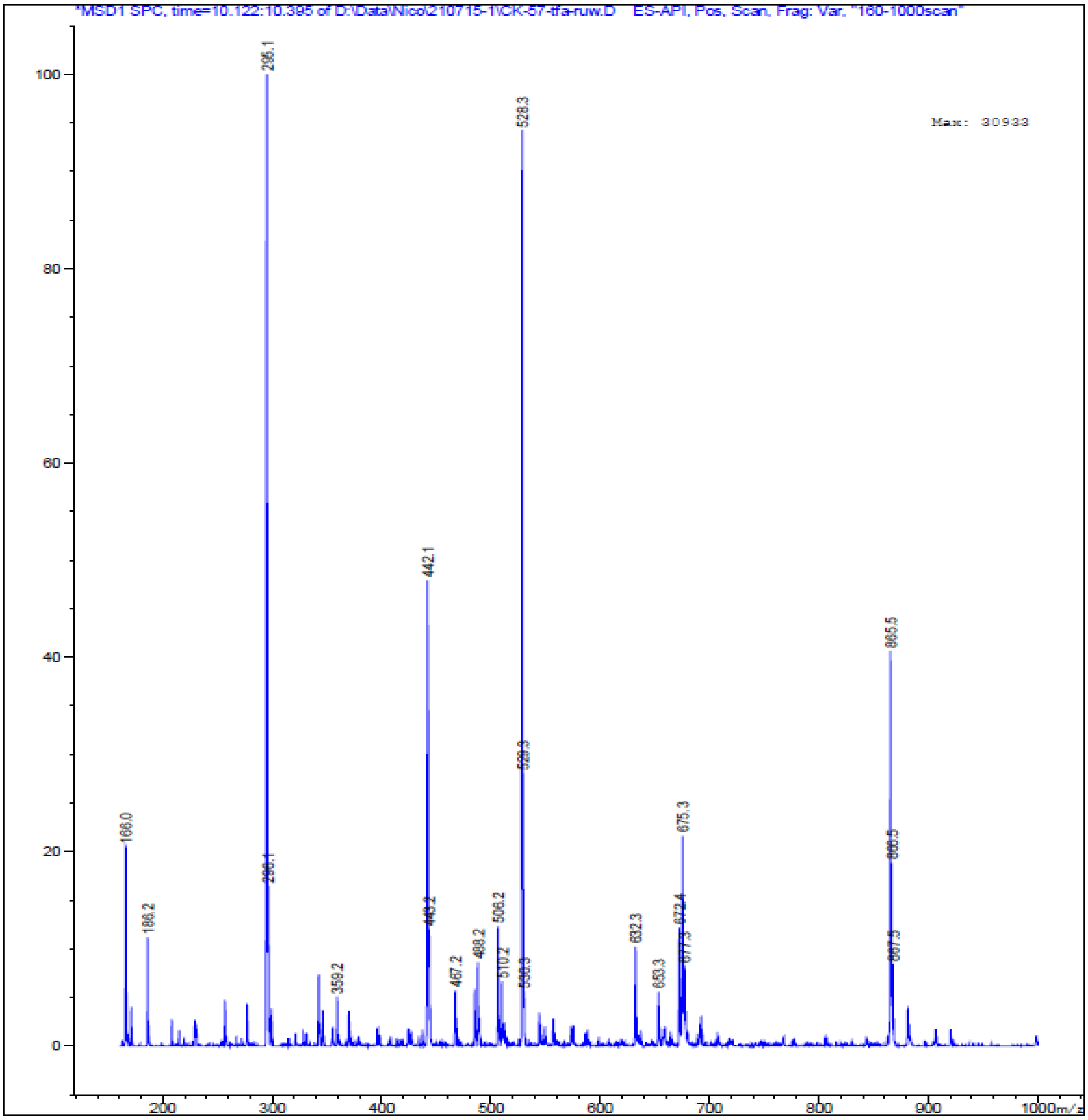

Compound **11**

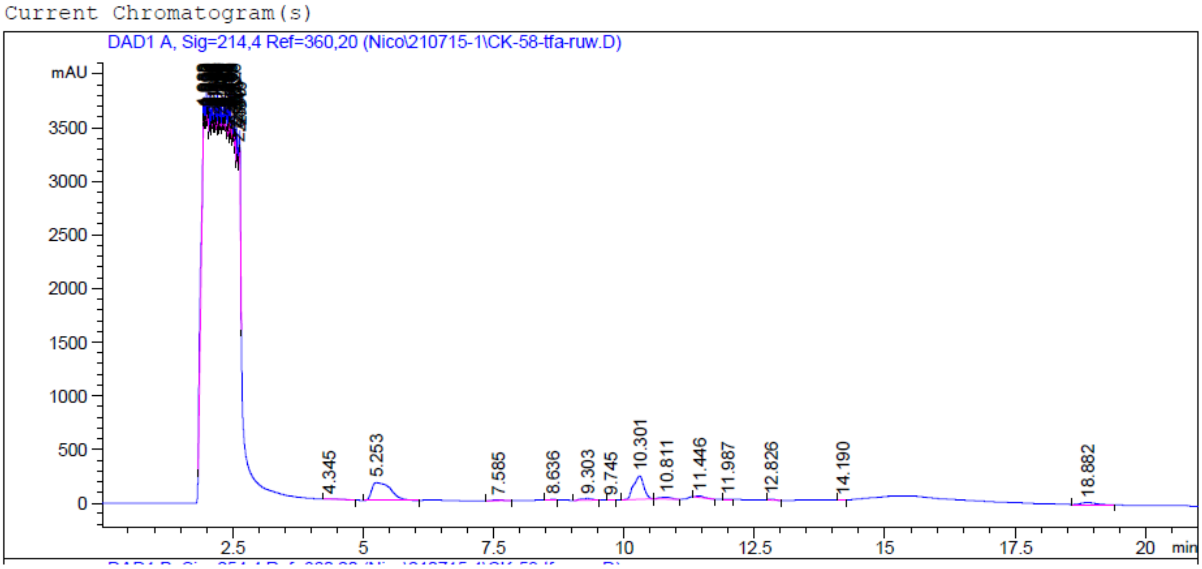
Current Chromatogram(s)
DAD1 A, Sig=214,4 Ref=360,20 (Nico\210715-1\CK-58-tfa-ruw.D)
mAU
4.345
5.253
7.585
8.636
9.303
9.745
10.301
10.811
11.446
11.987
12.826
14.190
18.882
min

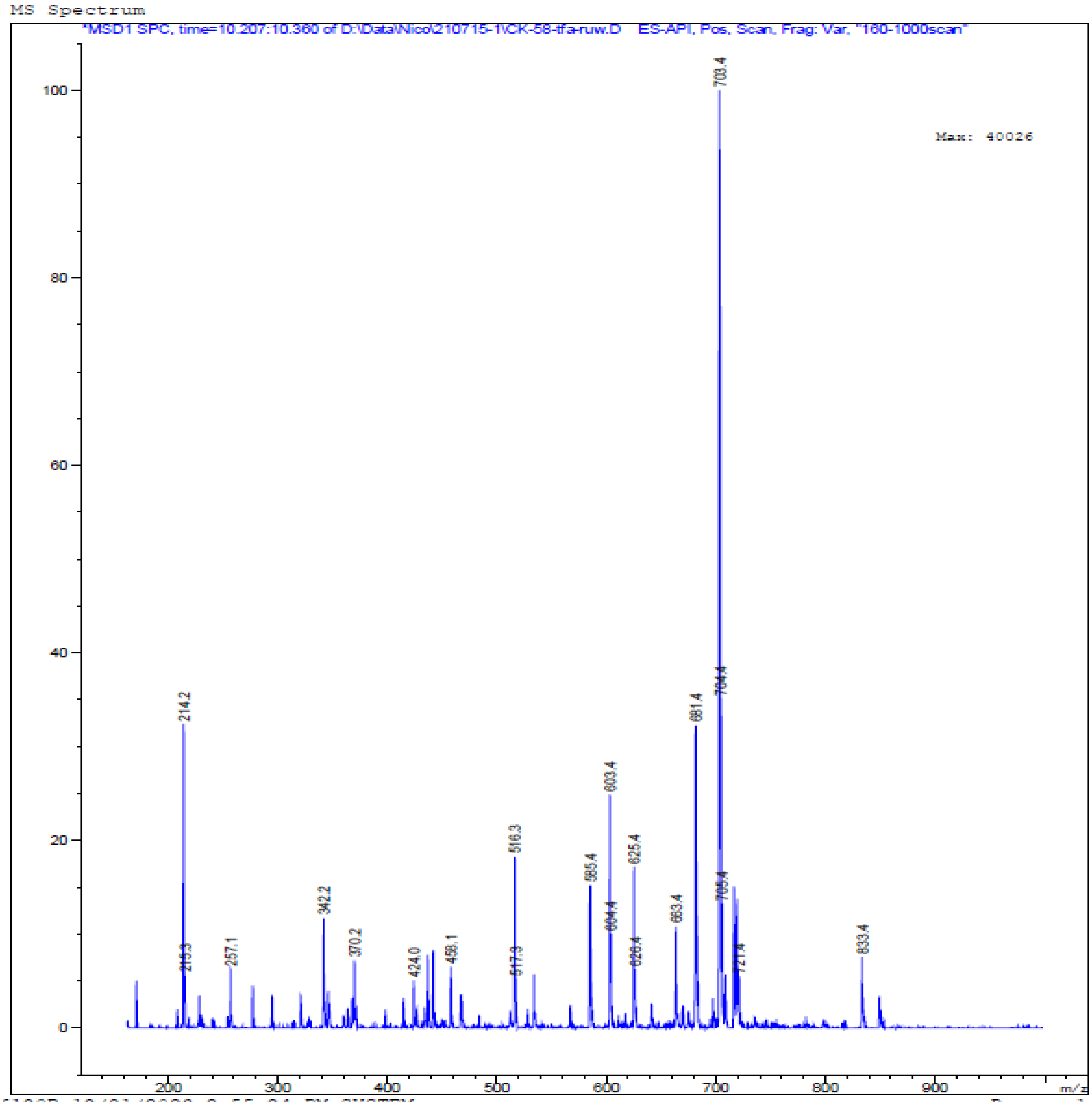
MS Spectrum
*MSD1 SPC, time=10.207:10.360 of D:\Data\Nico\210715-1\CK-58-tfa-ruw.D ES-API, Pos, Scan, Frag: Var, "160-1000scan"
Max: 40026
703.4
704.4
681.4
214.2
603.4
516.3
625.4
585.4
705.4
342.2
663.4
604.4
833.4
257.1
370.2
458.1
424.0
517.3
626.4
721.4
m/z

Compound **19**

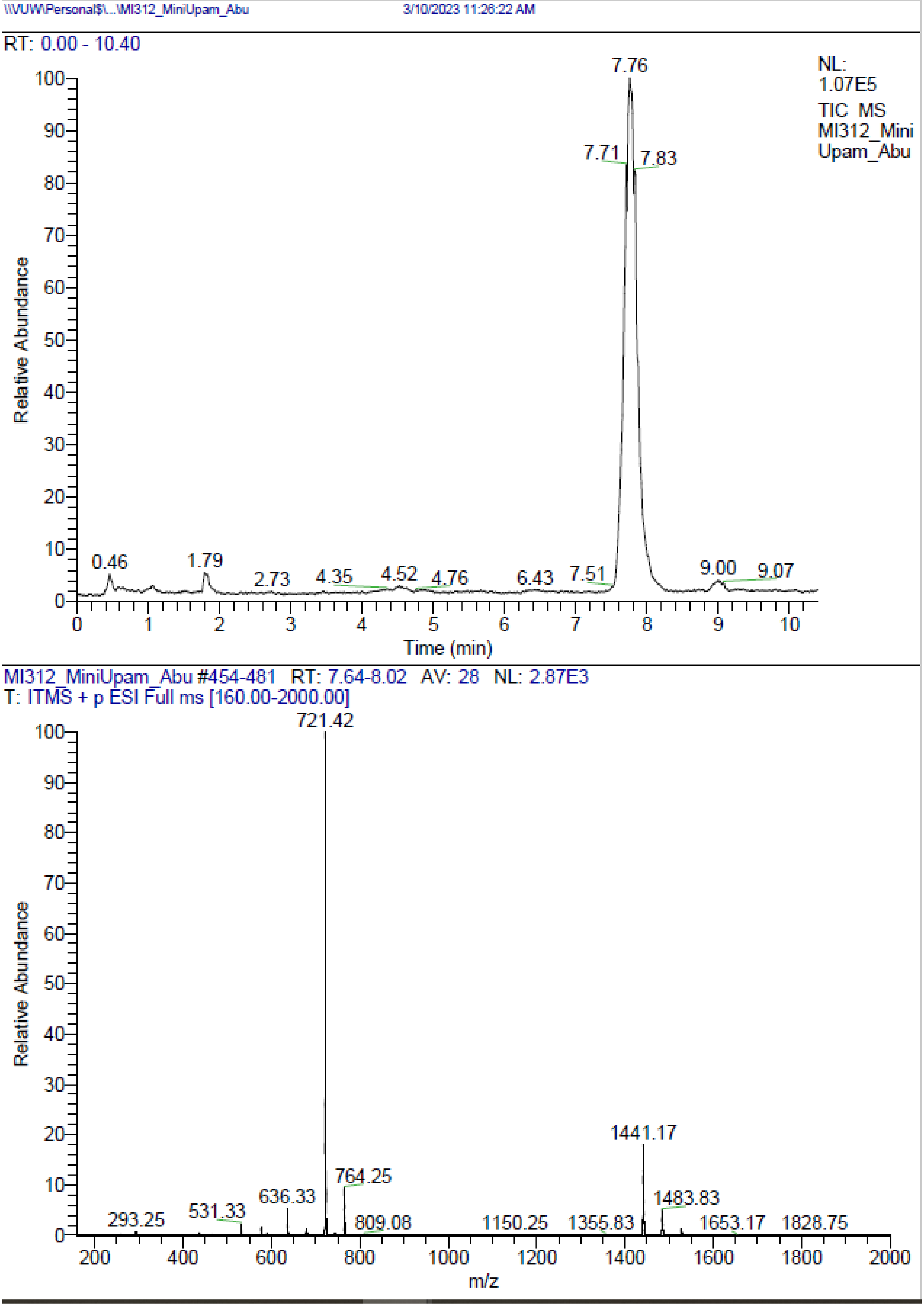

\\VUW\Personal$\...\MI312_MiniUpam_Abu
3/10/2023 11:26:22 AM
RT: 0.00 - 10.40
NL: 1.07E5
TIC MS MI312_MiniUpam_Abu
7.76
7.71
7.83
0.46
1.79
2.73
4.35
4.52
4.76
6.43
7.51
9.00
9.07
Relative Abundance
Time (min)
MI312_MiniUpam_Abu #454-481 RT: 7.64-8.02 AV: 28 NL: 2.87E3
T: ITMS + p ESI Full ms [160.00-2000.00]
721.42
1441.17
764.25
636.33
1483.83
531.33
293.25
809.08
1150.25
1355.83
1653.17
1828.75
Relative Abundance
m/z

Compound **20**

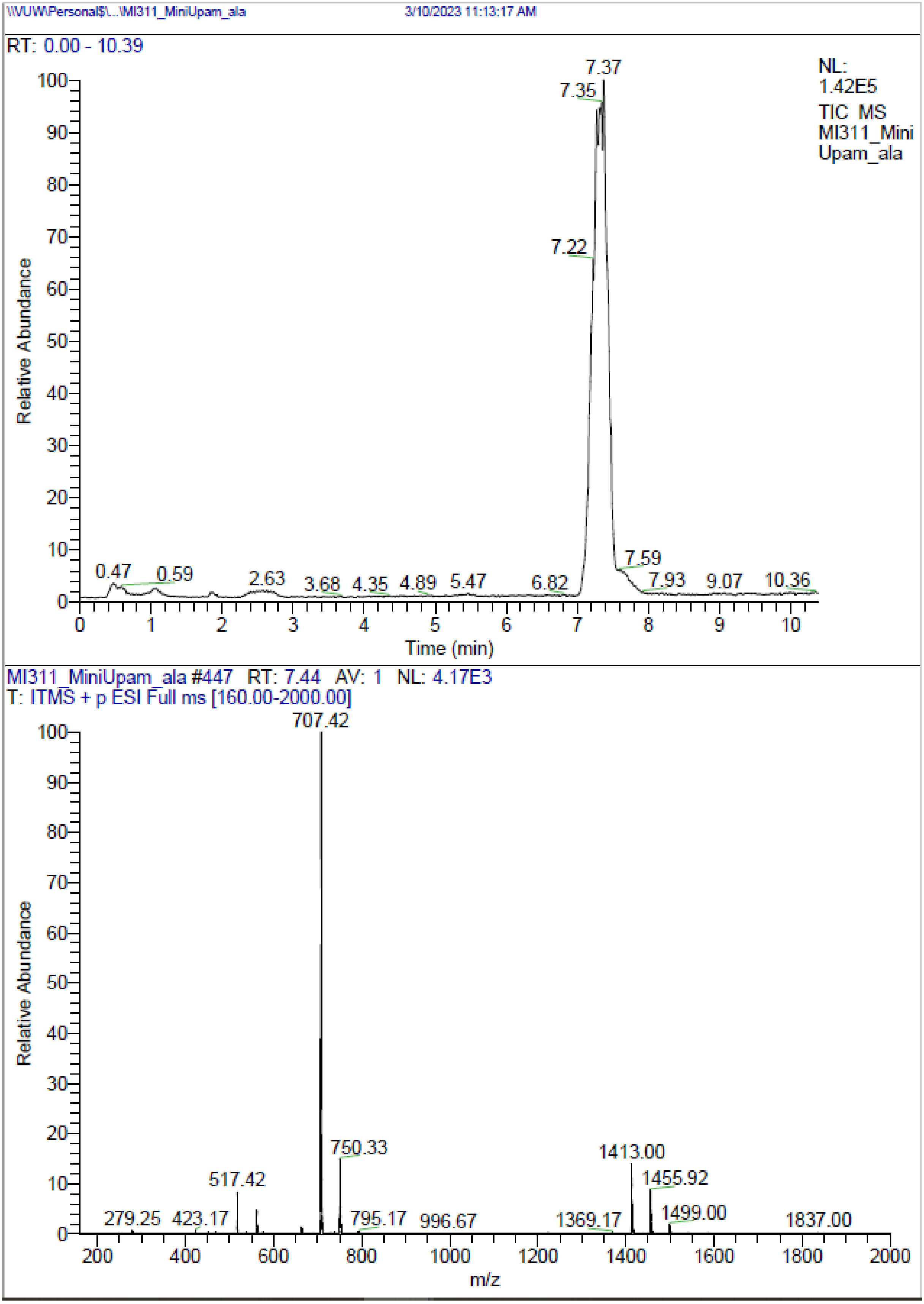
\\VUW\Personal$\...\MI311_MiniUpam_ala
3/10/2023 11:13:17 AM
RT: 0.00 - 10.39
NL: 1.42E5
TIC MS MI311_MiniUpam_ala
7.37
7.35
7.22
7.59
7.93
9.07
10.36
0.47
0.59
2.63
3.68
4.35
4.89
5.47
6.82
Relative Abundance
Time (min)
MI311_MiniUpam_ala #447 RT: 7.44 AV: 1 NL: 4.17E3
T: ITMS + p ESI Full ms [160.00-2000.00]
707.42
750.33
517.42
279.25
423.17
795.17
996.67
1369.17
1413.00
1455.92
1499.00
1837.00
m/z
